# One step at a time. Shaping consensus on research priorities and terminology in telehealth in musculoskeletal pain: an international modified e-Delphi study

**DOI:** 10.1186/s12891-023-06866-0

**Published:** 2023-10-03

**Authors:** Junior V. Fandim, Rana S. Hinman, Cecilie K. Øverås, Saurab Sharma, Joletta Belton, Vinícius C. Oliveira, Blake F. Dear, Romy Parker, Babita Ghai, Kim L. Bennell, Paulo Ferreira, Jan Hartvigsen, Bruno T. Saragiotto

**Affiliations:** 1 Masters and Doctoral Program in Physical Therapy, Universidade Cidade de São Paulo (UNICID), Rua Cesário Galeno, 448, Tatuapé, CEP 03071-000 São Paulo, SP Brasil; 2 Department of Physiotherapy, Centre for Health, Exercise & Sports Medicine, University of Melbourne, Parkville, Australia; 3 Department of Public Health and Nursing, NTNU - Norwegian University of Science and Technology, Trondheim, Norway; 4 Department of Sports Science and Clinical Biomechanics, University of Southern Denmark, Odense, Denmark; 5 Centre for Pain IMPACT, Neuroscience Research Australia, Sydney, Australia; 6 School of Health Sciences, Faculty of Medicine, University of New South Wales, Sydney, Australia; 7 IASP Global Alliance of Partners for Pain Advocacy (GAPPA), Washington D.C, USA; 8 Postgraduate Program in Rehabilitation and Functional Performance, Universidade Federal Dos Vales Do Jequitinhonha E Mucuri (UFVJM), Diamantina, MG Brazil; 9 School of Psychological Sciences, Macquarie University, Macquarie Park, Australia; 10 Department of Anaesthesia and Perioperative Medicine, Faculty of Health Sciences, University of Cape Town, Cape Town, South Africa; 11 Department of Anaesthesia and Intensive Care, Post Graduate Institute of Medical Education and Research, Chandigarh, India; 12 Faculty of Medicine and Health, The University of Sydney, Charles Perkins Centre, Sydney School of Health Sciences, Camperdown, NSW Australia; 13 Chiropractic Knowledge Hub, Odense, Denmark; 14 Discipline of Physiotherapy, Graduate School of Health, Faculty of Health, University of Technology Sydney, Sydney, Australia

**Keywords:** Telehealth, Telerehabilitation, Delphi technique, Research priorities, Terminology, Musculoskeletal pain

## Abstract

**Background:**

Telehealth has emerged as an alternative model for treatment delivery and has become an important component of health service delivery. However, there is inconsistency in the use of terminologies and a lack of research priorities in telehealth in musculoskeletal pain. The purpose of this international, multidisciplinary expert panel assembled in a modified three-round e-Delphi survey is to achieve a consensus on research priorities and for the standard terminology for musculoskeletal pain telehealth practice.

**Methods:**

In this international modified e-Delphi survey, we invited an expert panel consisting of researchers, clinicians, consumer representatives, industry partners, healthcare managers, and policymakers to participate in a three-round e-Delphi. Expert panels were identified through the Expertscape website, PubMed database, social media, and a snowball approach. In Round 1, potential research priorities and terminologies were presented to panel members. Panel members rated the agreement of each research priority on a 5-point Likert scale and an 11-point numerical scale, and each terminology on a 5-point Likert scale for the "telehealth in musculoskeletal pain " field over rounds. At least 80% of the panel members were required to agree to be deemed a consensus. We analyzed the data descriptively and assessed the stability of the results using the Wilcoxon matched-pairs signed rank test.

**Results:**

We performed an international e-Delphi survey from February to August 2022. Of 694 invited people, 160 panel members participated in the first round, 133 in the second round (83% retention), and 134 in the third round (84% retention). Most of the panel members were researchers 76 (47%), clinicians 57 (36%), and consumer representatives 9 (6%) of both genders especially from Brazil 31 (19%), India 22 (14%), and Australia 19 (12%) in the first round. The panel identified fourteen telehealth research priorities spanned topics including the development of strategies using information and communication technology, telehealth implementation services, the effectiveness and cost-effectiveness of telehealth interventions, equity of telehealth interventions, qualitative research and eHealth literacy in musculoskeletal pain conditions from an initial list of 20 research priorities. The consensus was reached for "digital health" and "telehealth" as standard terminologies from an initial list of 37 terminologies.

**Conclusion:**

An international, multidisciplinary expert consensus recommends that future research should consider the 14 research priorities for telehealth musculoskeletal pain reached. Additionally, the terms digital health and telehealth as the most appropriate terminologies to be used in musculoskeletal telehealth research.

**Register:**

Open Science Framework (https://osf.io/tqmz2/).

**Supplementary Information:**

The online version contains supplementary material available at 10.1186/s12891-023-06866-0.

## Introduction

The World Health Organization (WHO) defines telehealth as the delivery of health care services where patients and providers are separated by distance using information and communication technologies [1]. Telehealth is considered an important component of the modern healthcare system [2], and is a particularly valuable model for providing healthcare for long-term conditions and disadvantaged populations [3]. During the COVID-19 pandemic, healthcare systems used telehealth as a strategy to ensure continuous care and overcome access and geographic barriers [3–5].

Musculoskeletal disorders, such as low back pain and osteoarthritis, are major health problems and are the second greatest contributor to disability requiring rehabilitation worldwide [6–8]. Telehealth is suitable for delivering a wide range of healthcare services in both synchronous and asynchronous modes, through technologies increasing the chance of access to care in remote regions [3]. Telehealth has been reported as effective in chronic pain management, with benefits being recognized by stakeholders [3, 9].

Identifying the research priorities related to telehealth in musculoskeletal conditions is necessary to guide researchers in addressing the most important questions and producing high-quality evidence that meets community needs [10]. Previous research agendas for telehealth have been formulated to guide the development of multi-level telehealth studies in multiple contexts [11–13]. However, none of the previous studies have considered a broader participation from different stakeholders with diverse geographic and socioeconomic conditions or using a systematic method of consensus, such as a Delphi study. In addition, the telehealth terminologies currently available (eg, telecare, telemedicine) are inconsistent, often leading to confusion, lack of clarity in research studies, and inappropriate decision-making [14–16]. Thus, consensus on research priorities and telehealth terminologies are urgently needed in the musculoskeletal pain field. We used a modified e-Delphi approach to tackle two goals in one:

- To reach a consensus on research priorities for telehealth in musculoskeletal conditions;
- To reach a consensus on the best terminologies to be used for telehealth in musculoskeletal practice.

## Methods

### Protocol and registration

The protocol of this international modified e-Delphi study survey was registered prospectively and is available to the public on the Open Science Framework (https://osf.io/tqmz2/).

### Study design

This was a three-round international modified e-Delphi survey. We used an electronic process model that permits international involvement, reducing geographic barriers and providing greater accessibility for Delphi Panel Members to participate [17, 18]. We used the Guidance on Conducting and Reporting Delphi Studies (CREDES) [19] and followed the proposed guidelines for developing surveys using the Delphi method [17]. We used the term “telehealth” to describe the context of this e-Delphi survey because it is considered an umbrella term [1, 20]. and provides a common understanding between all interested parties (See Supplementary file 1 A for operational definitions in this study).

### Ethical considerations

Participants were informed about the objective and procedures of the study and provided informed consent before completing the first e-Delphi round. Ethical approval for the study was granted by the Research Ethics Committee at Universidade da Cidade de São Paulo (40705620.5.0000.0064).

### Establishment of an international steering committee

An International Steering Committee was formed to initiate, support, and guide the study [21, 22]. The members of the International Steering Committee were selected to represent a diversity of genders, disciplines, expertise, and geographical areas and included researchers with expertise in telehealth and musculoskeletal pain. The 11 members of the Steering Committee were from Australia, Brazil, Denmark, India, Nepal, Norway, South Africa and the USA. The Steering Committee was also invited to participate as panel members and complete the survey. The leading research authors (JF and BS) coordinated the day-to-day management and did not participate as panel members during the study.

### Patient and public involvement

We invited researchers, consumer representatives, clinicians, healthcare managers, industry partners, and policymakers as panel members for this e-Delphi survey to ensure we captured a wide breadth of perspectives and views. A patient partner (JB) with lived experience of musculoskeletal pain and treatment was a member of the International Steering Committee and provided insights into survey design, research content, language, and dissemination plans. We also sought informal feedback from researchers, telehealth clinicians and a patient with musculoskeletal pain to provide input on survey design, survey content, and language.

### Selection of e-Delphi panel members

Panel members were identified from October 2021 to March 2022. Panel members were composed of English-speaking musculoskeletal pain telehealth researchers/thought leaders, telehealth clinicians, consumer representatives, healthcare managers, industry partners, and policymakers. Eligibility criteria were:

- Musculoskeletal pain telehealth researchers/thought leaders listed on the Expertscape platform under the topics "Internet-based intervention", "telerehabilitation" "telemedicine", "remote consultation" involved in musculoskeletal pain telehealth research—i.e., first or last author for at least one published clinical trial or systematic review of telehealth indexed in PubMed in the last 10 years). PubMed search in Supplementary file 2;
- Clinicians registered to practice in their home country and have clinical experience with an average of 12 patients with musculoskeletal pain managed via telehealth over the last 12 months;
- Consumer representatives, advocates, patients, individuals, health service users, caregivers and family members with any personal experience of musculoskeletal pain in telehealth-delivered care or participated in research on telehealth for musculoskeletal pain;
- Industry partners or representatives of organizations or other entities who worked in the development of telehealth solutions related to musculoskeletal pain care;
- Healthcare managers employed within a healthcare organization (e.g., public health units);
- Policymakers (e.g., scientific advisors, civil servants, ministers, or politicians) who formulate, manage, or implement public or private health policy on health programs and/or services at any level of government (e.g., local, state, provincial, national, or international) related to telehealth-delivered care or telehealth research.

We also adopted a snowball sampling strategy approach where the International Steering Committee and e-Delphi panel members [23] recommended potential eligible candidates, and also shared the study invitation among your contacts and social media (See Supplementary file 1 B-C). We also invited professional organizations and used social media with weekly advertisements (i.e., Facebook, Instagram, LinkedIn, and Twitter) to recruit additional participants between February and March 2022. We contacted all potential, recommended and subscribed candidates by personalized invitations through e-mail, followed by a set of questions at the start of the e-Delphi survey to confirm eligibility and to advance to the sections.

#### The sample size for the Delphi panel members

There is no consensus on the sample size for an e-Delphi survey [24, 25]. Sample sizes typically vary due to complexity, characteristics of participants (e.g., homogeneity) and field [18, 26]. Previous Delphi surveys have used a sample size of between 23 to 603 participants [24, 27]. Thus, estimating a response rate in the first round of 20 to 40% [24, 28, 29], we planned to invite a minimum of 300 participants and include at least 60 participants in the first round.

### Generation of a draft list of potential terminologies and priorities

We generated a list of research priorities and terminologies in three phases, guided by the International Steering Committee. We developed a list of potential research priorities and terminologies items by researching previously published studies [2, 11, 13, 16, 30–32]. The list of research priorities were developed from a search for research priorities developed by The Partnership to Enable Optimal Primary Health Care by Leveraging Digital Media in Musculoskeletal Health Meeting (The PEOPLE Meeting), The Transatlantic Telehealth Research Network and relevant published studies and the terminologies was developed from a search of terms provided in telehealth literature and scoping review [2, 11, 13, 16, 30–32]. The local team presented the draft list of research priorities and terminologies for discussion and feedback in a research group (15 researchers), made modifications to the list, and discussed and ratified the final draft list with the International Steering Committee.

### e-Delphi procedures

This international modified e-Delphi survey was conducted in three rounds with open and closed-ended questions. We performed a pilot test of the study procedures with members of the International Steering Committee, Brazilian researchers and clinicians, and native English speakers over one week [18].

We then invited participants to become members of the Delphi panel using an individualised e-mail invitation containing the link to the survey (directing to round 1) using the Typeform® platform [33]. The e-Delphi survey was performed in three rounds (four to eight weeks apart) with an approximate four-week duration to complete each round of the survey. A reminder was sent to all panel members two weeks after the beginning of each round. Potential panel members who were unable to participate in the first or second round were invited to participate in the subsequent round [34]. Panel members were not able to submit the survey without answering the required questions. At the start of rounds 2 and 3, feedback on panel responses from the previous round was presented.

#### First-round

The survey consisted of three sections: 1) Panel member’s background information (e.g., name, country of residence, main stakeholder group) and queries to confirm eligibility; 2) Research priorities for telehealth in musculoskeletal pain research, including the importance of each research priority; and 3) Standard terminologies for telehealth in musculoskeletal pain research. Participants also had the opportunity to suggest new priorities/terms or edit those presented in an open question at the end of each section.

 Panel members were asked to rate their agreement for research priorities with a 5-point Likert scale (from "strongly disagree" to "strongly agree") [35] and importance with an 11-point numerical scale (from "0 = lower priority" to "10 = higher priority"). For standard terminologies, panel members were asked to rate their familiarity on a 4-point Likert scale (from "not at all familiar" to "very familiar"; only in the first round) and to rate their agreement with a 5-point Likert scale (from "strongly disagree" to "strongly agree") [35]. For consensus, we established an a priori cut-off as at least 80% of panel members rated an item as agree/strongly agree [36, 37]. We classified the level of agreement into four groups [38, 39]: 1) Strong agreement (80% of ratings as agree/strongly agree); 2) Moderate agreement (70–80% of rated as agree/strongly agree); 3) Low agreement (50–70% of rated as agree/strongly agree) and 4) No agreement (< 50% of rated as agree/strongly agree) [38, 39]. Items classified as *no agreement* were considered unnecessary and excluded from subsequent rounds. Items classified as *low* or *moderate agreement* were retained for the next round. Items classified as *strong agreement* were automatically included in the final list [39].

#### Second-round

Panel members were asked to rate their level of agreement for new items and items retained from Round 1, using the same rating scales from Round 1. We again classified the level of agreement for each item from *strong agreement* to *no agreement*. Items classified as *no agreement* were excluded from the third round. Items classified as *low* or *moderate agreement* were retained for the third round. Items classified as *strong agreement* were automatically included in the final list [39]. We provided open-text boxes to panel members to propose re-editing and/or merging of research priorities that had overlapping meanings.

#### Third-round.

Panel members were asked to rate the level of agreement for each retained research priority and terminology generated from round two using the same rating scales as for round 1. There were open-text boxes to propose editing and/or merging of similar research priorities and terminologies. We again classified the level of agreement for each item from *strong agreement* to *no agreement*. Items classified as *strong agreement* (i.e. achieving our a priori definition of consensus) were included in the final list.

## Data analysis

We performed descriptive data analysis using measures of central tendency (mean), variability (SD), and absolute and relative frequencies to demonstrate panel members´ demographic characteristics and group responses in all rounds, using Microsoft Excel for Office 365 (Microsoft Corporation, Redmond, WA, USA) and Statistical Package for the Social Sciences (IBM® SPSS Statistics for Windows, Version 19.0, Armonk, NY, USA). The research priorities and terminologies were presented by the level of importance and agreement from highest to lowest rated responses. The stability of the results between rounds two and three was assessed using the Wilcoxon matched-pairs signed rank test [40, 41]. Statistical significance was set at ≤ 0.05. We considered the results stable when there was no significant difference between the items [40, 41]. In addition, a post hoc sensitivity analysis was performed to account the results between rounds with Non-International Steering Committee panel members and compared the results of all panel members and Non-International Steering Committee panel members.

## Results

**Fig. 1:**
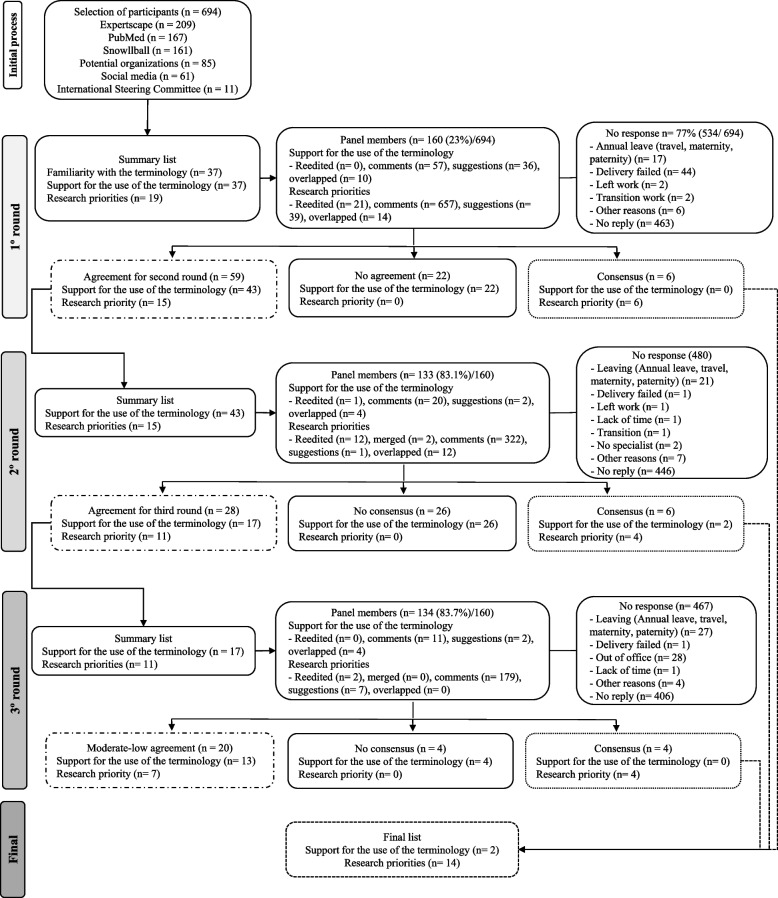
Flowchart of each round in the participation process researchers and panel members formed by participants in e-Delphi survey to identify the terminology and research priorities in telehealth in musculoskeletal pain research

The e-Delphi study was conducted over 7 months, with the first round running from February 1 to March 25, 2022, the second round from May 2 to May 31, 2022, and the third round from July 1 to August 5, 2022. 694 potential panel members were invited, including 455 researchers/thought leaders, 83 clinicians, 11 consumer representatives, 10 healthcare managers, 8 industry partners, 86 policymakers, and 41 unclassified respondents. A total of 160 people agreed to participate in the first round of the e-Delphi survey (Fig. 1). A total of 194 panel members participated in at least one round, 92 panel members participated in all three rounds, 46 panel members participated in at least two rounds, and 56 panel members participated in one round only. The panel members’ retention rate from Round 1 was 133 (83%) participants in the second round and 134 (84%) participants in the third round. The demographic characteristics of the participants in each round are summarized in Table 1.

**Table 1:** Characteristic of panel members of the e-Delphi study rounds

Characteristics	First round (*n* = 160)	Second round (*n* = 133)	Third round (*n* = 134)	All rounds (*n* = 92)
Panel members (%)^a^				
Researcher/thought leader	76 (47)	71 (53)	75 (56)	52 (56)
Clinician	57 (36)	46 (34)	44 (33)	27 (29)
Consumer representative	9 (6)	9 (7)	8 (6)	8 (9)
Healthcare manager	9 (6)	2 (1)	4 (3)	2 (2)
Industry partner	7 (4)	3 (2)	2 (1)	2 (2)
Policymaker	2 (1)	2 (1)	1 (1)	1 (1)
Highest level of education (%)^a^				
PhD	65 (41)	62 (47)	69 (51)	41 (45)
Master's Degree	59 (37)	49 (37)	43 (32)	35 (38)
Bachelor’s Degree	22 (14)	16 (12)	15 (11)	12 (13)
University (other)	9 (6)	0 (0)	1 (1)	0
Diploma/Certificate/Apprenticeship	2 (1)	3 (2)	3 (2)	2 (2)
Others	3 (2)	3 (2)	3 (2)	2 (2)
Gender (%)^a^				
Woman	76 (47)	75 (56)	74 (55)	48 (52)
Man	84 (52)	58 (44)	60 (45)	44 (48)
Age (mean/SD) ^a^				
	42 (11)	42 (11)	44 (11)	43 (11)
Country of residence (%)^a^				
Brazil	31 (19)	24 (18)	23 (17)	17 (18)
India	22 (14)	11 (8)	10 (7)	6 (6)
Australia	19 (12)	15 (11)	16 (12)	10 (11)
United Kingdom	14 (9)	11 (8)	10 (7)	7 (8)
Canada	13 (8)	11 (8)	11 (8)	9 (10)
United States of America	10 (6)	9 (7)	12 (9)	7 (8)
Denmark	6 (4)	4 (3)	5 (4)	1 (1)
Italy	5 (3)	5 (4)	6 (4)	5 (5)
Netherlands	5 (3)	6 (4)	5 (4)	3 (3)
Norway	5 (3)	4 (3)	4 (3)	3 (3)
Spain	4 (2)	4 (3)	5 (4)	4 (4)
Nepal	3 (2)	4 (3)	2 (1)	1 (1)
Chile	3 (2)	4 (3)	3 (2)	3 (3)
Switzerland	2 (1)	2 (1)	2 (1)	1 (1)
Nigeria	2 (1)	1 (1)	1 (1)	1 (1)
Poland	2 (1)	2 (1)	2 (1)	2 (2)
Portugal	2 (1)	1 (1)	1 (1)	1 (1)
Germany	2 (1)	2 (1)	3 (2)	1 (1)
Colombia	1 (1)	2 (1)	2 (1)	1 (1)
China	1 (1)	1 (1)	1 (1)	1 (1)
Turkey	1 (1)	2 (1)	2 (1)	1 (1)
Angola	1 (1)	0 (0)	0 (0)	0 (0)
Belgium	1 (1)	1 (1)	1 (1)	1 (1)
Indonesia	1 (1)	1 (1)	1 (1)	1 (1)
Saint Lucia	1 (1)	0 (0)	0 (0)	0 (0)
Ireland	1 (1)	1 (1)	2 (1)	1 (1)
South Africa	1 (1)	2 (1)	1 (1)	0 (0)
Sweden	1 (1)	1 (1)	0 (0)	0 (0)
Japan	0 (0)	1 (1)	1 (1)	1 (1)
Agentina	0 (0)	1 (1)	1 (1)	0 (0)
Israel	0 (0)	0 (0)	1 (1)	0 (0)
Income economies (%)^a^				
High-income economies	96 (60)	85 (64)	91 (68)	64 (70)
Upper-middle-income economies	35 (22)	31 (23)	30 (22)	19 (21)
Lower-middle-income economies	29 (18)	17 (13)	13 (10)	9 (10)
Low-income economies	0 (0)	0 (0)	0 (0)	0 (0)
Researchers^a^				
Area of research (%)^b^				
Back pain	37 (54)	31 (44)	35 (49)	26 (50)
Acute and subacute pain	11 (16)	12 (17)	12 (17)	11 (21)
Chronic pain	38 (55)	38 (53)	39 (55)	31 (60)
Foot and/or ankle pain	5 (7)	2 (3)	2 (3)	1 (2)
Hip and/or knee pain	19 (27)	15 (21)	17 (24)	12 (23)
Neck pain	13 (19)	12 (17)	12 (17)	10 (19)
Shoulder, upper limb and/or hand pain	17 (25)	10 (14)	11 (15)	9 (17)
Thoracic spine pain	6 (9)	5 (7)	5 (7)	3 (6)
Others	12 (17)	12 (17)	12 (17)	6 (11)
Main treatment approach researched in telehealth (%)				
Behavioral or psychological therapies	13 (17)	9 (13)	7 (10)	5 (10)
Education	5 (7)	1 (1)	2 (3)	1 (2)
Exercise & physical activity	18 (24)	25 (35)	24 (33)	20 (38)
Electronic devices development	2 (3)	1 (1)	1 (1)	1 (2)
Public/population health	1 (1)	2 (3)	3 (4)	1 (2)
Self-management	12 (16)	14 (20)	15 (20)	10 (19)
Multi-component (e.g., education and exercise)	24 (32)	17 (24)	18 (25)	12 (23)
Others	1 (1)	2 (3)	3 (4)	2 (4)
Telehealth modality(s) worked (%)^b^				
E-mail	10 (13)	4 (6)	5 (7)	3 (6)
SMS/Text messages	19 (26)	14 (20)	17 (24)	11 (21)
Smartphone application	38 (51)	34 (48)	30 (42)	25 (48)
Video conference	35 (47)	27 (38)	32 (44)	20 (38)
Website platform	35 (47)	39 (55)	36 (50)	26 (50)
Telephone call	21 (28)	19 (27)	24 (33)	15 (29)
Others	1 (1)	2 (3)	2 (3)	2 (4)
Consumer representatives^a^				
Musculoskeletal conditions which received telehealth (%)				
Back pain	3 (33)	3 (37)	3 (43)	3 (43)
Acute and subacute pain	0 (0)	0 (0)	0 (0)	0 (0)
Chronic pain	5 (55)	4 (50)	3 (43)	3 (43)
Foot and/or ankle pain	1 (11)	0 (0)	0 (0)	0 (0)
Hip and/or knee pain	4 (44)	2 (25)	2 (29)	2 (29)
Neck pain	2 (22)	1 (12)	1 (14)	1 (14)
Shoulder, upper limb and/or hand pain	4 (44)	2 (25)	2 (29)	2 (29)
Thoracic spine pain	0 (0)	1 (12)	0 (0)	0 (0)
Others	0 (0)	0 (0)	0 (0)	0 (0)
Telehealth modality(s) experienced (%)				
E-mail	2 (22)	4 (44)	3 (37)	3 (37)
SMS/Text messages	2 (22)	1 (11)	1 (12)	1 (12)
Smartphone application	2 (22)	3 (33)	3 (37)	3 (37)
Video conference	4 (44)	1 (11)	1 (12)	1 (12)
Website platform	4 (44)	3 (33)	3 (37)	3 (37)
Telephone call	4 (44)	7 (78)	6 (75)	6 (75)
Others	0 (0)	0 (0)	0 (0)	0 (0)
Industry partners^a^				
Area of work (%)^b^				
Back pain	5 (71)	2 (67)	2 (100)	2 (100)
Acute and subacute pain	2 (29)	0 (0)	0 (0)	2 (100)
Chronic pain	5 (71)	2 (67)	1 (50)	1 (50)
Foot and/or ankle pain	1 (14)	0 (0)	0 (0)	2 (100)
Hip and/or knee pain	3 (43)	2 (67)	1 (50)	1 (50)
Neck pain	2 (29)	0 (0)	0 (0)	0 (0)
Shoulder, upper limb and/or hand pain	1 (14)	0 (0)	0 (0)	0 (0)
Thoracic spine pain	1 (14)	0 (0)	0 (0)	0 (0)
Others	0 (0)	1 (33)	0 (0)	0 (0)
Main treatment approach worked in telehealth (%)				
Behavioral or psychological therapies	0 (0)	0 (0)	0 (0)	0 (0)
Education	0 (0)	0 (0)	0 (0)	0 (0)
Exercise & physical activity	1 (14)	0 (0)	0 (0)	0 (0)
Electronic devices development	1 (14)	1 (33)	1 (50)	1 (50)
Public/population health	1 (14)	0 (0)	0 (0)	0 (0)
Self-management	1 (14)	0 (0)	0 (0)	0 (0)
Multi-component (e.g., education and exercise)	3 (43)	1 (33)	1 (50)	1 (50)
Others	1 (14)	1 (33)	0 (0)	0 (0)
Telehealth modality(s) experienced (%)^ab^				
E-mail	2 (33)	1 (33)	0 (0)	0 (0)
SMS/Text messages	3 (50)	1 (33)	0 (0)	0 (0)
Smartphone application	5 (83)	2 (67)	1 (50)	1 (50)
Video conference	4 (67)	2 (67)	1 (50)	1 (50)
Website platform	4 (67)	1 (33)	0 (0)	0 (0)
Telephone call	2 (33)	2 (67)	1 (50)	1 (50)
Others	0 (0)	0 (0)	0 (0)	0 (0)

Continuous data presented as mean (SD) and categorical as frequency and proportion (%); Information unavailable for some panel members^a^; Panel members could select more than one response^b^

### Results for telehealth terminology and priorities

#### Results for research priorities

In the first round, 20 research priorities were presented to the panel members. A total of six (30%) research priorities met the criteria for strong agreement (> 80%) and were included in the final consensus list. Ten (50%) research priorities had a moderate agreement (70–80%) and four (20%) priorities had a low agreement (50–70%) and all of these were considered again in the next round. No priorities were rejected due to no agreement (< 50%) (See Supplementary file 3 A-D). One new research priority was proposed and 657 general comments were received, including edits and suggestions to the current priorities that were revised.

In the second round, 15 research priorities were considered and four (26%) met the criteria for strong agreement (> 80%) and were included in the final consensus list. Five (33%) research priorities had a moderate agreement (70–80%) and six (40%) priorities had a low agreement (50–70%), all of which proceeded to round 3 for further consideration. No priorities were rejected due to no agreement (< 50%) (See Supplementary file 4 A-D). One new research priority was suggested, and two were merged followed by 322 comments.

**Fig. 2:**
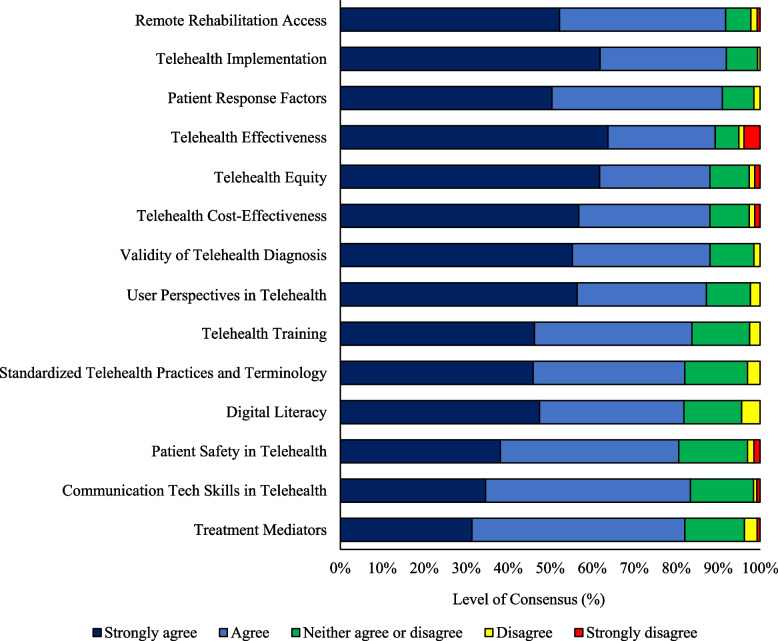
Final list of telehealth research priorities ranked from highest to lowest. Research Priority Abbreviated: 1) Research and development of strategies for using information and communication technology to create and facilitate access rehabilitation care for individuals with musculoskeletal with difficulty in transportation, financial resources or living in different regions (such as remote, rural, and others); 2) Research about how to implement telehealth services at the user, clinician and health system level; 3) Identification of patient characteristics that affect response to treatments delivered by telehealth; 4) Effectiveness of treatment approaches delivered via telehealth in the management of musculoskeletal conditions; 5) Equity research on interventions to improve access, treatment or clinical outcomes to telehealth services for disadvantaged or historically underserved populations with musculoskeletal conditions; 6) The cost-effectiveness of telehealth treatments for musculoskeletal conditions; 7) Research on reliability and validity of clinical assessment and diagnostic tests administered via telehealth (compared to in-person testing) in individuals with musculoskeletal conditions; 8) Qualitative telehealth research involving all end-users to determine perceptions, barriers, and enablers in the management of musculoskeletal conditions; 9) Design and evaluation of curricula to train students and health care practitioners in the provision of telehealth that conforms to core capability frameworks; 10) Standardization of telehealth-related terms and the development of frameworks and guidelines for musculoskeletal telehealth practice; 11) Research on health literacy, eHealth literacy, technology literacy and identifying relevant factors for patients with musculoskeletal conditions engaging in telehealth (eg, barriers and facilitators); 12) Investigation of adverse events and patient safety during telehealth encounters for musculoskeletal conditions; 13) Research that examines the specific contribution of communication and information technology and digital skills to the effectiveness of telehealth treatments in musculoskeletal conditions; 14) Identification of mediators contributing to the effects of telehealth-delivered treatments

In the third round, 11 research priorities were considered. Four (36%) met the prior criteria for strong agreement (> 80%) and were included in the final consensus list (Fig. 2). Five (45%) priorities had a moderate agreement (70–80%) and two (18%) priorities had a low agreement (50–70%). No priorities were rejected due to no agreement (< 50%). Panel members provided 179 general comments during this round (See Supplementary file 5 A-D). Overall, panel members reached a consensus on 14 out of 20 telehealth research priorities from the initially proposed list in the first round. The final list of 14 telehealth research priorities in musculoskeletal pain is provided in Fig. 2 and Table 2. Further information regarding the final list is available (See Supplementary file 6 A-C).

**Table 2:** Final list terminologies and telehealth research priorities for musculoskeletal pain ordered from highest to lowest importance by the panel members

		Level of agreement (%)	Level of importance (mean/SD)
*Telehealth terminologies*			
1	Digital health	84%	-
2	Telehealth	82%	-
*Telehealth research priorities*			
1	Remote Rehab﻿ilitation Access: Research and development of strategies for using information and communication technology to create and facilitate access rehabilitation care for individuals with musculoskeletal with difficulty in transportation, financial resources or living in different regions (such as remote, rural, and others)	92%	8 (1)
	Research studies and development of strategies to use information and communication technology to create and facilitate access to rehabilitation care for individuals with musculoskeletal conditions (especially chronic musculoskeletal conditions) with difficult to access in-person healthcare service such as transportation difficulties, time difficulties, limited financial resources, or living in different regions (such as remote, rural, and others)		
2	Telehealth Implementation: Research about how to implement telehealth services at the user, clinician and health system level	92%	8 (2)
	Studies on implementation strategies of telehealth at the user (i.e., patient, clinician and consumers), workforce and health system levels (e.g., centers, hospitals and other organizations) with patient/consumer partners involvement. It comprises the science of implementation, following consideration of key components, strategies, and methods for promoting the systematic adoption of evidence-based telehealth interventions into practice and policy to improve health. This includes implementation trials, randomized controlled trials, hybrid designs, mixed methods, qualitative studies, and any study design aiming at understanding implementation context or implementing a new telehealth strategy		
3	Patient Response Factors: Identification of patient characteristics that affect response to treatments delivered by telehealth	91%	8 (2)
	Studies identifying moderators (e.g., clinical, demographic, and psychological status) that interact with telehealth treatments to change outcomes in individuals with musculoskeletal pain conditions		
4	Telehealth Effectiveness: Effectiveness of treatment approaches delivered via telehealth in the management of musculoskeletal conditions	89%	8 (2)
	Studies testing the clinical effectiveness of interventions (e.g., education, psychological interventions, exercise, etc.), through telehealth or telecommunication platforms (e.g., chat, apps, website), with any delivery method (synchronous and asynchronous), mode of communication (e.g., audio, video, or text), degree of care (e.g., self-guided, tailoring intervention or blended care), by any treatment provider (e.g., physiotherapist, pain physician/clinician or multidisciplinary staff) considering different setting, and follow-up periods (short, medium and long), using research designs, such as randomized controlled trials, mixed methods, or systematic reviews		
5	Telehealth ﻿Equity: Equity research on interventions to improve access, treatment or clinical outcomes to telehealth services for disadvantaged or historically underserved populations with musculoskeletal conditions	88%	8 (2)
	Studies to identify and assess the effects of telehealth interventions to improve access, treatment or clinical outcomes in disadvantaged or historically underserved populations who may suffer from health inequity and inequalities based on PROGRESS-Plus characteristics (i.e., place of residence, race/ethnicity/ culture/language, occupation, gender/sex, religion, education, socioeconomic status, social capital, age, disability, and sexual orientation)		
6	Telehealth Cost-Effectiveness: The cost-effectiveness of telehealth treatments for musculoskeletal conditions	88%	8 (2)
	Randomized controlled trials or other designs including economic evaluations and cost-effectiveness research for telehealth interventions for musculoskeletal conditions considering different settings and income levels		
7	Validity of Telehealth Diagnosis: Research on reliability and validity of clinical assessment and diagnostic tests administered via telehealth (compared to in-person testing) in individuals with musculoskeletal conditions	88%	8 (2)
	Studies investigating the reliability and validity of *clinical assessment and* diagnostic tests administered via telehealth (compared to in-person testing) in individuals with musculoskeletal conditions		
8	﻿User Perspectives in Telehealth: Qualitative telehealth research involving all end-users to determine perceptions, barriers, and enablers in the management of musculoskeletal conditions	87%	8 (2)
	Qualitative research in telehealth involving all end-users (e., g patient, patient partners, healthcare professionals) to explore and determine in depth the perspectives, facilitators, and barriers in the acceptance, decline and engagement of individuals with musculoskeletal conditions and healthcare professionals. And also explore the extent to which new evidence on the perspectives, barriers and facilitators of the use of communication and information technology is currently being addressed to improve acceptability and engagement in the management of musculoskeletal conditions		
9	Telehealth Training: Design and evaluation of curricula to train students and health care practitioners in the provision of telehealth that conforms to core capability frameworks	84%	8 (2)
	Development of general telehealth curricula in institutions that offer any health courses for training in the ethical aspects and core capability framework (e.g., patient privacy, care planning, technology skills and delivery) for healthcare practitioners using information and communication technology to provide any treatment safely to the patient		
10	Standardized Telehealth Practices and Terminology: Standardization of telehealth-related terms and the development of frameworks and guidelines for musculoskeletal telehealth practice	82%	8 (2)
	Identify, maintain, and update suitable terminologies related to telehealth to ensure service delivery, and develop guidelines for standard practices, and safe telehealth treatment for individuals with musculoskeletal pain conditions		
11	Digital Literacy: Research on health literacy, eHealth literacy, technology literacy and identifying relevant factors for patients with musculoskeletal conditions engaging in telehealth (eg, barriers and facilitators)	82%	8 (2)
	Research to identify and understanding all relevant patient factors, such as health literacy, eHealth literacy, technology literacy or other characteristics that may determine, influence or predict patients' experience of telehealth service (e.g., individual, personal, contextual, organizational and other factors)		
12	Patient Safety in Telehealth: Investigation of adverse events and patient safety during telehealth encounters for musculoskeletal conditions	81%	8 (2)
	Studies measuring or investigating ways to measure adverse events at a distance, as well as studies investigating medical, physical and emotional patient safety and ways to deal with urgencies during remote encounters		
13	Communication Tech Skills in Telehealth: Research that examines the specific contribution of communication and information technology and digital skills to the effectiveness of telehealth treatments in musculoskeletal conditions	83%	7 (2)
	Mixed methods research that examines specific components of telehealth communication and information technology (e.g., SMS, telephone or video), digital skills and their possible impact on care and health outcomes for individuals with musculoskeletal pain conditions		
14	Treatment Mediators: Identification of mediators contributing to the effects of telehealth-delivered treatments	82%	7 (2)
	Development of theory-based studies or mediation analysis of randomized controlled trials that help identify, explain, or contribute to the effects of telehealth-delivered treatments in the outcomes of individuals with musculoskeletal pain conditions (e.g., increases in self-efficacy, social support, or therapeutic alliance)		

Level of agreement 5-point Likert scale; level of importance a 11-point numerical scale; standard deviation (SD)

#### Consensus on a standard terminology

In the first round, none of the 37 terminologies met a priori criteria for strong agreement/consensus (> 80%). Four (11%) terminologies had a moderate agreement (70–80%) and 11 (30%) terminologies had low agreement (50–70%), which were all considered in the next round. Twenty-two (59%) terminologies were rejected from further rounds due to no agreement (< 50%) (See Supplementary file 7 A-D). In addition, 28 new terminologies were suggested by the panel members to be included in round 2.

In the second round, two (4.6%) terminologies "*Digital health*" and "*Telehealth*" met the prior criteria for strong agreement (> 80%) and were included in the final list. Five (12%) terminologies had a moderate agreement (70–80%) and 10 (23%) terminologies had low agreement (50–70%). Twenty-six (60%) terminologies were rejected due to no agreement (< 50%) (See Supplementary file 8 A-C). Two new terminologies were suggested by the panel members to be included in round 3.

In the third round, no additional terminologies met our prior criteria for strong agreement (> 80%). Three (18%) terminologies had moderate agreement (70–80%), 10 (59%) terminologies had a low agreement (50–70%), and four (24%) terminologies were rejected due to no agreement (< 50%). See Supplementary file for further information on scoring across round three (Supplementary file 9 A-C). Overall, panel members reached a consensus on two out of 37 telehealth terminologies from the initially proposed list in the first round. The final consensus list included only "*digital health*" and "*telehealth*" as standardised terminologies.

For further information, all qualitative and quantitative information provided in the feedback to the panel members in each round is available (See Supplementary files 10, 11 and 12).

### Stability analysis

We conducted a stability analysis for research priorities and terminologies between Rounds 2 and 3. Stability was reached in six out of eight research priorities and 10 of the 15 terminologies (*p* > 0.05) (See Supplementary file 13).

### Sensitivity analysis

Robust evidence was also obtained after accounting for panel member participation in the sensitivity analysis. For research priorities, it was observed the research priority "Identification of patient characteristics" could have upgraded one level of agreement from moderate (80%) to strong agreement (81%) in the first round. Likewise, the research priority "Investigation of adverse events" could have downgraded one level from strong (81%) to moderate agreement (79%) in the third round. The first and third round analyses yielded similar results for terminologies. However, the terminology "Telehealth" could have had downgraded one level from strong (82%) to moderate agreement (80%) in the second round, returning to participate in the third round. See Supplementary file for further information for sensitivity analysis of research priorities and terminologies (Supplementary file 14 A-F).

## Discussion

### Summary of main findings

This international modified e-Delphi survey reached a consensus on research priorities and telehealth terminology for musculoskeletal pain research that can be used in all contexts. The panel members reached a consensus on 14 research priorities which are the development of strategies for using information and communication technology, telehealth implementation services, participant characteristics for telehealth treatment response, effectiveness and cost-effectiveness of telehealth interventions, equity research on telehealth interventions, qualitative research in end-users, reliability and validity of clinical assessment administered via telehealth, telehealth curricula design, telehealth frameworks and guidelines, eHealth literacy, telehealth patient safety, digital skills and telehealth treatment-related mediators. Further, the terminologies that reached consensus were "Digital health" and "Telehealth" from more than 40 terminologies presented over three rounds.

### Comparison with other e-Delphi surveys

While our survey shares some similar findings with the previous studies, it differs in terms of methodology study design, geographic regions and a diverse range of individual perspectives. In 2013, Li et al. [13] conducted a 2-day discussion with 26 participants from four key panel groups and generated four research priorities in digital health for chronic musculoskeletal conditions: (i) understanding the characteristics of individuals who are underserviced, (ii) barriers and ethical issues, (iii) development of technologies considering health and digital literacy, and (iv) improve the knowledge on the effectiveness of digital interventions. In 2018, Wethington et al. [32] conducted a practice consensus workshop model involving 60 participants following seven panel groups to generate research priorities in mobile health technologies and later-life pain. The authors identified 13 priorities classified into two categories: implications for research on mHealth among older adults with pain and implementation of technology and associated regulatory issues. However, the priorities were limited to only older adults with pain and there was no exploration of best-practice terminologies.

Our study was not limited to older adults, therefore can apply to research priorities across the lifespan, and there was no exploration of best-practice terminologies. We used the Delphi approach, which may reduce dominator bias, status effect and group pressure compared to other consensus studies [17, 42, 43] and the terminology chaos in the field. A strength of this e-Delphi survey approach is that we considered the voices of all panel members equally. Our study amplified the previous studies' findings and identified 14 research priorities that comprehensively cover the contextual needs and perspectives of a broader audience of interested parties from different geographic regions who are involved with musculoskeletal pain and economic incomes.

### Implications for practice and research

Developing a consensus on telehealth research priorities in the musculoskeletal pain population is essential for the development of an equitable research agenda that meets the needs of all interested parties. Funding agencies may use the research priorities in this e-Delphi study to guide funding decisions, aligning priorities with expert consensus and reducing the chance that priorities are chosen based on subjective, ad hoc objectives or that fail to respond to critical healthcare needs [10, 44]. Moving forward, it will remain important to involve patient partners, researchers, clinicians, industry partners, healthcare managers and policymakers to align research with relevant priorities, make research more efficient, and ensure that future telehealth research equitably addresses key health problems.

The widespread and inconsistent use of telehealth terminology is a problem known as "terminology chaos" in the field of psychology [45]. Our panel members reached a consensus that "Digital health" and "Telehealth" should be used as standard terminologies for the musculoskeletal field. Having a consensus for terminologies prevents patients from being overwhelmed by numerous terms and facilitates communication between clinicians and patients [15]. It also helps clinicians make appropriate healthcare decisions and avoid overestimating the available approaches, which can result in insufficient care. For researchers, it facilitates the communication of research findings in the community, helps develop a comprehensive search strategy and also aids clarity of scope and pooling of analysis in systematic reviews and meta-analyses [15].

### Strengths and limitations

Limitations of the study included the common problem of underrepresentation of policymakers, industry partners, and consumer representatives amongst our panel compared to other panel members (e.g., researchers) [46–48]. This can lead to possible underestimation or overestimation of researchers' and clinicians' insights which was a large proportion of the panel members over the other panel members. Further, the e-Delphi survey was developed in the English language, which limited the participation of non-English speakers. There is evidence that an e-Delphi survey provided in multiple languages may have a higher participation rate compared to an English language survey [49]. Further, we encountered barriers to contacting potential participants (e.g. email delivery failure, changing jobs or wrong email address, spam email problems) which means many potential experts were unable to participate and contribute their insights, reasoning, and contextual needs.

The strengths of the study include adherence following our prospective registered protocol and report considering the CREDES guideline, pilot testing phase to increase feasibility, comprehension, readability, usability, and validity, broad participation from 31 countries (including lower-middle-income, upper-middle-income and high-income countries), high panel member retention of up to 84% in later rounds, a multi-professional International Steering Committee with pain patient partners and researchers with expertise in telehealth and musculoskeletal pain, and the use of snowball and social media to advertise, update, and retain panel members. A total of 125 panel members participated in at least one round and social media received over 18,407 impressions, 1,779 video views and 281 engagements up to the end of the survey. Additionally, we conducted an evaluation of result stability between the second and third rounds, specifically examining the level of agreement regarding terminologies and research priorities. This approach allows us to account for the potential occurrence of chance results, as consensus can be achieved even with fluctuating responses across rounds.

## Conclusion

An international, multidisciplinary expert consensus recommends that future research should consider the 14 research priorities for telehealth musculoskeletal pain**.** Additionally, the terms "Digital health" and "Telehealth" are the most recommended terminologies by the panel members to be used in musculoskeletal telehealth research.

### Supplementary Information

**Additional file 1: Supplementary file 1. **A. Operational definitions. B. Social media survey information available for interested participants to sign up for the team members contact. C. Type form survey information and set of questions to potential participants to confirm eligibility and proceed to the sections.

**Additional file 2: Supplementary file 2.** Electronic search strategy at PubMed.

**Additional file 3: Supplementary file 3.  **A. First round panel members' group rating agreement on telehealth research priorities. B. First round panel members' rating ranked from highest to lowest importance on telehealth research priorities ranked from highest to lowest. C. First round panel members' group rating agreement in percent on telehealth research priorities ranked from highest to lowest. D. First round panel members' rate by income-level supporting the use of the term as standard terminology ranked from highest to lowest.

**Additional file 4: Supplementary file 4.** A. Second round panel members' group rating agreement on telehealth research priorities. B. Second round panel members' rating ranked from highest to lowest importance on telehealth research priorities ranked from highest to lowest. C. Second round panel members' group rating agreement in percent on telehealth research priorities ranked from highest to lowest. D. Second round panel members' rate by income-level supporting the use of the term as standard terminology ranked from highest to lowest.

**Additional file 5: Supplementary file 5.** A. Third round panel members' rating agreement in percent on telehealth research priorities ranked from highest to lowest. B. Third round panel members' rating ranked from highest to lowest importance on telehealth research priorities ranked from highest to lowest. C. Third round panel members' group rating agreement in percent on telehealth research priorities ranked from highest to lowest. D. Third round panel members' rate by income-level supporting the use of the term as standard terminology ranked from highest to lowest.

**Additional file 6: Supplementary file 6.** A. Final list of panel members' rating agreement level in percent on telehealth research priorities ranked from highest to lowest. B. Final list of panel members' rating group rating agreement level in percent on telehealth research priorities ranked from highest to lowest. C. Final list of panel members' rate by income-level supporting the use of the term as standard terminology ranked from highest to lowest.

**Additional file 7: Supplementary file 7.** A. Panel members' rating of how familiar they are with the term. B. First round panel members' rate agreement in percent on supporting the use of the term as standard terminology ranked from highest to lowest. C. First round panel members' group rate supporting the use of the term as standard terminology ranked from highest to lowest. D. First round panel members' rate by income-level supporting the use of the term as standard terminology ranked from highest to lowest.

**Additional file 8: Supplementary file 8.** A. Second round panel members' rate agreement in percent on supporting the use of the term as standard terminology ranked from highest to lowest. B. Second round panel members' group rate supporting the use of the term as standard terminology ranked from highest to lowest. C. Second round panel members' rate by income-level supporting the use of the term as standard terminology ranked from highest to lowest.

**Additional file 9: Supplementary file 9.** A. Third round panel members' rate agreement in percent on supporting the use of the term as standard terminology ranked from highest to lowest. B. Third round panel members' group rate supporting the use of the term as standard terminology ranked from highest to lowest. C. Third round panel members' rate by income-level supporting the use of the term as standard terminology ranked from highest to lowest.

**Additional file 10: Supplementary file 10.** Feedback with all the raw results of the first round.

**Additional file 11: Supplementary file 11.** Feedback with all the raw results of the second round.

**Additional file 12: Supplementary file 12.** Feedback with all the raw results of the third round.

**Additional file 13: Supplementary file 13.** A. Stability analysis of non-consensus terminologies items from the e-Delphi survey. B. Stability analysis of non-consensus research priorities items from the e-Delphi survey.

**Additional file 14: Supplementary file 14.** A. Sensitivity analysis of the first round for the support of the research priorities in musculoskeletal pain. B. Sensitivity analysis of the second round for the support of the research priorities in musculoskeletal pain. C. Sensitivity analysis of the third round for the support of the research priorities in musculoskeletal pain. D. Sensitivity analysis of the first round for the support of the use of the term as standard terminology in musculoskeletal pain. E. Sensitivity analysis of the second round for the support of the use of the term as standard terminology in musculoskeletal pain. F. Sensitivity analysis of the third round for the support of the use of the term as standard terminology in musculoskeletal pain.

## Data Availability

The study's raw data are available in the supplementary materials and also upon reasonable request from the corresponding author Junior Vitorino Fandim.
