# Supplementary material for: One step at a time. Shaping consensus on research priorities and terminology in telehealth in musculoskeletal pain: an international modified e-Delphi study
Source: BMC Musculoskelet Disord. 2023 Oct 3;24:783. doi: 10.1186/s12891-023-06866-0 (PMC10546725; doi:10.1186/s12891-023-06866-0)
Supplement: Supplementary file 1 — Additional file 1: Supplementary file 1. A. Operational definitions. B. Social media survey information available for interested participants to sign up for the team members contact. C. Type form survey information and set of questions to potential participants to confirm eligibility and proceed to the sections. [file 12891_2023_6866_MOESM1_ESM.docx]

| **Supplementary file 1 A. Operational definitions** |
| --- |
| **Telehealth** |
| The World Health Organization defines telehealth as the delivery of health care services where patients and providers are separated by distance [1]. Telehealth uses information and communication technologies for the exchange of information for the diagnosis and treatment of diseases and injuries, research and evaluation, and for the continuing education of health professionals, which is particularly valuable to support long-distance clinical healthcare for those living in remote and rural areas, vulnerable groups and ageing populations [1; 2]. |
| **Telehealth providers and specialties** |
| Telehealth may be provided by all health professions including nurses, nutritionists, pharmacists, physicians, physiotherapists, psychologists and others [1; 15]. Further, telehealth services comprise a range of specialties such as preventive medicine, physical medicine, physical rehabilitation (including musculoskeletal pain) and others [4; 9]. |
| **Telehealth interventions, platforms and components** |
| Telehealth may be used to provide different treatments such as health education, exercise and other self-management strategies [28]. It can be used to deliver care individually or in groups [14; 29] by synchronous (i.e., interactive) modes such as video conference, asynchronous (i.e., store-forward) modes such as smartphone applications [29] or both, or using remote monitoring strategies. |

| **Supplementary file 1 B. Social media survey information available for interested participants to sign up for the team members contact** |
| --- |
| **Terminology and Research Priorities in Telehealth in Musculoskeletal Pain Research** |
| ***Delphi Study Coordinators:*** Mr Junior Fandim (PhD Student) and A/Prof Bruno Saragiotto  ***Research Team:*** Professor Blake Dear, Professor Rana Hinman, Professor Jan Hartvigsen, Ms Cecilie K. Øverås, Dr Saurab Sharma, Ms Joletta Belton, Professor Vinícius Oliveira, Professor Romy Parker, Professor Babita Ghai, Professor Kim Bennell, Professor Paulo Ferreira.  Thank you for contacting us to participate in our study.  We aim to develop the first consensus on the standardization of terminologies to be used in telehealth and determine research priorities for musculoskeletal pain telehealth practice.  We would like to hear the voice of all stakeholders involved in telehealth in musculoskeletal pain.  ***Who can participate?***  ***Musculoskeletal pain telehealth researcher and thought leaders***  - Researchers listed in the Expertscape website on the topics “Internet-based intervention”, “telerehabilitation”, “telemedicine”, “remote consultation” or who has at least one published clinical trial or systematic review indexed in PubMed in the last 10 years.  - Individuals who have developed or published documents focusing on telehealth and health policy relevant to musculoskeletal health.  ***Clinicians***  - Registered healthcare professionals in their home country with clinical experience treating patients with musculoskeletal pain with an average of 12 patients treated via telehealth in the last 12 months.  ***Consumer representatives***  - Patients or individuals, health service users, caregivers and family members with personal experience of musculoskeletal pain. This also includes consumer representatives or advocates (e.g., pain patient advocates, patient partners, public partners, professional body and others) who have had some experience with telehealth-delivered care or participated in research on telehealth for musculoskeletal pain.  ***Healthcare managers***  - Healthcare managers working in healthcare organizations (e.g., public health units) in a manager’s position (e.g., clinical leaders teams).  ***Industry partners and developers***  - Individuals, team members, organizations or other entities (e.g., industry partners and developers) who work in the development of telehealth solutions related to musculoskeletal pain care.  ***Policymakers***  - Individuals, organizations, and entities (e.g., scientific advisors, civil servants, ministers, or politicians) who make formulation, management, implementation of public or private health policy on public health programs and services at any level of government (e.g., local, state, provincial, national, or international) related to telehealth-delivered care or telehealth research.  The e-Delphi survey is estimated to be completed in three rounds. Each round consists of an online survey that will take approximately ***15-30*** minutes to complete.  If you would like to participate in the e-Delphi survey, please fill in the following data so we can contact you.  **Panel member basic contact information**  We will ask you a few questions and then we will contact you. |
| **Let's start with your first and last name** |
| **What email address can we reach you at?**  (This is only to get in touch, not to send spam). |
| **Which stakeholder do you identify with?**  Please select only one option (choose the option that best represents your main role). |
| A) Clinician  B) Consumer representative  C) Healthcare manager  D) Policymakers  E) Researchers/thought leaders  F) Industry partners and developers |
| **How did you hear about our study?** |
| A) Facebook  B) Twitter  C) Instagram  D) LinkedIn  E) Google or websites  F) Other |
| **Final message** |
| Dear *First and last name*  That's everything.  We would like to thank you for your interest in participating in the e-Delphi survey.  Your voice and experience will be essential.  We'll send you an email with some details after you submit this form. |
|  |

| **Supplementary file 1 C. Typeform survey information and set of questions to potential participants to confirm eligibility and proceed to the sections** |
| --- |
| **Terminology and Research Priorities in Telehealth for Musculoskeletal Pain Research** |
| ***Delphi Study Coordinators:*** Mr Junior Fandim (PhD Student) and A/Prof Bruno Saragiotto  ***Research Team:*** Professor Blake Dear, Professor Rana Hinman, Professor Jan Hartvigsen, Ms Cecilie K. Øverås, Dr Saurab Sharma, Ms Joletta Belton, Professor Vinícius Oliveira, Professor Romy Parker, Professor Babita Ghai, Professor Kim Bennell, Professor Paulo Ferreira.  We aim to develop the first consensus on the standardization of terminologies to be used in telehealth and determine research priorities for musculoskeletal pain telehealth practice.  We would like to hear the voice of all stakeholders involved in telehealth in musculoskeletal pain.  Thank you for agreeing to participate in this study. We believe that research focused on topics relevant to all stakeholders is more likely to be highly beneficial.  ***Who can participate?***  ***Musculoskeletal pain telehealth researchers and thought leaders***  - Researchers listed in the Expertscape website on the topics “Internet-based intervention”, “telerehabilitation”, “telemedicine”, “remote consultation” or who has at least one published clinical trial or systematic review indexed in PubMed in the last 10 years.  - Individuals who have developed or published documents focusing on telehealth and health policy relevant to musculoskeletal health.  ***Clinicians***  - Registered healthcare professionals in their home country with clinical experience treating patients with musculoskeletal pain with an average of 12 patients treated via telehealth in the last 12 months.  ***Consumer representatives***  - Patients or individuals, health service users, caregivers and family members with personal experience of musculoskeletal pain. This also includes consumer representatives or advocates (e.g., pain patient advocates, patient partners, public partners, professional body and others) who have had some experience with telehealth-delivered care or participated in research on telehealth for musculoskeletal pain.  ***Healthcare managers***  - Healthcare managers working in healthcare organizations (e.g., public health units) in a manager’s position (e.g., clinical leaders teams).  ***Industry partners and developers***  - Individuals, team members, organizations or other entities (e.g., industry partners and developers) who work in the development of telehealth solutions related to musculoskeletal pain care.  ***Policymakers***  - Individuals, organizations, and entities (e.g., scientific advisors, civil servants, ministers, or politicians) who make formulation, management, implementation of public or private health policy on public health programs and services at any level of government (e.g., local, state, provincial, national, or international) related to telehealth-delivered care or telehealth research.  This e-Delphi survey is estimated to be completed in three rounds. Each round consists of an online survey that will take approximately ***15-30*** minutes to complete. |
| **e-Delphi survey contents:** |
| Section 1: Participant Information Sheet and background questions  Section 2: Standard terminologies for telehealth in musculoskeletal pain research  Section 3: Research priorities for telehealth in musculoskeletal pain research |
| **Participant Information Sheet and background questions** |
| **Do you accept to participate in this International Modified e-Delphi Study?**  Please see the complete participant information sheet ***here*** |
| A) I accept  B) I do not accept |
| First and last name |
| Please enter your e-mail address |
| **What is your main Stakeholder Group?**  Please select only one option (choose the option that best represents your main role). |
| A) Clinician  B) Consumer representative  C) Healthcare manager  D) Policymakers  E) Researchers/thought leaders  F) Industry partners and developers |
| **Clinicians' background information**  For this section, we will have specific questions for registered healthcare professionals in their home country and clinical experience treating patients with musculoskeletal pain via telehealth. |
| **What's your age?** |
| **What is your gender?** |
| A) Woman  B) Man  C) Prefer not to say  D) I identify as____ (Please specify your gender) |
| **Highest level of education** |
| A) University Bachelor’s Degree  B) University Higher Education Master's Degree  C) University Higher Education PhD Degree  D) Other (please, specify) |
| **Which country do you currently reside in?** |
| - Select an option |
| **Consumer representatives' background information**  For this section, we will have specific questions for consumer representatives defined as any patients or people with personal experience of musculoskeletal pain, health service users, caregivers and family members. This also includes consumer representatives or advocates (e.g., pain patient advocates, patient partners, public partners, professional body and others) who have had some experience with telehealth-delivered care or participated in research on telehealth for musculoskeletal pain. |
| **What's your age?** |
| **What is your gender?** |
| A) Woman  B) Man  C) Prefer not to say  D) I identify as____ (Please specify your gender) |
| **Highest level of education** |
| A) Secondary/High School  B) Diploma/Certificate/Apprenticeship  C) University Bachelor’s Degree  D) University Higher Education Master's Degree  E) University Higher Education PhD Degree  F) University Higher Education Degree (other) |
| **Which country do you currently reside in?** |
| - Select an option |
| **Healthcare managers' background information**  For this section, we will have specific questions for individuals working in healthcare organizations (e.g., public health units) in a manager’s position (e.g., clinical leaders teams). |
| **What's your age?** |
| **What is your gender?** |
| A) Woman  B) Man  C) Prefer not to say  D) I identify as____ (Please specify your gender) |
| **Highest level of education** |
| A) Secondary/High School  B) Diploma/Certificate/Apprenticeship  C) University Bachelor’s Degree  D) University Higher Education Master's Degree  E) University Higher Education Phd's Degree  F) University Higher Education Degree (other) |
| **Which country do you currently reside in?** |
| - Select an option |
| **Policymakers' background information**  For this section, we will have specific questions for individuals, organizations, and entities (e.g., scientific advisors, civil servants, ministers, or politicians) who make formulation, management, implementation of public or private health policy on public health programs and services at any level of government (e.g., local, state, provincial, national, or international) related to telehealth. |
| **What's your age?** |
| **What is your gender?** |
| A) Woman  B) Man  C) Prefer not to say  D) I identify as____ (Please specify your gender) |
| **Highest level of education** |
| A) Secondary/High School  B) Diploma/Certificate/Apprenticeship  C) University Bachelor’s Degree  D) University Higher Education Master's Degree  E) University Higher Education Phd's Degree  F) University Higher Education Degree (other) |
| **Which country do you currently reside in?** |
| - Select an option |
| **Researcher's background information**  For this section, we will have specific questions for telehealth musculoskeletal pain researchers. |
| **What's your age?** |
| **What is your gender?** |
| A) Woman  B) Man  C) Prefer not to say  D) I identify as____ (Please specify your gender) |
| **Highest level of education** |
| A) Secondary/High School  B) Diploma/Certificate/Apprenticeship  C) University Bachelor’s Degree  D) University Higher Education Master's Degree  E) University Higher Education PhD Degree  F) University Higher Education Degree (other) |
| **Which country do you currently reside in?** |
| - Select an option |
| **What is your area of research?** |
| Choose as many as you like  A) Back pain  B) Acute and subacute pain  C) Chronic pain  D) Foot and/or ankle pain  E) Hip and/or knee pain  F) Neck pain  G) Shoulder, upper limb and/or hand pain  H) Thoracic spine pain  I) Other |
| **What is the *main* treatment approach that you research in telehealth?** |
| A) Behavioral or psychological therapies  B) Education  C) Exercise & physical activity  D) Electronic devices development  E) Public/population health  F) Self-management  G) Multi-component (e.g., education and exercise)  H) Other |
| **What telehealth modality(s) have you been researching?** |
| If none, please proceed forward  Choose as many as you like  A) E-mail  B) SMS/Text messages  C) Smartphone application  D) Video conference  E) Website platform  F) Telephone call  G) Other |
| **Industry partner and developer background information**  For this section, we will have specific questions for individuals, team members, organizations or other entities (e.g., industry partners and developers) who work in the development of telehealth solutions related to musculoskeletal pain care. |
| **What's your age?** |
| **What is your gender?** |
| A) Woman  B) Man  C) Prefer not to say  D) I identify as____ (Please specify your gender) |
| **Highest level of education** |
| A) Secondary/High School  B) Diploma/Certificate/Apprenticeship  C) University Bachelor’s Degree  D) University Higher Education Master's Degree  E) University Higher Education Phd's Degree  F) University Higher Education Degree (other) |
| **Which country do you currently reside in?** |
| - Select an option |
| **What is your area of work?** |
| Choose as many as you like  A) Back pain  B) Acute and subacute pain  C) Chronic pain  D) Foot and/or ankle pain  E) Hip and/or knee pain  F) Neck pain  G) Shoulder, upper limb and/or hand pain  H) Thoracic spine pain  I) Other |
| **What is the *main* treatment approach that you work in telehealth?** |
| A) Behavioral or psychological therapies  B) Education  C) Exercise & physical activity  D) Electronic devices development  E) Public/population health  F) Self-management  G) Multi-component (e.g., education and exercise)  H) Other |
| **What telehealth modality(s) have you been working with?**  If none, please proceed forward |
| Choose as many as you like  A) E-mail  B) SMS/Text messages  C) Smartphone application  D) Video conference  E) Website platform  F) Telephone call  G) Other |
| **Automatic message for participants who met the criteria and agreed to participate** |
| Dear *First and last name*  Firstly, we would like to thank you for participating in the first round of the e-Delphi survey.  Your voice, help, and expertise were essential in taking the first step on the consensus for the standardization of terminology to be used in telehealth and determine research priorities for musculoskeletal pain telehealth practice.  We will be in touch soon to conduct the second round. |
| **Automatic message for participants who did not meet the criteria** |
| Apologies.  You ended up not meeting some criteria regarding the consumer representative, clinician, researcher, health manager, policymaker interested or involved in healthcare or telehealth research.  But thank you for your intention to help us take the first step on the standardization of terminology to be used in telehealth and determine research priorities for musculoskeletal pain telehealth practice. |
|  |
