# Supplementary material for: One step at a time. Shaping consensus on research priorities and terminology in telehealth in musculoskeletal pain: an international modified e-Delphi study
Source: BMC Musculoskelet Disord. 2023 Oct 3;24:783. doi: 10.1186/s12891-023-06866-0 (PMC10546725; doi:10.1186/s12891-023-06866-0)
Supplement: Supplementary file 2 — Additional file 2: Supplementary file 2. Electronic search strategy at PubMed. [file 12891_2023_6866_MOESM2_ESM.docx]

| **Supplementary file 2. Electronic search strategy at PubMed** | | | |
| --- | --- | --- | --- |
| **#** | **Search** |  |  |
| **1** | telemedicine [MeSH Terms] |  |  |
| **2** | telemedicine [Title/Abstract] |  |  |
| **3** | telerehabilitation [MeSH Terms] |  |  |
| **4** | "electronic health" [Title/Abstract] |  |  |
| **5** | digital health [Title/Abstract] |  |  |
| **6** | "mobile medicine" [Title/Abstract] |  |  |
| **7** | "smart phone" or "mobile phone" [Title] |  |  |
| **8** | phone [Title/Abstract] |  |  |
| **9** | telephone [Title/Abstract] |  |  |
| **10** | telephone [MeSH Terms] |  |  |
| **11** | apps [Title/Abstract] |  |  |
| **12** | application program* [Title/Abstract] |  |  |
| **13** | application software* [Title/Abstract] |  |  |
| **14** | mobile Applications [MeSH Terms] |  |  |
| **15** | mobile communication* [Title/Abstract] |  |  |
| **16** | "mobile technology" [Title/Abstract] |  |  |
| **17** | SMS [Title/Abstract] |  |  |
| **18** | text messaging [Title/Abstract] |  |  |
| **19** | text Messaging [MeSH Terms] |  |  |
| **20** | internet [MeSH Terms] |  |  |
| **21** | internet [Title/Abstract] |  |  |
| **22** | online [Title/Abstract] |  |  |
| **23** | "computer based" [Title/Abstract] |  |  |
| **24** | wireless.mp |  |  |
| **25** | video conferencing [MeSH Terms] |  |  |
| **26** | "tablet device" [Title/Abstract] |  |  |
| **27** | iPad [Title/Abstract] |  |  |
| **28** | iPhone [Title/Abstract] |  |  |
| **29** | **OR/28** |  |  |
| **30** | musculoskeletal pain[MeSH Terms] |  |  |
| **31** | musculo*[Title/Abstract] OR muscular NEAR pain[Title/Abstract] |  |  |
| **32** | back pain[MeSH Terms] |  |  |
| **33** | neck pain[MeSH Terms] |  |  |
| **34** | Spine[MeSH Terms] |  |  |
| **35** | back[Title/Abstract] |  |  |
| **36** | lumbar[Title/Abstract] |  |  |
| **37** | lumbo*[Title/Abstract] |  |  |
| **38** | spine[Title/Abstract] OR spinal NEAR pain[Title/Abstract] |  |  |
| **39** | neck[Title/Abstract] OR cervical NEAR pain[Title/Abstract] |  |  |
| **40** | knee*[Title/Abstract] OR hip[Title/Abstract] OR hips[Title/Abstract] OR shoulder*[Title/Abstract] OR hand NEAR pain[Title/Abstract] |  |  |
| **41** | chronic Pain[MeSH Terms] |  |  |
| **42** | Arthritis[Title/Abstract] |  |  |
| **43** | Arthritis, Rheumatoid[MeSH Terms] |  |  |
| **44** | osteoarthrit*[Title/Abstract] |  |  |
| **45** | spondylitis[Title/Abstract] |  |  |
| **46** | spondylosis[Title/Abstract] |  |  |
| **47** | osteitis[Title/Abstract] |  |  |
| **48** | osteochondritis[Title/Abstract] |  |  |
| **49** | arthropathy[Title/Abstract] |  |  |
| **50** | neurogenic[Title/Abstract] |  |  |
| **51** | arthropathy[Title/Abstract] OR neurogenic[Title/Abstract] OR bursitis[Title/Abstract] OR shoulder NEXT impingement[Title/Abstract] |  |  |
| **52** | myalgia[Title/Abstract] |  |  |
| **53** | lordosis[Title/Abstract] |  |  |
| **54** | lumbago[Title/Abstract] |  |  |
| **55** | sciatica[Title/Abstract] |  |  |
| **56** | cervicogenic[Title/Abstract] |  |  |
| **57** | dyskinesis[Title/Abstract] |  |  |
| **58** | tendinitis[Title/Abstract] |  |  |
| **59** | joint ADJ pain[Title/Abstract] |  |  |
| **60** | radicular ADJ pain[Title/Abstract] |  |  |
| **61** | allodynia[Title/Abstract] |  |  |
| **62** | hyperalgesia[Title/Abstract] |  |  |
| **63** | sacroiliac[Title/Abstract] |  |  |
| **64** | subluxation[Title/Abstract] |  |  |
| **65** | disc[Title/Abstract] |  |  |
| **66** | misalignment[Title/Abstract] |  |  |
| **67** | osteopathic ADJ lesion[Title/Abstract] |  |  |
| **68** | frozen ADJ shoulder[Title/Abstract] |  |  |
| **69** | degenerative ADJ joint ADJ disease[Title/Abstract] |  |  |
| **70** | whiplash Injuries[MeSH Terms] |  |  |
| **71** | myofascial pain syndromes[MeSH Terms] |  |  |
| **72** | "myofascial pain syndromes"[Title/Abstract] |  |  |
| **73** | fibromyalgia[MeSH Terms] |  |  |
| **74** | OR/73 |  |  |
| **75** | 29 AND 73 |  |  |
| Filters: Randomized Controlled Trial, Systematic Review, in the last 10 years, Humans | |  |  |
