# Supplementary material for: One step at a time. Shaping consensus on research priorities and terminology in telehealth in musculoskeletal pain: an international modified e-Delphi study
Source: BMC Musculoskelet Disord. 2023 Oct 3;24:783. doi: 10.1186/s12891-023-06866-0 (PMC10546725; doi:10.1186/s12891-023-06866-0)
Supplement: Supplementary file 3 — Additional file 3: Supplementary file 3. A. First round panel members' group rating agreement on telehealth research priorities. B. First round panel members' rating ranked from highest to lowest importance on telehealth research priorities ranked from highest to lowest. C. First round panel members' group rating agreement in percent on telehealth research priorities ranked from highest to lowest. D. First round panel members' rate by income-level supporting the use of the term as standard terminology ranked from highest to lowest. [file 12891_2023_6866_MOESM3_ESM.docx]

**Supplementary file 3 A. First round panel members' group rating agreement on telehealth research priorities**

**Supplementary file 3 A. First round panel members' rating agreement in percent on telehealth research priorities ranked from highest to lowest.**

**Supplementary file 3 A. First round panel members' rating agreement in percent on telehealth research priorities ranked from highest to lowest.**

Research Priority Abbreviations: Research about how to implement telehealth services at the user, clinician and health system level; Effectiveness of treatment approaches delivered via telehealth in the management of musculoskeletal, Equity research on interventions to improve access, treatment or clinical outcomes to telehealth services for disadvantaged or historically underserved populations with musculoskeletal conditions; conditions; The cost-effectiveness of telehealth treatments for musculoskeletal conditions; Design and evaluation of curricula to train students and health care practitioners in the provision of telehealth that conforms to core capability frameworks; Research on health literacy, eHealth literacy, technology literacy and identifying relevant factors for patients with musculoskeletal conditions engaging in telehealth (eg, barriers and facilitators);Identification of patient characteristics that affects the response to treatments delivered by telehealth; Investigation of harms and adverse events during telehealth encounters for musculoskeletal conditions; Integration of telehealth devices with electronic health records and cloud databases; Research on suitable outcome measures for telehealth in individuals with musculoskeletal conditions; Translation, dissemination and communication developed with stakeholders; Research that examines the specific contribution of communication and information technology to the effectiveness of telehealth treatments in musculoskeletal conditions;Standardization of telehealth-related terms and the development of frameworks for categorizing different telehealth approaches in musculoskeletal practice; Identification of mediators that explain or contribute to the mechanisms underpinning the effects of telehealth-delivered treatments (e.g. , self-efficacy); Identification of clinician characteristics that affect response; New developments and advances in telehealth communication and information technologies considering predictive models and the use of artificial intelligence; Development of algorithms and analytical approaches for predictive models, personalized, and customized analytics, and devices to improve assessment and management of musculoskeletal conditions; Research on diagnostic tests suitable for telehealth in individuals with musculoskeletal conditions; Identify, explore, and implement the most suitable business models to support the delivery of telehealth treatment for individuals with musculoskeletal conditions; The role of organizations and advisory boards in supporting the use of evidence-based telehealth in musculoskeletal conditions.

**Supplementary file 3 B. First round panel members' rating ranked from highest to lowest importance on telehealth**

**research priorities ranked from highest to lowest.**

**Supplementary file 3 B. First round panel members' rating ranked from highest to lowest importance on telehealth research**

**priorities ranked from highest to lowest.**

Research Priority Abbreviations: Research about how to implement telehealth services at the user, clinician and health system level; Effectiveness of treatment approaches delivered via telehealth in the management of musculoskeletal, Equity research on interventions to improve access, treatment or clinical outcomes to telehealth services for disadvantaged or historically underserved populations with musculoskeletal conditions; conditions; The cost-effectiveness of telehealth treatments for musculoskeletal conditions; Design and evaluation of curricula to train students and health care practitioners in the provision of telehealth that conforms to core capability frameworks; Research on health literacy, eHealth literacy, technology literacy and identifying relevant factors for patients with musculoskeletal conditions engaging in telehealth (eg, barriers and facilitators);Identification of patient characteristics that affects the response to treatments delivered by telehealth; Investigation of harms and adverse events during telehealth encounters for musculoskeletal conditions; Integration of telehealth devices with electronic health records and cloud databases; Research on suitable outcome measures for telehealth in individuals with musculoskeletal conditions; Translation, dissemination and communication developed with stakeholders; Research that examines the specific contribution of communication and information technology to the effectiveness of telehealth treatments in musculoskeletal conditions; Standardization of telehealth-related terms and the development of frameworks for categorizing different telehealth approaches in musculoskeletal practice; Identification of mediators that explain or contribute to the mechanisms underpinning the effects of telehealth-delivered treatments (e.g. , self-efficacy); Identification of clinician characteristics that affect response; New developments and advances in telehealth communication and information technologies considering predictive models and the use of artificial intelligence; Development of algorithms and analytical approaches for predictive models, personalized, and customized analytics, and devices to improve assessment and management of musculoskeletal conditions; Research on diagnostic tests suitable for telehealth in individuals with musculoskeletal conditions; Identify, explore, and implement the most suitable business models to support the delivery of telehealth treatment for individuals with musculoskeletal conditions; The role of organizations and advisory boards in supporting the use of evidence-based telehealth in musculoskeletal conditions.

**Supplementary file 3 C. First round panel members' group rating agreement in percent on telehealth research priorities ranked from highest to lowest.**

**Supplementary file 3 C. First round panel members' group rating agreement in percent on telehealth research priorities ranked from highest to lowest.**

Research Priority Abbreviations: Research about how to implement telehealth services at the user, clinician and health system level; Effectiveness of treatment approaches delivered via telehealth in the management of musculoskeletal, Equity research on interventions to improve access, treatment or clinical outcomes to telehealth services for disadvantaged or historically underserved populations with musculoskeletal conditions; conditions; The cost-effectiveness of telehealth treatments for musculoskeletal conditions; Design and evaluation of curricula to train students and health care practitioners in the provision of telehealth that conforms to core capability frameworks; Research on health literacy, eHealth literacy, technology literacy and identifying relevant factors for patients with musculoskeletal conditions engaging in telehealth (eg, barriers and facilitators);Identification of patient characteristics that affects the response to treatments delivered by telehealth; Investigation of harms and adverse events during telehealth encounters for musculoskeletal conditions; Integration of telehealth devices with electronic health records and cloud databases; Research on suitable outcome measures for telehealth in individuals with musculoskeletal conditions; Translation, dissemination and communication developed with stakeholders; Research that examines the specific contribution of communication and information technology to the effectiveness of telehealth treatments in musculoskeletal conditions;Standardization of telehealth-related terms and the development of frameworks for categorizing different telehealth approaches in musculoskeletal practice; Identification of mediators that explain or contribute to the mechanisms underpinning the effects of telehealth-delivered treatments (e.g. , self-efficacy); Identification of clinician characteristics that affect response; New developments and advances in telehealth communication and information technologies considering predictive models and the use of artificial intelligence; Development of algorithms and analytical approaches for predictive models, personalized, and customized analytics, and devices to improve assessment and management of musculoskeletal conditions; Research on diagnostic tests suitable for telehealth in individuals with musculoskeletal conditions; Identify, explore, and implement the most suitable business models to support the delivery of telehealth treatment for individuals with musculoskeletal conditions; The role of organizations and advisory boards in supporting the use of evidence-based telehealth in musculoskeletal conditions.

**Supplementary file 3 D. First round panel members' rate by income-level supporting the use of the term as standard terminology ranked from highest to lowest.**

**Supplementary file 3 D. First round panel members' rate by income-level supporting the use of the term as standard terminology ranked from highest to lowest.**

Research Priority Abbreviations: Research about how to implement telehealth services at the user, clinician and health system level; Effectiveness of treatment approaches delivered via telehealth in the management of musculoskeletal, Equity research on interventions to improve access, treatment or clinical outcomes to telehealth services for disadvantaged or historically underserved populations with musculoskeletal conditions; conditions; The cost-effectiveness of telehealth treatments for musculoskeletal conditions; Design and evaluation of curricula to train students and health care practitioners in the provision of telehealth that conforms to core capability frameworks; Research on health literacy, eHealth literacy, technology literacy and identifying relevant factors for patients with musculoskeletal conditions engaging in telehealth (eg, barriers and facilitators);Identification of patient characteristics that affects the response to treatments delivered by telehealth; Investigation of harms and adverse events during telehealth encounters for musculoskeletal conditions; Integration of telehealth devices with electronic health records and cloud databases; Research on suitable outcome measures for telehealth in individuals with musculoskeletal conditions; Translation, dissemination and communication developed with stakeholders; Research that examines the specific contribution of communication and information technology to the effectiveness of telehealth treatments in musculoskeletal conditions;Standardization of telehealth-related terms and the development of frameworks for categorizing different telehealth approaches in musculoskeletal practice; Identification of mediators that explain or contribute to the mechanisms underpinning the effects of telehealth-delivered treatments (e.g. , self-efficacy); Identification of clinician characteristics that affect response; New developments and advances in telehealth communication and information technologies considering predictive models and the use of artificial intelligence; Development of algorithms and analytical approaches for predictive models, personalized, and customized analytics, and devices to improve assessment and management of musculoskeletal conditions; Research on diagnostic tests suitable for telehealth in individuals with musculoskeletal conditions; Identify, explore, and implement the most suitable business models to support the delivery of telehealth treatment for individuals with musculoskeletal conditions; The role of organizations and advisory boards in supporting the use of evidence-based telehealth in musculoskeletal conditions.
