# Supplementary material for: One step at a time. Shaping consensus on research priorities and terminology in telehealth in musculoskeletal pain: an international modified e-Delphi study
Source: BMC Musculoskelet Disord. 2023 Oct 3;24:783. doi: 10.1186/s12891-023-06866-0 (PMC10546725; doi:10.1186/s12891-023-06866-0)
Supplement: Supplementary file 4 — Additional file 4: Supplementary file 4. A. Second round panel members' group rating agreement on telehealth research priorities. B. Second round panel members' rating ranked from highest to lowest importance on telehealth research priorities ranked from highest to lowest. C. Second round panel members' group rating agreement in percent on telehealth research priorities ranked from highest to lowest. D. Second round panel members' rate by income-level supporting the use of the term as standard terminology ranked from highest to lowest. [file 12891_2023_6866_MOESM4_ESM.docx]

**Supplementary file 4. Second round panel members' group rating agreement on telehealth research priorities**

**Supplementary file 4 A. Second round panel members' rating agreement in percent on telehealth research priorities ranked from highest to lowest.**

**Supplementary file 4 A. Second round panel members' group rating agreement in percent on telehealth research priorities ranked from highest to lowest.**

Research Priority Abbreviations: Identification of patient characteristics that affect response to treatments delivered by telehealth; Qualitative telehealth research to determine perceptions, barriers, and enablers in the management of musculoskeletal conditions; Research that examines the specific contribution of communication and information technology and digital skills to the effectiveness of telehealth treatments in musculoskeletal conditions; Standardization of telehealth-related terms and the development of frameworks and guidelines for musculoskeletal telehealth practice; Investigation of patient-related safety risks and adverse events during telehealth encounters for musculoskeletal conditions; Research on reliability and validity testing for diagnostic tests suitable for telehealth (compared to in-person testing) in individuals with musculoskeletal conditions; Integration of telehealth devices with electronic health records and cloud databases; Identification of mediators contributing to the effects of telehealth-delivered treatments; Research on suitable patient-oriented research outcome measures for telehealth in individuals with musculoskeletal conditions; Development of algorithms and analytical approaches for predictive models, personalized, and customized analytics, and devices to improve assessment and management of musculoskeletal conditions; Translation, dissemination and communication developed with all parties involved; The role of organizations and advisory boards in supporting the use of evidence-based telehealth in musculoskeletal conditions; New developments and advances in telehealth communication and information technologies considering predictive models and the use of artificial intelligence; Identify, explore, and implement the most suitable business models to support the delivery of telehealth treatment for individuals with musculoskeletal conditions; Identification of clinician characteristics and beliefs that affect response.

**Supplementary file 4 B. Second round panel members' rating ranked from highest to lowest importance on telehealth**

**research priorities ranked from highest to lowest.**

**Supplementary file 4 C. Second round panel members' group rating agreement in percent on telehealth research priorities ranked from highest to lowest.**

**Supplementary file 4 C. Second round panel members' group rating agreement in percent on telehealth research priorities ranked from highest to lowest.**

Research Priority Abbreviations: Identification of patient characteristics that affect response to treatments delivered by telehealth; Qualitative telehealth research to determine perceptions, barriers, and enablers in the management of musculoskeletal conditions; Research that examines the specific contribution of communication and information technology and digital skills to the effectiveness of telehealth treatments in musculoskeletal conditions; Standardization of telehealth-related terms and the development of frameworks and guidelines for musculoskeletal telehealth practice; Investigation of patient-related safety risks and adverse events during telehealth encounters for musculoskeletal conditions; Research on reliability and validity testing for diagnostic tests suitable for telehealth (compared to in-person testing) in individuals with musculoskeletal conditions; Integration of telehealth devices with electronic health records and cloud databases; Identification of mediators contributing to the effects of telehealth-delivered treatments; Research on suitable patient-oriented research outcome measures for telehealth in individuals with musculoskeletal conditions; Development of algorithms and analytical approaches for predictive models, personalized, and customized analytics, and devices to improve assessment and management of musculoskeletal conditions; Translation, dissemination and communication developed with all parties involved; The role of organizations and advisory boards in supporting the use of evidence-based telehealth in musculoskeletal conditions; New developments and advances in telehealth communication and information technologies considering predictive models and the use of artificial intelligence; Identify, explore, and implement the most suitable business models to support the delivery of telehealth treatment for individuals with musculoskeletal conditions; Identification of clinician characteristics and beliefs that affect response.

**Supplementary file 4 D. Second round panel members' rate by income-level supporting the use of the term as standard terminology ranked from highest to lowest.**

**Supplementary file 4 D. Second round panel members' rate by income-level supporting the use of the term as standard terminology ranked from highest to lowest.**

Research Priority Abbreviations: Identification of patient characteristics that affect response to treatments delivered by telehealth; Qualitative telehealth research to determine perceptions, barriers, and enablers in the management of musculoskeletal conditions; Research that examines the specific contribution of communication and information technology and digital skills to the effectiveness of telehealth treatments in musculoskeletal conditions; Standardization of telehealth-related terms and the development of frameworks and guidelines for musculoskeletal telehealth practice; Investigation of patient-related safety risks and adverse events during telehealth encounters for musculoskeletal conditions; Research on reliability and validity testing for diagnostic tests suitable for telehealth (compared to in-person testing) in individuals with musculoskeletal conditions; Integration of telehealth devices with electronic health records and cloud databases; Identification of mediators contributing to the effects of telehealth-delivered treatments; Research on suitable patient-oriented research outcome measures for telehealth in individuals with musculoskeletal conditions; Development of algorithms and analytical approaches for predictive models, personalized, and customized analytics, and devices to improve assessment and management of musculoskeletal conditions; Translation, dissemination and communication developed with all parties involved; The role of organizations and advisory boards in supporting the use of evidence-based telehealth in musculoskeletal conditions; New developments and advances in telehealth communication and information technologies considering predictive models and the use of artificial intelligence; Identify, explore, and implement the most suitable business models to support the delivery of telehealth treatment for individuals with musculoskeletal conditions; Identification of clinician characteristics and beliefs that affect response.
