# Supplementary material for: One step at a time. Shaping consensus on research priorities and terminology in telehealth in musculoskeletal pain: an international modified e-Delphi study
Source: BMC Musculoskelet Disord. 2023 Oct 3;24:783. doi: 10.1186/s12891-023-06866-0 (PMC10546725; doi:10.1186/s12891-023-06866-0)
Supplement: Supplementary file 5 — Additional file 5: Supplementary file 5. A. Third round panel members' rating agreement in percent on telehealth research priorities ranked from highest to lowest. B. Third round panel members' rating ranked from highest to lowest importance on telehealth research priorities ranked from highest to lowest. C. Third round panel members' group rating agreement in percent on telehealth research priorities ranked from highest to lowest. D. Third round panel members' rate by income-level supporting the use of the term as standard terminology ranked from highest to lowest. [file 12891_2023_6866_MOESM5_ESM.docx]

**Supplementary file 5. Third round panel members' group rating agreement on telehealth research priorities**

**Supplementary file 5 A. Third round panel members' rating agreement in percent on telehealth research priorities ranked from highest to lowest.**

**Supplementary file 5 A. Third round panel members' rating agreement in percent on telehealth research priorities ranked from highest to lowest.**

Research Priority Abbreviations: Research and development of strategies for using information and communication technology to facilitate access to individuals with musculoskeletal conditions in remote or rural regions; Research on reliability and validity of clinical assessment and diagnostic tests administered via telehealth (compared to in-person testing) in individuals with musculoskeletal conditions; Identification of mediators contributing to the effects of telehealth-delivered treatments; Investigation of adverse events and patient safety during telehealth encounters for musculoskeletal conditions; Research on suitable patient-oriented research outcome measures for telehealth in individuals with musculoskeletal conditions; Translation, dissemination and communication developed with all parties involved; New developments and advances in telehealth communication and information technologies considering predictive models and the use of artificial intelligence; Data science initiative to support the use of telehealth in musculoskeletal conditions; The role of organizations and advisory boards in supporting the use of evidence-based telehealth in musculoskeletal conditions; Identification of clinician (health professional) characteristics and beliefs that affect response to management via telehealth; Development and testing of innovative business models to support the delivery of telehealth in musculoskeletal conditions.

**Supplementary file 5 B. Third round panel members' rating ranked from highest to lowest importance on telehealth**

**research priorities ranked from highest to lowest.**

**Supplementary file 5 B. Third round panel members' rating ranked from highest to lowest importance on telehealth**

**research priorities ranked from highest to lowest.**

Research Priority Abbreviations: Research and development of strategies for using information and communication technology to facilitate access to individuals with musculoskeletal conditions in remote or rural regions; Research on reliability and validity of clinical assessment and diagnostic tests administered via telehealth (compared to in-person testing) in individuals with musculoskeletal conditions; Identification of mediators contributing to the effects of telehealth-delivered treatments; Investigation of adverse events and patient safety during telehealth encounters for musculoskeletal conditions; Research on suitable patient-oriented research outcome measures for telehealth in individuals with musculoskeletal conditions; Translation, dissemination and communication developed with all parties involved; New developments and advances in telehealth communication and information technologies considering predictive models and the use of artificial intelligence; Data science initiative to support the use of telehealth in musculoskeletal conditions; The role of organizations and advisory boards in supporting the use of evidence-based telehealth in musculoskeletal conditions; Identification of clinician (health professional) characteristics and beliefs that affect response to management via telehealth; Development and testing of innovative business models to support the delivery of telehealth in musculoskeletal conditions.

**Supplementary file 5 C. Third round panel members' group rating agreement in percent on telehealth research priorities ranked from highest to lowest.**

**Supplementary file 5 C. Third round panel members' group rating agreement in percent on telehealth research priorities ranked from highest to lowest.**

Research Priority Abbreviations: Research and development of strategies for using information and communication technology to facilitate access to individuals with musculoskeletal conditions in remote or rural regions; Research on reliability and validity of clinical assessment and diagnostic tests administered via telehealth (compared to in-person testing) in individuals with musculoskeletal conditions; Identification of mediators contributing to the effects of telehealth-delivered treatments; Investigation of adverse events and patient safety during telehealth encounters for musculoskeletal conditions; Research on suitable patient-oriented research outcome measures for telehealth in individuals with musculoskeletal conditions; Translation, dissemination and communication developed with all parties involved; New developments and advances in telehealth communication and information technologies considering predictive models and the use of artificial intelligence; Data science initiative to support the use of telehealth in musculoskeletal conditions; The role of organizations and advisory boards in supporting the use of evidence-based telehealth in musculoskeletal conditions; Identification of clinician (health professional) characteristics and beliefs that affect response to management via telehealth; Development and testing of innovative business models to support the delivery of telehealth in musculoskeletal conditions.

**Supplementary file 5 D. Third round panel members' rate by income-level supporting the use of the term as standard terminology ranked from highest to lowest.**

**Supplementary file 5 D. Third round panel members' rate by income-level supporting the use of the term as standard terminology ranked from highest to lowest.**

Research Priority Abbreviations: Research and development of strategies for using information and communication technology to facilitate access to individuals with musculoskeletal conditions in remote or rural regions; Research on reliability and validity of clinical assessment and diagnostic tests administered via telehealth (compared to in-person testing) in individuals with musculoskeletal conditions; Identification of mediators contributing to the effects of telehealth-delivered treatments; Investigation of adverse events and patient safety during telehealth encounters for musculoskeletal conditions; Research on suitable patient-oriented research outcome measures for telehealth in individuals with musculoskeletal conditions; Translation, dissemination and communication developed with all parties involved; New developments and advances in telehealth communication and information technologies considering predictive models and the use of artificial intelligence; Data science initiative to support the use of telehealth in musculoskeletal conditions; The role of organizations and advisory boards in supporting the use of evidence-based telehealth in musculoskeletal conditions; Identification of clinician (health professional) characteristics and beliefs that affect response to management via telehealth; Development and testing of innovative business models to support the delivery of telehealth in musculoskeletal conditions.
