# Supplementary material for: One step at a time. Shaping consensus on research priorities and terminology in telehealth in musculoskeletal pain: an international modified e-Delphi study
Source: BMC Musculoskelet Disord. 2023 Oct 3;24:783. doi: 10.1186/s12891-023-06866-0 (PMC10546725; doi:10.1186/s12891-023-06866-0)
Supplement: Supplementary file 6 — Additional file 6: Supplementary file 6. A. Final list of panel members' rating agreement level in percent on telehealth research priorities ranked from highest to lowest. B. Final list of panel members' rating group rating agreement level in percent on telehealth research priorities ranked from highest to lowest. C. Final list of panel members' rate by income-level supporting the use of the term as standard terminology ranked from highest to lowest. [file 12891_2023_6866_MOESM6_ESM.docx]

**Supplementary file 6. Final list of panel members' group rating agreement on telehealth research priorities**

**Supplementary file 6 A. Final list of panel members' rating agreement level in percent on telehealth research priorities ranked from highest to lowest.**

**Supplementary file 6 B. Final list of panel members' rating group rating agreement level in percent on telehealth research priorities ranked from highest to lowest.**

**Supplementary file 6 C. Final list of panel members' rate by income-level supporting the use of the term as standard terminology ranked from highest to lowest.**
