# Supplementary material for: One step at a time. Shaping consensus on research priorities and terminology in telehealth in musculoskeletal pain: an international modified e-Delphi study
Source: BMC Musculoskelet Disord. 2023 Oct 3;24:783. doi: 10.1186/s12891-023-06866-0 (PMC10546725; doi:10.1186/s12891-023-06866-0)
Supplement: Supplementary file 7 — Additional file 7: Supplementary file 7. A. Panel members' rating of how familiar they are with the term. B. First round panel members' rate agreement in percent on supporting the use of the term as standard terminology ranked from highest to lowest. C. First round panel members' group rate supporting the use of the term as standard terminology ranked from highest to lowest. D. First round panel members' rate by income-level supporting the use of the term as standard terminology ranked from highest to lowest. [file 12891_2023_6866_MOESM7_ESM.docx]

**Supplementary file 7. First round panel members' group rate supporting the use of the term as**

**standard terminology**

**Supplementary file 7 A. Panel members' rating of how familiar they are with the term.**

**Supplementary file 7 B. First round panel members' rate agreement in percent on supporting the use of the term as standard terminology ranked from highest to lowest.**

**Supplementary file 7 C. First round panel members' group rate supporting the use of the term as standard terminology ranked from highest to lowest.**

**Supplementary file 7 D. First round panel members' rate by income-level supporting the use of the term as standard terminology ranked from highest to lowest**
