# Supplementary material for: One step at a time. Shaping consensus on research priorities and terminology in telehealth in musculoskeletal pain: an international modified e-Delphi study
Source: BMC Musculoskelet Disord. 2023 Oct 3;24:783. doi: 10.1186/s12891-023-06866-0 (PMC10546725; doi:10.1186/s12891-023-06866-0)
Supplement: Supplementary file 9 — Additional file 9: Supplementary file 9. A. Third round panel members' rate agreement in percent on supporting the use of the term as standard terminology ranked from highest to lowest. B. Third round panel members' group rate supporting the use of the term as standard terminology ranked from highest to lowest. C. Third round panel members' rate by income-level supporting the use of the term as standard terminology ranked from highest to lowest. [file 12891_2023_6866_MOESM9_ESM.docx]

**Supplementary file 9. Third round panel members' group rate supporting the use of the term as**

**standard terminology**

**Supplementary file 9 A. Third round panel members' rate agreement in percent on supporting the use of the term as standard terminology ranked from highest to lowest.**

**Supplementary file 9 B. Third round panel members' group rate supporting the use of the term as standard terminology ranked from highest to lowest.**

**Supplementary file 9 C. Third round panel members' rate by income-level supporting the use of the term as standard terminology ranked from highest to lowest.**
