# Supplementary material for: One step at a time. Shaping consensus on research priorities and terminology in telehealth in musculoskeletal pain: an international modified e-Delphi study
Source: BMC Musculoskelet Disord. 2023 Oct 3;24:783. doi: 10.1186/s12891-023-06866-0 (PMC10546725; doi:10.1186/s12891-023-06866-0)
Supplement: Supplementary file 10 — Additional file 10: Supplementary file 10. Feedback with all the raw results of the first round. [file 12891_2023_6866_MOESM10_ESM.docx]

**Supplementary file 10. Feedback with all the raw results of the first round.**

**Which country do you currently reside in?**

**What is your main Stakeholder Group?**

**What is your gender?**

**Highest level of education**

**Panel members (Researchers)**

**What is your area of research?**

**What is the main treatment approach that you research in telehealth?**

**What telehealth modality(s) have you been researching?**

**Panel members (Consumer representative)**

**Which of the musculoskeletal conditions did you receive telehealth for?**

**Which modality(s) of telehealth have you experienced?**

**Panel members (Developer and industry partner)**

**What is your area of work?**

**What is the main treatment approach that you work in telehealth?**

**What telehealth modality(s) have you been working with?**

**Standard terminologies for telehealth in musculoskeletal pain research**

**How familiar you are with the term___?**

**Standard terminologies for telehealth in musculoskeletal pain research**

**Do you support the use of the term ___ as standard terminolgy?**

**Standard terminologies for telehealth in musculoskeletal pain research**

**Do you support the use of the term ___ as standard terminolgy?**

**Standard terminologies for telehealth in musculoskeletal pain research**

**Do you support the use of the term ___ as standard terminolgy?**

**Standard terminologies for telehealth in musculoskeletal pain research**

**Do you support the use of the term ___ as standard terminolgy?**

| Overall summary of the round 1 for the support of the use of the term as standard terminology in telehealth in musculoskeletal pain suggested by the panel members. | | |
| --- | --- | --- |
| Nº | Telehealth terms | Round 1 |
|  |  | Strong agreement (> 80 %) |
| - | - | - |
|  |  | Moderate agreement (70% - 80%) |
| 1 | Telehealth | 77.50% |
| 2 | Mobile health app (mHealth) | 76.25% |
| 3 | Digital health | 73.13% |
| 4 | Telerehabilitation | 70.63% |
|  |  | Low agreement (50% - 70%) |
| 1 | Telemedicine | 69.38% |
| 2 | eHealth Intervention | 69.38% |
| 3 | Video consultations | 65.00% |
| 4 | Electronic health (eHealth) | 63.13% |
| 5 | Self Monitoring | 61.25% |
| 6 | Videoconferencing | 61.25% |
| 7 | Digital health intervention | 60.63% |
| 8 | Teleconsultation | 56.88% |
| 9 | Remote Monitoring | 51.88% |
| 10 | Telephone intervention | 50.63% |
| 11 | Telephysiotherapy | 50.00% |
|  |  | No agreement (< 50%) |
| 1 | Telemonitoring | 48.75% |
| 2 | Internet- and Mobile-Based Intervention | 46.88% |
| 3 | Remote Patient Management (RPM) | 46.88% |
| 4 | Virtual care | 46.88% |
| 5 | Internet-based exercises | 46.25% |
| 6 | Tele-Intervention | 44.38% |
| 7 | Remote treatment or therapy | 43.75% |
| 8 | Telecare | 43.13% |
| 9 | e-consults | 41.88% |
| 10 | Home Telehealth | 40.63% |
| 11 | Tele-assistance | 39.38% |
| 12 | Virtual support | 39.38% |
| 13 | Smartphone-Based Remote Self-Management | 39.38% |
| 14 | Digital physical therapy practice | 37.50% |
| 15 | Personal videoconferencing | 37.50% |
| 16 | Therapist–guided synchronous (ie. live and interactive) telehealth exercise program | 37.50% |
| 17 | Video visits | 36.88% |
| 18 | Remote or non–face-to-face care models | 35.63% |
| 19 | Telecoaching | 33.75% |
| 20 | Telementoring | 33.75% |
| 21 | Digital therapeutics (DTx) | 31.25% |
| 22 | e-visits | 26.88% |
| **New or suggested telehealth terms for round 2** | | |
| 1 | Asynchronous care | - |
| 2 | Audio-simultaneous rehabilitation | - |
| 3 | Blended care | - |
| 4 | Digital care program | - |
| 5 | Digital Health Application (DiHA) | - |
| 6 | Digital rehabilitation | - |
| 7 | Digital Tool | - |
| 8 | Distance physiotherapy | - |
| 9 | Exercise physiology | - |
| 10 | In-person Assessments and Treatment | - |
| 11 | New technology | - |
| 12 | Online care | - |
| 13 | Online consultations | - |
| 14 | Online healthcare | - |
| 15 | Online pain management | - |
| 16 | Pain coaching | - |
| 17 | Pain coach | - |
| 18 | Remote presence | - |
| 19 | Remote self-management support | - |
| 20 | Remotely Delivered Treatment | - |
| 21 | Remote home-based exercise program | - |
| 22 | Remote self-management support | - |
| 23 | Technology-based intervention | - |
| 24 | Tele-assessment | - |
| 25 | Telephone Assessments and Treatment | - |
| 26 | Telepractice | - |
| 27 | Video Assessments and Treatment | - |
| 28 | Virtual appointment | - |
| 29 | Virtual consultations |  |
| 30 | Virtual clinic |  |
| 31 | Virtual exercise prescription |  |
| 33 | Virtual physical assessment and provisional tele diagonosis |  |
| 33 | VR. AR & XR |  |
| 34 | Virtual Physiotherapy |  |
| 35 | Virtual reality asynchronous/synchronous |  |
| 36 | Virtual reality immersive/ non immersive |  |
| **Telehealth terms suggested and repeated with the previous list** | | |
|  | Digital physiotherapy |  |
|  | Digital Physical Therapy |  |
|  | Digital healthcare |  |
|  | mHealth (mobile Health) |  |
|  | mHealth app based rehabilitation |  |
|  | Telephysiotherapy |  |
|  | Telerehabilitation |  |
|  | Telehealth consult |  |
|  | Telehealth consultation |  |
|  | Videoconference |  |
| **Telehealth terms re-edited or modified** | | |
| - | - | - |
| - | - | - |
| **Telehealth terms included in Round 2** | | |
| 1 | Telehealth | 77.50% |
| 2 | Mobile health app (mHealth) | 76.25% |
| 3 | Digital health | 73.13% |
| 4 | Telerehabilitation | 70.63% |
| 5 | Telemedicine | 69.38% |
| 6 | eHealth Intervention | 69.38% |
| 7 | Video consultations | 65.00% |
| 8 | Electronic health (eHealth) | 63.13% |
| 9 | Self Monitoring | 61.25% |
| 10 | Videoconferencing | 61.25% |
| 11 | Digital health intervention | 60.63% |
| 12 | Teleconsultation | 56.88% |
| 13 | Remote Monitoring | 51.88% |
| 14 | Telephone intervention | 50.63% |
| 15 | Telephysiotherapy | 50.00% |
| 16 | Audio-simultaneous rehabilitation | - |
| 17 | Blended care | - |
| 18 | Digital care program | - |
| 19 | Digital Health Application (DiHA) | - |
| 20 | Digital rehabilitation | - |
| 21 | Digital Tool | - |
| 22 | Distance physiotherapy | - |
| 23 | Online consultations | - |
| 24 | Online healthcare | - |
| 25 | Online pain management | - |
| 26 | Remote presence | - |
| 27 | Remote self-management support | - |
| 28 | Remotely Delivered Treatment | - |
| 29 | Remote home-based exercise program | - |
| 30 | Tele-assessment | - |
| 31 | Technology-based intervention | - |
| 32 | Telephone Assessments and Treatment | - |
| 33 | Telepractice | - |
| 34 | Video Assessments and Treatment | - |
| 35 | Virtual appointment | - |
| 36 | Virtual consultations | - |
| 37 | Virtual clinic | - |
| 38 | Virtual exercise prescription | - |
| 39 | Virtual physical assessment and provisional tele diagonosis | - |
| 40 | Virtual reality. Augmented reality & Extended reality (VR, AR & XR) | - |
| 41 | Virtual Physiotherapy | - |
| 42 | Virtual reality asynchronous/synchronous | - |
| 43 | Virtual reality immersive/ non immersive | - |

**Overall suggestions, re-wording, and comments for any terminology provided by the panel members.**

"remote presence"

"none"

"New technology"

"Digital rehabilitation"

"Telepractice"

"remote home-based exercise program"

"virtual reality asynchronous/synchronous"

"virtual reality immersive/ non immersive"

"no"

"Audio-simultaneous rehabilitation"

"Remotely Delivered Treatment"

"Virtual physical assessment and provisional tele diagonosis"

"No"

"Tele-assessment"

"online consultations"

"no"

"Blended care"

"Web based care"

"Online care"

"VR, AR & XR"

"no"

"no"

"Digital Tool"

"technology-based intervention"

"Virtual exercise prescription"

"NA"

"No"

"No"

"mHealth (mobile Health)"

"telehealth consult, telehealth consultation, videoconference"

"no"

"digital physiotherapy, distance physiotherapy"

"RPM often refers to Remote Patient Monitoring instead of Remote Patient Management"

"online healthcare"

"Digital Health Application (DiHA) as used in the German reimbursement system and alternative for DTx."

"Digital care program"

"Asynchronous care"

"no"

"Digital Physical Therapy"

"No, That was very goo"

"Virtual clinic"

"no"

"No"

"No"

"No"

"'Remote self-management support' (and variations on 'remote' to precede self-management"

supported provided virtually)"

"Digital healthcare"

"Online pain management"

"pain coaching"

"no"

"Não"

"pain coaching"

"No"

"No"

"No"

"Telephysiotherapy"

"No everything was fine"

"No"

"Do away with the prefeix digital and e (digi health and e health for example) "

"Exercise physiology"

"Those are applicable during pandemic or in remote places but not recommended as such. "

"No"

"No"

"No"

"telerehabilitation"

"These are sufficient"

"não"

"NA"

"No"

"mHealth app based rehabilitation"

"Virtual physical assessment and provisional tele diagonosis"

"No"

"Video Assessments and Treatment"

"Telephone Assessments and Treatment"

"In-person Assessments and Treatment"

"It may be worth looking at virtual synchronous (live) versus virtual asynchronous"

"Virtual appointment"

"Asynchronous Care"

"Telephysio"

"Virtual consultations"

"Virtual Physiotherapy"

“No"

"No,That was very good"

“No"

"None"

"Remote self-management support' (and variations on 'remote' to precede self-management supported provided virtually)"

"I would not recommend terms that are intervention specific (eg exercise), discipline specific (eg physiotherapy) or suggest unilateral or narrow approach to care (eg self management or monitoring) ""I would not recommend terms that are intervention specific (eg exercise), discipline specific (eg physiotherapy) or suggest unilateral or narrow approach to care (eg self management or monitoring) "

**Research priorities for telehealth in musculoskeletal pain research**

**How much do you agree this should be a research priority?**

**Research priorities for telehealth in musculoskeletal pain research**

**How much do you agree this should be a research priority?**

**Research priorities for telehealth in musculoskeletal pain research**

**How important is this research priority?**

**Research priorities for telehealth in musculoskeletal pain research**

**How much do you agree this should be a research priority?**

**Research priorities for telehealth in musculoskeletal pain research**

**How much do you agree this should be a research priority?**

**Research priorities for telehealth in musculoskeletal pain research**

**How important is this research priority?**

**Research priorities for telehealth in musculoskeletal pain research**

**How important is this research priority?**

| Overall summary of the round 1 for the support of the telehealth research priorities in musculoskeletal pain suggested by the panel members. | | | |
| --- | --- | --- | --- |
| Nº | Telehealth research priorities in the round 1 | Round 1 | |
|  |  | Strong agreement  (>80%) | Level of importance (mean) |
| 1 | Research about how to implement telehealth services at the user, clinician and health system level | 91.88% | 8.4 |
| 2 | Effectiveness of treatment approaches delivered via telehealth in the management of musculoskeletal conditions | 89.38% | 8.5 |
| 3 | The cost-effectiveness of telehealth treatments for musculoskeletal conditions | 88.13% | 8 |
| 4 | Equity research on interventions to improve access. treatment or clinical outcomes to telehealth services for disadvantaged or historically underserved populations with musculoskeletal conditions | 88.13% | 8.3 |
| 5 | Design and evaluation of curricula to train students and health care practitioners in the provision of telehealth that conforms to core capability frameworks | 83.75% | 7.7 |
| 6 | Research on health literacy, eHealth literacy, technology literacy and identifying relevant factors for patients with musculoskeletal conditions engaging in telehealth (eg, barriers and facilitators) | 81.88% | 7.8 |
|  |  | Moderate agreement  (70% - 80%) | Level of importance (mean) |
| 1 | Identification of patient characteristics that affects the response to treatments delivered by telehealth | 80.00% | 7.5 |
| 2 | Investigation of harms and adverse events during telehealth encounters for musculoskeletal conditions | 78.13% | 7.6 |
| 3 | Integration of telehealth devices with electronic health records and cloud databases | 77.50% | 7.4 |
| 4 | Research on suitable outcome measures for telehealth in individuals with musculoskeletal conditions | 76.25% | 7.2 |
| 5 | Translation, dissemination and communication developed with stakeholders | 75.00% | 7.2 |
| 6 | Research that examines the specific contribution of communication and information technology to the effectiveness of telehealth treatments in musculoskeletal conditions | 73.13% | 7.1 |
| 7 | Identification of clinician characteristics that affect response | 72.50% | 6.9 |
| 8 | Identification of mediators that explain or contribute to the mechanisms underpinning the effects of telehealth-delivered treatments (e.g.. self-efficacy) | 72,50% | 7.2 |
| 9 | Standardization of telehealth-related terms and the development of frameworks for categorizing different telehealth approaches in musculoskeletal practice | 72.50% | 7.4 |
| 10 | New developments and advances in telehealth communication and information technologies considering predictive models and the use of artificial intelligence | 71.88% | 7.1 |
|  |  | Low agreement  (50% - 70%) | Level of importance (mean) |
| 1 | Development of algorithms and analytical approaches for predictive models, personalized, and customized analytics, and devices to improve assessment and management of musculoskeletal conditions | 67.50% | 6.8 |
| 2 | Research on diagnostic tests suitable for telehealth in individuals with musculoskeletal conditions | 66.25% | 7.1 |
| 3 | Identify, explore, and implement the most suitable business models to support the delivery of telehealth treatment for individuals with musculoskeletal conditions | 64.38% | 6.8 |
| 4 | The role of organizations and advisory boards in supporting the use of evidence-based telehealth in musculoskeletal conditions | 61.88% | 6.7 |
| **Research priorities included for round 2** | | | |
| 1 | Identification of patient characteristics that affect response to treatments delivered by telehealth | 80.00% | 7.5 |
| 2 | *Investigation of* ***patient-related safety risks and adverse events*** *during telehealth encounters for musculoskeletal conditions* | 78.13% | 7.6 |
| 3 | Integration of telehealth devices with electronic health records and cloud databases | 77.50% | 7.4 |
| 4 | *Research on suitable* ***patient-oriented research*** *outcome measures for telehealth in individuals with musculoskeletal conditions* | 76.25% | 7.2 |
| 5 | *Translation, dissemination and communication developed with* ***all parties involved*** | 75.00% | 7.2 |
| 6 | *Research that examines the specific contribution of communication and information technology and* ***digital skills*** *to the effectiveness of telehealth treatments in musculoskeletal conditions* | 73.13% | 7.1 |
| 7 | *Identification of clinician characteristics* ***and beliefs*** *that affect response* | 72.50% | 6.9 |
| 8 | ***Identification of mediators contributing to the effects of telehealth-delivered treatments*** | 72.50% | 7.2 |
| 9 | *Standardization of telehealth-related terms and the development of frameworks* ***and*** ***guidelines for musculoskeletal telehealth practice*** | 72.50% | 7.4 |
| 10 | New developments and advances in telehealth communication and information technologies considering predictive models and the use of artificial intelligence | 71.88% | 7.1 |
| 11 | Development of algorithms and analytical approaches for predictive models, personalized, and customized analytics, and devices to improve assessment and management of musculoskeletal conditions | 67.50% | 6.8 |
| 12 | ***Research on reliability and validity testing for diagnostic tests suitable for telehealth (compared to in-person testing) in individuals with musculoskeletal conditions*** | 66.25% | 7.1 |
| 13 | Identify, explore, and implement the most suitable business models to support the delivery of telehealth treatment for individuals with musculoskeletal conditions | 64.38% | 6.8 |
| 14 | The role of organizations and advisory boards in supporting the use of evidence-based telehealth in musculoskeletal conditions | 61.88% | 6.7 |
| **New or suggested research priorities for round 2** | | | |
| 1 | Qualitative telehealth research to determine perceptions, barriers, and enablers in the management of musculoskeletal conditions  (*Qualitative research in telehealth to explore and determine in depth the perspectives, facilitators, and barriers in the acceptance, decline and engagement in the management of musculoskeletal conditions. And also explore the extent to which new evidence on the perspectives, barriers and facilitators of the use of communication and information technology is currently being addressed to improve acceptability and engagement in the management of musculoskeletal conditions*)* | - | - |
| **Research priorities automatically included in Delphi's final list** | | | |
| 1 | *Research about how to implement telehealth services at the user, clinician and health system level* ***with patient/consumer involvement*** | 91.88% | 8.4 |
| 2 | *Effectiveness of treatment approaches delivered via telehealth in the management of musculoskeletal conditions* | 89.38% | 8.5 |
| 3 | The cost-effectiveness of telehealth treatments for musculoskeletal conditions ***considering different settings and income levels*** | 88.13% | 8 |
| 4 | Equity research on interventions to improve access, treatment or clinical outcomes to telehealth services for disadvantaged or historically underserved populations with musculoskeletal conditions | 88.13% | 8.3 |
| 5 | Design and evaluation of curricula to train students and health care practitioners in the provision of telehealth that conforms to core capability frameworks | 83.75% | 7.7 |
| 6 | Research on health literacy, eHealth literacy, technology literacy and identifying relevant factors for patients with musculoskeletal conditions engaging in telehealth (eg, barriers and facilitators) | 81.88% | 7.8 |
| Italics and bold items reworded based on panel members feedback; Description developed by coordinators and steering committee based on panel members feedback*. | | | |

**Comments and suggestions for research priorities provided by the panel members**

***Would you like to comment on the research priority of _"Effectiveness of treatment approaches delivered by telehealth in the management of musculoskeletal conditions"***

"This is a very broad question and it may be that interventions delivered 'live' via video conferencing differ from an ehealth intervention where a participant follows a self management route via internet materials with some or no telephone support. In addition medical consultations differ from physiotherapy and psychological therapy is different again. It may be that certain interventions are better suited to digital delivery and that certain outcomes are better than others"

"In the ongoing COVID 19 pandemic, avoidance of physical contact is paramount and it is preferable that patients are guided for exercise in their home environment which also achieves the goal of distancing."

"I think it would be relevant to differentiate the type of intervention to establish its effectiveness rather than investigating the whole set of telerehabilitation interventions together. This makes it very difficult to interpret the results, since very different interventions are compared (web program vs. videoconsultation, for example)."

"There is a big scope of conducting research in this area as it will be an effective tool in treating patients who cannot visit rehabilitation centre on regular basis and by this mean therapist can see patients staying in remote areas and can cater to more number of patients."

"Cardiopulmonary science"

"Effectiveness of telehealth interventions for MSK disorders when provided by specially trained clinicians"

"Telehealth has become an essential during the time of COVID. I want it to work as well as in person visits, but I do not know for sure if it is!"

"Investigate strategies to improve the effectiveness of physical exercise performed remotely"

"Patients face difficulty understanding the exercise program (instructions)"

"An orientation guide for the management and treatment of patients through Telecare in real time is needed, in addition to research in the area to verify the effect of this type of intervention."

"Given the explosion of telehealth, research priorities should focus on care delivered via this format"

"We must know the effectiveness of treatments offered in these modalities if we are to continue with them"

"Investigating digital solutions that support interventions in this sense is extremely relevant to improve the care offered"

"Scalable technologies to delivery MSK treatments are - so far - the only way to disrupt traditional delivery models…"

"Even though the COVID-19 pandemic has hugely reduced skepticism around digital health, there is still a dire need for more studies showing that digital interventions are not only well accepted but also effective in delivering high quality care- in single arm/cohort studies and also in comparison with so called conventional approaches"

"Make distinction between apps/web-based etc. "

"NA"

"Further research is required on the real-world effectiveness of various interventions to various populations"

"Cost-effectiveness studies are relevant in telehealth future researchs. It would also be of great importance to provide data on possible risks or adverse effects associated with their use. This information is important for Healthcare Systems."

"The wording is clear. Telehealth is becoming an increasingly well-recognised term within the general population - I support the wording of this priority. This represents a very important research priority given the increasing burden of musculoskeletal conditions on the health care system, which is potentially impacting the delivery of acute, urgent care."

"I think once research shows effectiveness of an intervention, if the fact it is provided by telehealth doesn't significantly alter it, or have a reasonable reason for thinking it may not be effective remotely, it may not need to be assessed for effectiveness as increasing number of studies show equivalence."

"The relevance of priority depends on country-based adoption of tele-health"

"Beyond the effectiveness of one type of intervention, it is important to define the profile of those who are willing to receive a telehealth treatment. Cultural aspects and the specific needs of populations must be taken into account when evaluating effectiveness."

"The aim is fine, but the approaches should be targeted to the population, by a healthcare professional who is trained in that approach, using a study design that is appropriate to research the question."

"This is a priority as it offers a potential method to increase access to care in resource poor countries"

"I would not use the term telehealth as this covers a big range of deliveries. I would use specific terms such as mobile health, internet-based, etc."

"Perhaps, instead of telehealth, synchronous and asynchronous eHealth intevrentions

more than effectiveness is needed, also patient engagement, feasibility, acceptability, satisfaction with new treatment approaches"

"Telerehab is well developed in high-income countries. Further research should investigate the (cost) effectiveness of tele intervention (different modalities) in low- and middle-income countries."

"Digital health interventions have been increasingly used recently and investigating their effectiveness is paramount! However, their definitions, differences and similarities also need to be further investigated, so the studies focused on testing their effectiveness can be appropriately conducted and summarized through systematic reviews."

"suggest emphasise this is agnostic to discipline and diagnosis, also need to look at difference between acute care and longer chronic disease management"

"I'm missing the question of study comparator in this priority. Comparisons both against respective face-to-face modalities but also vs. other controls, incl. sham., are important in different scenarios."

"Effectiveness, preference and ease of use..."

"Chronic MSDs and Geriatric related conditions"

"It is very much important to manage musculoskeletal conditions"

"In my opinion, the whole concept of potential Telerehabilitation advantages lies on its capacity of improving access and cost-effectiveness. We already know that education and exercise are effective for several musculoskeletal conditions, but how its delivery thought Telehealth might modify its impact in real world? This is the gap that must be bridged for me.

"Effectiveness of telehealth versus usual face to face care"

"Effectiveness is always important. I think the challenge is to agree on outcomes, i.e. what should the primary outcome and thresholds for effectiveness be?"

"It is important because it can show how effective telehealth is nowadays"

priority is important to, in case of proven effectiveness, increase access for patients in remote locations around the world to a trained musculoskeletal physical therapist"

"Effectiveness of telehealth approaches in the management of musculoskeletal conditions"

"No"

"yes it is very effective."

"As this is a new mode to deliver treatment, there is a necessity to undertake this research.

"Effectiveness of physiotherapy treatment delivered through online medium in management of musculoskeletal conditions"

"No"

"It is important to continue to follow patients after a rehabilitation period as a manteinance"

"No suggestion"

"No"

"no"

"no"

"no"

"Too diverse to be useful. Needs splitting."

"No"

"Tele Health is need of hour and hence really important for management of musculoskeletal conditions but it can not replace the physical contact required between health provider and receiver because physical contact help build confidence and comfort between them. "

"Not only in pandemia e-Health is important"

"I would to clarify whether this research priority only concerns clinical effectiveness or also cost-effectiveness. If it only concerns clinical effectiveness, I would suggest adding the word "Clinical" at the beginning of the sentence."

"Musculoskeletal conditions may be too broad; it may need to be addressed by body part/condition."

"The highest priority as far as I can tell."

"Given the pandemic, the world was forced into remote management of patients. Some schools of thought think, that the results were equal but there is also the argument that this intervention requires a steady internet connection and compatible device with the end-user not only proficient in its use but comfortable with a virtual visit. Research in the field will result in reliable information and recommendations to improve the service"

"Some patients do not thrive with telehealth and we need to identify these patients early.

"Not so popular now"

"Important to understand this treatment approach, given geographical and other challenges for many patients"

"I have seen some resrach so there may be a higher priority"

"I think that telehealth applications are applications that must be done in addition to face-to-face health applications, especially when there is a problem in the transportation of patients to health centers."

"we need more data about this topicç"

"I prefer: "deliver (partly) by telehealth"

"Although it is important but physical sessions are best and should be preferred for effective treatment of musculoskeletal conditions"

"Too broad, does not specify treatment content or medium or conditions"

***Would you like to comment on the research priority of _"Investigation of harms and adverse events during telehealth encounters for musculoskeletal conditions"_***

"Again too vague"

"An orientation guide for the management and treatment of patients through telecare in real time is needed, in addition to research in the area to verify the effects of this type of intervention."

"Apps are only licensed, if they do not harm. However numbers needed to harm are important

"Are there any harms or adverse events using telehealth for msk conditions"

"As with all health interventions, the risk-benefit ratio should be considered before use. This includes a thorough understanding of the safety profile of interventions. Telehealth approaches may be advantageous and feasible in many ways. However, a deeper understanding of how to appropriately assess tele-health specific harms and adverse events is required. Criteria for assessing harms for face-to-face interventions may not be directly translatable for telehealth settings. Further, clinical escalation pathways and processes may differ to face-to-face care, and this should be explored."

"Considering the prevalence of telehealth now, we need to look into if it is actually a good thing or not! "

"crucial for patients' safety, i.e. methotrexate administration"

"Does this intend adverse events 'resulting from telehealth encounters'? If so, perhaps language of that kind could be sued instead of 'During'. "

"Focus on ruling out Red and Yellow flags"

"For example, I would understand the n° of adverse events due to the execution of treatment (e.g. exercise) performed without the supervision of the therapist."

"Harms and adverse events are generally neglected in these pain therapies. We need to know who is not engaged by them, as part of potential harm."

"Harms and adverse events directly related to telehealth are not foreseen in my opinion."

"Harms and adverse events should be studied alongside the effects of eHealth interventions."

"Harms of missing interventions Can be important"

"Harms/adverse outcomes should be reported for any studied intervention (telehealth and otherwise) "

"I don't believe there are strong reasons to believe that harms would be greater than in face-to-face care. Also, maybe we should also look at the same time to the harms of face-to-face sessions (and of missing them) "

"I don't see this as a priority, but as something you always monitor during research in telehealth. "

"I think there have not been such research before and first we want to do no harm"

"I think this is highly relevant. Harms that we have experienced or aimed to mitigate for include; missed diagnosis, safeguarding (inability to express need via videoconferencing), ineffective treatment due to poor internet connection / distraction of trying to home school and receive treatment, data protection issues, declining (psychological) treatment due to not wishing to observe image and / or be in an online group. Usually we may meet people individually face to face and build confidence and trust prior to attending group based pain rehabilitation. We have also modified physical activity so that it can be conducted in a confined space and without much equipment- this may limit effectiveness. We don't tend to practice floor based activities with highly disabled people as we often cannot see them if off camera- normally we would practice this in person and be able to stabilise a chair for getting off the floor for example. "

"I think this research its most important than the previously"

"I would consider this as part of research on effectiveness, as to me effectiveness includes both benefits and potential harms"

"If it means receive intervention quicker I query the need for this. Need to research most effective approach and training to ensure a successful encounter"

"Important to do"

"Important to define "harms" in this space"

"Important to understand whether there is the potential form more adverse effects of treatment when delivered virtually. For example, do patients overdo exercises more often Or have increased chance of doing them incorrectly When the therapist teaches the patient to do these exercises virtually. "

"Investigation of harms and adverse events during telehealth encounters"

"It is good enough"

"it is need of the hour"

"It might come later or in parallel of the main researches about telehealth, but definetively it would not be my first priority of research"

"It sounds a bit weird to make a research priority on the harms. We want to investigate the effectiveness and, while doing so, identify possible harms. It would be similar to the pharmaceutical industry making clinical tests looking for adverse events to write down the side effects. But maybe I am misunderstanding the gap. "

"n/a"

"NA"No

"No"

"No"

"No"

"No"

"no"

"no"

"no"

"No"

"No adverse events will occur by adequate planning and careful implementation"

"No comment"

"Or perhaps something related to difficulties to adherence of telehealth? "

"Other family members try to do the same exercises provided that their condition and type of exercise they need to do might be completely different imposing questions over the effectiveness of intervention. "

"Our approach is to devide consultation half/half between video-consulting and live-consulting. So that hands on corrections can be given to patients. "

"Previous research provides evidence that Telehealth is safe and effective"

"Privacy should be one of the research topics. "

"proper physical supervision of exercises. "

"Safety is important in delivering health care. I suspect a better focus is on determining patient suitability for TeleHealth. i.e. scope"

"Safety measures for specific patients should be discussed along with clinicians"

"studies of effectiveness always need to investigate harms. "

"The Telehealth type should be defined as we should be sure the types of Telehealth"

"There is a need for studies to show the importance of telehealth applications in a controlled manner carried out by authorized persons together with face-to-face health applications"

"This investigation is very important as this will prepare the therapist as well as patient for potential risks and will help the therapist to take necessary precautions while guiding patients and while recruiting patients. "

"This is unknown so is important"

"This may be better worded as follows: "Investigation of patient-related harms and adverse events during telehealth encounters for musculoskeletal conditions."

"This should be embedded in RCT's"

"Unless there's direct harms for choosing telehealth over in-person health service, the word 'harms' can be replaced with another word such as '[safety] risks'. "

"Very important"

"we did a telerehab program on pain in the arm and legs; no problems happens if there is a first step de visu and than the possibility to go on in tele health. "

"We must be understand harm and adverse events in order to mitigate risk"

"What are harm/ adverse events? Or is it about delayed diagnosis of serious pathologies? Also, considering ethics, how could harms be investigated if video consultations are kept confidential? How could we minimise the under-reporting of these adverse events during the research? "

"When the professional is not present the patient can injured himself"

***Would you like to comment on the research priority of _"The cost-effectiveness of telehealth treatments for musculoskeletal conditions"_***

"As before"

"Comparison of standard face to face msk care versus telehealth msk care"

"Cost effectiveness for patients should also be included (in addition to health system cost-effectiveness, etc) "

"Cost effectiveness priorities should consider direct/indirect patient cost as a priority"

"Cost-effectiveness should be a priority on the research in low- and middle-income economies"

"cost-effectiveness studies should be embedded with RCTs evaluating effectiveness and safety of Telehealth interventions"

"Costs might not be economical as compared to physical consultations compared over effectiveness"

"Efficacy of the approach needs to be securely ascertained before effectiveness can be put in the balance wth cost"

"Environmental impact for me is more important than cost-effectiveness"

"Good enough"

"Health system financing has to be taken into account, as this varies a lot between different countries"

"Hopefully cost-effectiveness includes cost benefit analysis to patient and community, rather than just straight financial analysis. "

"I agree this is important but several factors change rapidly and may render findings quickly outdated such as cost of equipment, access to certain technologies, organization of health services etc. "

"I don't think teleconsultation is cost effective. "

"I miss the terminology 'blended care' in which telehealth is integrated within usual care. (this comment belongs also to previous questions, but unfortunately I can't go back in the system). Regarding this priority: from societal perspective it's always good to take in account both costs and effects. "

"I think if it is not shown to be cost effective it will not happen"

"I would say not only cost-effectiveness but also social return on investment. "

"If a modality is too expensive or does not have an appropriate cost benefit ratio - it should not be used"

"Important"

"Important consideration when ramping up models of care"

"Important to drive policy"

"in health systems such as Brazil, for example, with insurers and medical plans that pay health professionals very poorly, this type of research is a challenge and also its implementation"

"It is also a need of the hour"

"It is cost effective"

"It is cost effective but if the pain intensity in VAS is more than 7 then patient has to come department physically to take treatment. "

"It is important both for patients and for health service"

"It is important to consider the contrast in effectiveness and cost-effectiveness studies"

"It is much needed because although telehealth seems to show promise in this field, it is not yet clear that this is being achieved on a widespread basis. "

"it reduce the health cost of patients. "

"Look at the separate delivery modes (apps, internet, etc.) "

"More important than cost-effectiveness is the question of whether remote consults can improve access to care or what can be done to get that effect; I hope this will come up later. If not, I'm hereby suggesting it be added for the next round. "

"NA"

"New healthcare delivery models are needed more than ever, but at the same time there is a strong need to contain increasing expenditure. Digital health can be the key to solving this, and in my view the cost-effectiveness piece is the missing link for that. "No

"No"

"No"

"No"

"No"

"no"

"no"

"no"

"no

"No comment"

"Quality will always be the most important in my opinion. "

"Research in the area is necessary to verify the feasibility of this type of intervention. "

"Should be done routinely alongside effectiveness trials"

"Similar to my previous comments, cost-effectiveness forms part of the risk-benefit assessment of health interventions. In several countries around the world, there have been federal initiatives to subside telehealth appointments. The impact of this should be assessed. This priority is worded appropriately. "

"Tele-health is likely cost-effective, however do patients prefer this method of delivery? "

"Telehealth, if effective, offers a potentially cost-effect method to deliver care in resource poor settings"

"The challenge here is measuring effectiveness of TeleHealth and a suitable comparator service. Cost is relatively straight forward to measure. "

"The types of Telehealth treatments may play a role in cost-effectiveness. Environmental difference may also come to play as the costs may differ from one environment"

"They have been rolled out without consideration of efficacy, especially whether they substitute for face to face healthcare (expensive) or supplement it"

"This is highly relevant as it will be viewed as being a cheaper alternative for a pressured healthcare system and may potentially expand access to treatment. "

"This is paramount to the continuation of this model of care beyond the pandemic. "

"This may be challenging with different funding models across countries; however, is important work. "

"Until we address other issues, cost is less important"

"Video conferencing"

"We know Tele is cost effective, but there are few studies reported on this topic"

"What cost are you talking about? Still uses as much clinicians time but more costeffective for patient and long term if able to access treatment quicker may reduce need for input"

"Whether these are cost effective or not is still a matter of debate"

"Yes, as everyone can't afford or able to operate smart gadgets"

***Would you like to comment on the research priority of _"Equity research on interventions to improve access to telehealth services for disadvantaged populations with musculoskeletal conditions"_***

"access to and accessibility of - a subtle difference but it is not just about getting linked to services, but also the ability to participate in them fully"

"all other priorities cannot really be viewed in a global context if lack of equity and access to telehealth is a determining factor"

"CALD, older populations and those with poor access to technology / digital literacy are at risk of being under-serviced in a TeleHealth service model"

"Digital health interventions can be used to improve access to care in particular in these populations"

"Disadvantaged populations face additional or different challenges towards health care access."

"For example, telehealth, which often relies on secure internet or phone connections, may be challenging for people living in rural areas who often have reduced or unstable connectivity. The impact of this should be examined to optimise the design and delivery of telehealth interventions. "

"equity in health and research is a growing topic and is especially important for the world that is becoming more and more virtual. "

"Here health literacy might be important"

"Highly important as this technology and the digital approach has larger potential to be "mass" distributed and benefit those who do not have access to other types of healthcare. (if effective and safe, of course) "

"How do we overcome barriers to telehealth msk care for disadvantaged populations"

"I think equity is a general issue for all treatments and not just telehealth so should be looked at in that context"

"I think less advantaged persons and people in remote places need it more so we should know it works for them"

"I would refrain from the word 'disadvantaged' and use other generalized word in research equity such as 'underserved populations'"

"I’ve been a part of a pro bono clinic during the pandemic where we provided care via telehealth. We ran into many issues with accessing care via telehealth - no device, poor connection, etc. "

"If you think economically backward class, delivery of telehealth is going to be difficult

"Important as per present scenario"

"Improving poor populations' access to quality musculoskeletal care should be the top priority for any scientist"

"Internet literacy could be great to study before apply any intervention with telehealth. "

"It is essential for outreach and better coverage"

"It is little addressed; such populations might find the interventions more accessible than face to face, but we don't know about the financial, technological, privacy and other barriers. "

"it should be uniform and mainly rural based. "

"It's important"

"Make it work for 'general population' first. Thereafter, tailor to disadvantaged groups. "

"NA"

"No"

"No"

"No"

"No"

"no"

"No"

"no"

"No comment"

"No comment"

"no doubt about this, tele health services improve access. "

"None"

"Not important"

"nothing to add"

"One of the benefits of telehealth tools is the ability to democratise and reach population segments where healthcare is often scarce. The use of affordable and accessible devices such as smartphones, computers and tablets should be a priority. "

"perhaps underserved instead of disadvantaged? "

"Previous comment was based on this concept. Please refer"

"Remember that in many sistuation it is quite difficult to let these patients come to hospitals to have care"

"Research in the area is necessary to verify the positive effects of this type of intervention.

Scrap my comment from above. "

"Technology may not result in bigger differences in for example health between populations"

"Telehealth, which promised to be a tool to bridge the inequity gap in health, is not being able to solve it, since there is inequity of access for economic reasons or digital literacy in the most disadvantaged populations, who could benefit the most due to the difficulty of accessing health care in person. "

"The poor and marginalized are those persons who would lack a device, or the knowledge to use a device. They may also be the ones stigmatized during a session resulting in an ineffective intervention. "

"There are inequities with virtual care as marginalized populations do not have the infrastructure/resources to support virtual care. If you identify this via research, it brings awareness but efforts should be directed at solutions. "

"There are many poor people who don't have access to health care. So, telehealth for these people is a great solution"

"there are specific barriers to telehealth interventions in disadvantaged populations which may inadvertently lead to further disadvantage"

"There is not much research in this area and there is general assumption that technology will increase access to care while it may not always be the case and could increase disparities. We should better understand the equity implications"

"This could be reworded as follows: "Equity research to determine barriers and enablers to access to telehealth services for disadvantage populations with musculoskeletal conditions."

"This is a complex issue. Some of the complexity lies in people not being referred for treatment / being aware of how to self-refer. It is difficult to provide a culturally competent service to all underserved people- a possibility is that there could be a national / regional digital service that could provide more culturally appropriate interventions - interpreters are not enough to provide this. We ask people what pronoun they would like to use at the beginning of treatment to indicate openness / acceptance. Again access to treatment is likely to be an issue here. Some people have informed us that they attended an online group but would not have attended in person (for reasons related to anxiety / fear of judgement). They said the experience was (unexpectedly) 'life changing'. "

"this is the key advantage of e-Health....you can deliver it to any patient around the world"

"this is very relevant as many of the interventions reach people with high health literacy instead of low health-literacy. the applications should help closing this gap. "

"This must include a look at the provisioning of access for these populations, e.g. providing devices and internet connection"

"This would be among my main research priorities"

"Under serviced populations lose out if not enough research is done to understand their needs"

"Understanding the barriers for these disadvantaged populations to access telehealth is necessary"

***Would you like to comment on the research priority of _"Design and evaluation of curricula to train students and health care practitioners in the provision of telehealth that conforms to core capability frameworks"_***

"absolutely; we need to start from students and increase culture in GPs"

"All HCPs and students must understand how to use different modalities and be able to use them appropriately"

"Anecdotally, clinicians report that the provision of telehealth care is not necessarily synonymous with face-to-face care. Different skills sets are required, and further training may be needed to support clinicians in the uptake and delivery of telehealth interventions. Without context, it is difficult to determine what core "capability" frameworks refers to. "

"Considering the differences in curricula and approaches across various cultures, age groups, etc., I don't think design of a curricula is of great priority. While the idea is good, I don't think the real-world application will be as feasible. "

"Design and evaluation of curricula to train students and health care practitioners in telehealth that conforms to core t"

"Capability framework"

"Designing core capability frameworks for telehealth approaches"

"Due to its rapid expansion due to the COVID19 pandemic, it is necessary to include curricular training in digital practice, including not only teleconsultations but also exploring different technological options to, for example, standardize data collection that can facilitate access to data for researchers. "

"Effective education is extremely important to support the uptake of Telehealth"

"Encourage patient partners/consumers to be involved"

"Good initiative"

"Health professionals often require a process of learning and reflection to use and make the most of telehealth tools, sometimes involving a complete change in their standardised way of working. These processes could be accelerated or eliminated with prior training. "

"Priorities such as basic training in Medical Devices, Digital Therapeutics (DTx), regulatory systems for medical devices, processes for adapting to new technologies in clinical practice, etc."

"I believe without research, this is worth implementing"

"I think that in some programs the curricula could do a better job of just teaching future physios to communicate, and then telehealth could/would be better. "

"I think we need to know it does no harm and works before putting it in the curriculum but when it is it will go up in importance"

"Important to standardised the quality and safety of delivering telehealth"

"in order to effectively and safely deliver Telehealth, embedding Telehealth in curricula is critical! "

"In order to guarantee the external validity and the implementation of telehealth solutions in real world, this would be a very important research topic after comprovating the cost-effectiveness and accessibility of telehealth"

"It is needed but not be only option as medical education require more physical interaction to achieve better outcomes"

"it is very important to train students because telehealth is diferent from face-to-face consultation"

"It should be a priority especially in these difficult times"

"its seems good"

"NA"

"Need to answer effectiveness questions first"

"Need to clarify if you mean professional competencies here explicit to telehealth, or applying telehealth across competencies"

"No"

"No"

"No"

"No"

"No"

"no"

"no"

"no"

"No comment"

"not clear to what core capability frameworks you refer"

"nothing to add"

"Once we understand this model of care better, it’s time to engage and train students To deliver this care effectively. "

"Quality and standards are important"

"The curricula should also include the ability to perform a physical examination virtually. This can be challenging for students who are learning to do an in-person physical examination. "

"There is enough evidence and training available to create a curricula for the students, specially based on WHO guidelines for telehealth implementation"

"There needs to be comfort with an in-person exam prior to transitioning to virtual. Not sure if the emphasis for students should be to be confident in virtual as a new learner. "

"This is a fast moving target, and our students will have different experiences and assumptions from ours. I'm not sure formal teaching is the way to do this. "

"This is highly relevant. Although there are publications related to good practice, in 2020, there was little about live videoconferencing techniques for rehabilitation. We need standardised guidance on data protection. Even the trust where I work does not understand the practicalities around protecting data when working in groups. We had to undertake practice as a team to work out how to do this. I have learned how to complete a data protection impact assessment (by experience not teaching). Students and clinicians are very unaware of the governance requirements. The British Pain Society is due to publish guidance regarding pain management programmes, but this (when I last read it) does not cover legal aspects of data protection. This information should be coming from the 4 national health boards such as NHS England in the UK. There is no uniform guidance on a safe platform for group videoconferencing. Anthony Gilberts research is helpful in highlighting these issues- discussing insurance etc. We need frameworks around competent delivery, intimate examination, clinical risk, safeguarding. We have written SOPS for some of these as a department. "

"This question is a relevant topic. I have direct experience with this. For example, at the university level in Italy, we are checking the educational curricula to understand if they conform to core principles suggested by WCPT educational framework. "

"This would be very helpful for universities and curriculum design"

"Those that want to use the existing technologies, will educate themselves through existing courses. Researching this will contribute little to the inrinsik motivation of people in this. "

"To early"

"Very much important for students"

"we must prepare undergraduate physiotherapy students for what will come in the future"

"What are the core standards needed for safe and effective msk telehealthcare"

"yes it should be. "

***Would you like to comment on the research priority of _"Telehealth implementation studies in musculoskeletal conditions at the user and health system levels"_***

"A framework to implement telehealth msk care provision"

"Another piece of the puzzle needed to Implement this model of care. "

"As previous comments. "

"Essential to train and educate healthcare workers in use of telehealth, otherwise the advantages the technology offers will not be seen (it will remain something extra besides the already existing burden). "

"Health system and policy research is sometimes forgotten and if not studied can be a barrier to implementation"

"Here I take a capitalist view: let innovative companies determine how best to implement telehealth and learn from their successes. "

"Implementation is tough, often specific to countries and their healthcare systems, so lots of good interventions go unused. "

"Implementation is very much important"

"Important"

"important"

"Important not to stop at effectiveness in controlled trials but to move into how it is taken up in practice and the fidelity to the original interventions"

"In our country, there is a lot technology available but hardly not used. "

"Inclusion of 'clinician' or 'health care provider' as part of the priority, since the main objective is supposedly "Research about how to implement telehealth services at the user, clinician and health system level".

"Interventions only effect change if they are indeed implemented and used"

"It is very interesting as there are multiple barriers to its use, both personal and structural, and finding a workflow to get it into clinical practice and accompanying it until its integration is crucial for its speed of implementation. "

"It needs to be done correctly when we know it can work for all parties"

"It seems good"

"It will ease of doing"

"NA"

"no"

"No"

"No"

"No"

"No"

"No"

"no"

"no"

"no"

"No"

"No comment"

"No comment"

"No comment"

"Not sure if there is a need for implementation. People figured this out during the pandemic. The focus should be on where do we go from her. Sustaining virtual care, what population would benefit the most, how can we incorporate it once the pandemic is not the driving factor for participation in virtual care. "

"One of the limitations that can be encountered when implementing telehealth systems in clinical practice, and why they fail, is the process of change. "

"As professionals working in this area, we have the responsibility to provide instruments that facilitate this change that the use of a new digital tool entails. We must guide the process based on previous successful experiences, train professionals in the use of the tool, and provide keys to explain and deal with patients through these tools. "

"If we leave a professional alone with a digital tool they barely know, they are likely to abandon its use."

"Patient/consumer involvement is essential"

"Perhaps reword as follows: "Telehealth implementation studies at the user and health system levels, for musculoskeletal conditions."

"Relevant"

"Similarly to my answer in the previous question, this is a paramount research topic for implementing the solution in real world scenarios"

"studies on effectiveness, cost-effectiveness and safety of Telehealth interventions aren't sufficient to influence practice and policy without implementation studies. "

"the implementation is rather the most important step to make it available to broad range o patients"

"Uptake of Telehealth services has been slow and there are many barriers that need to be better understood"

"user suggests micro level (clinician/patient), system suggests macro, but what about the meso level (health services and workforce) "

"yes it brings improvements in patients health. "

***Would you like to comment on the research priority of _"Research on suitable outcome measures for telehealth in individuals with musculoskeletal conditions"_***

"A common discussion in research of musculoskeletal conditions is centred around whether the appropriate outcomes are being measured (e.g., are they meaningful to patients and clinicians?). This priority is highly important. "

"agree - if this is the mode of delivery the outcome measures need to be valid and reliable"

"As a clinician, there are validated patient-reported outcome measures to use. Although this is open for debate, or whether we need to standardise delivery of physical tests for telehealth"

"At first we need to compare telehealth with the same/similar outcome measures as used during in person care. Only when the efficacy of telehealth is shown can we begin looking at new or unique outcome measures. "

"Balance between telehealth and contact bases health interventions is necessary"

"Find out the optimal evaluation tool"

"Good"

"Ha, ha, just mentioned it above"

"Highly relevant to see more relevant OMs"

"I believe that the clinical measures used today (namely PROMs) already cover most of the needs"

"If we do not know what we are measuring for outcomes - we will never know if we have been successful"

"Important"

"It would be good to examine suitable outcomes on care being delivered via telehealth"

"It's unclear how this would be different to outcome measures used in F2F care. "

"Lower priority, can start with accepted outcome measures for the conditions"

"Make sure to include patient oriented research outcomes. "

"Many of the outcome measures may be similar to what we would use if seeing people in person, so suggest this is less of a priority. "

"Must be patient centred similar to PSFS"

"NA"

"no"

"No"

"No"

"No"

"No"

"no"

"no"

"no"

"No"

"No comment"

"No comments. "

"Pain intensity could be replaced by other primary outcomes such as QoL, burden, etc"

"Patient reported outcome measures could be helpful"

"Suitable outcome measures should be determined with patient/consumer partners"

"The outcome measures in musculoskeletal pain that we are already using in conventional practice can be extrapolated to the digital one"

"There are hardly any validated clinical questionnaires in digital format, and work is needed to assess changes in psychometric variables. "

"there are many measures already and we do not need specific telehealth measures, but to explore the measures already have"

"this is a very important point to work in line with VBHC"

"top priority"

"Valid and reliable outcome measures to use in an msk telehealth service"

"we aren't able to accurately assess the effects of interventions without reliable and valid outcome measures. Also, it wouldn't be right to assume that outcome measures that are valid as paper-pen administration will also be valid when administered online. This to me a prerequisite before testing any Telehealth interventions. "

"we can look at the same outcomes as in person if we compare to in person first then they may be different"

"We know a lot about suitable outcomes: why should telehealth have different ones? "

"We need to include physical outcome measures (for physical rehabilitation interventions) / qualitative alongside PROMs and economic evaluation. "

"Will be hard to have valid trial results without being confident in our measures"

"yes ,proper interval based outcomes should be checked. "

***Would you like to comment on the research priority of _"Research on diagnostic tests suitable for telehealth in individuals with musculoskeletal conditions"_***

"Applicable to promote widespread coverage"

"Can't see it as a really worthy cause"

"Considering that orthopedic tests are not good at isolating the structure in a large percentage of cases, I do not think it is a priority to compare them with those self-administered by the patient. "

"Diagnoses is not the key option for telehealth. Treatment comes first! "

"diagnosis informs and shapes treatment"

"Diagnostic tests shared via telehealth are very similar to in-person as the Xrays/MRI results/blood work would be the same. Objective tests should be verified. Is it still positive if self-administered. "

"Essential research to optimise the patient journey and ensure appropriate patient referral"

"Good"

"I believe it is important to digitise some commonly used tests (like the 5 times sit to stand test) but not crucial"

"I don't think diagnostic tests are particularly helpful in promoting musculoskeletal health"

"I personally think there are many services that can be done through telehealth but real-time virtual diagnostic may not be one of them. Assessments for MK conditions diagnostics may just be few of the essential services best provided in-person if able. "

"I’m not sure what this question is referring to. If I think that a patient needs diagnostic tests, then I would refer them to the appropriate care provider. Imaging, etc can only be done in person. "

"Assessment of pain could be done remotely"

"If we are going to do business in a different environment - then we have to understand what diagnostic testing is suitable to that environment - and what isn’t"

"It is clearly mentioned"

"It is necessary to standardize tests"

"it is very important because tests are probably quite difficult to do at distance... for this reason we need to study this problem"

"May want to reword as follows: "Reliability and validity testing for diagnostic tests suitable for telehealth (compared to in-person testing) in individuals with musculoskeletal conditions."

"Most diagnostic tests are unreliable how about concentrate on listening skills and testing clinicians ability to listen to the patient"

"NA"

"NA"

"No"

"No"

"No"

"No"

"no"

"no"

"no"

"No"

"no comment. "

"Not clear whether you mean technology to measure or certains ways of examination via the screen. "

"not my area of expertise"

"not specific to telehealth"

"one of the most difficult points for implementing telehealth is the difficulty for physical therapists to perform a quality fully hands off physical exam"

"Shared decision making for telehealth in individuals with musculoskeletal conditions"

"Specification of the test is needed here. Not all muscoluskeletal conditions are suitable for self-assesment by patients. "

"Telehealth diagnostic testing, validity and reliability testing"

"The initial evaluation to verify the eligibility of patients to undergo treatment in telahealth should have a specific protocol to ensure the success of the treatment. "

"The tools used in a face-to-face encounter may require modifications to be effective in a virtual encounter. Therefore, there is need to examine those so one can adapt. "

"This need to be undertaken to manage safety and clinical risk. "

"To implement 100% online telerehabilitation, it is very important that the assessment is also carried out online. Thus, it is necessary to validate the tests to allow their application. "

"Yes important"

***Would you like to comment on the research priority of _"Identification of patient characteristics (moderators) that affects the response to treatments delivered by telehealth"_***

"Again, for me, if we are to delve into this, than the scientific community should, in parallel, delve into the identification of patient characteristics that affect response in conventional approaches. Essentially, we should not assume that conventional is the right way and have to prove digital is up to that standard. "

"Can be a part of any other reasarch"

"First effectiveness"

"Good"

"Good"

"i think this would fit into some other research priorities"

"I would say not only patient characteristics but also context characteristics. The context of the patient can also (indirectly) affect the response to treatments delivered by telehealth. "

"Identifications is important"

"Identify for whom telehealth might work is an very important research topic, mainly to assist clinicians in decision making about which patients or clinical conditions could be addressable through telehealth"

"If we aspire to personalized health care, it is important to assess which patient characteristics are key when offering telephysical therapy"

"Important to know which types of patients respond better than others"

"Important to unserstand wich intervention works when and for whom at what time"

"In order to provide personalized care, it important to know which patient is suitable"

"In person or remote, this information is very important. "

"Including social determinants and health literacy"

"Information on clinical decision tools, factors affecting rehabilitation (including social / financial) would be helpful. Factors such as a change in catastrophing and fear avoidance are known to moderate outcome in PROMS post pain rehabilitation but we don't know how important these factors are to individuals receiving treatment so this should be considered (in my opinion) "

"NA"

"no"

"No"

"No"

"No"

"No"

"no"

"no"

"no"

"No comment"

"Not all patients will deem telehealth interventions accceptable, which may affect treatment outcomes. This should be investigated, not only through quantitative research but also qualitative research designs. "

"Only patient that require telehealth should be targeted with suitable selection of subjects"

"Perhaps "determinants of telehealth tools usage and effectiveness"

"subgroup analysis of who will benefit most will compliment equity and accessibility work

"The profile of people that use telehealth is crucial to understand the needs of that specific population"

"These studies could be embedded within observational studies or clinical trials. "

"This is a problematic oversimplification of the many interacting variables that affect treatment response. It is usually done retrospectively, overfitted to the model, and dependent on who entered the study, with no reference to those excluded. It is therefore usually unreplicable. "

"This is important because physical examinations are limited"

"This is very broad; should it be broken down to medical characteristics, physical characteristics, psychological characteristics, etc.? "

"This point is relevant. For example, I would understand the role of patients' characteristics (e.g., beliefs, expectations, preference) in affecting therapeutic outcomes. These elements represent Contextual factors capable of influencing the patients' response. "

"We need to establish efficacy and cost before working on moderators"

"we need to know who it will work for all ages??? "

"What are the key psychosocial characteristics which would predict successful outcomes for msk telehealth"

"While this is a priority, this can be merged with the previous research priority on building curricula - patient characteristics is one of the factors to consider as part of building a curricula and treatment plan. "

"yes it affects the outcomes. "

"Yes, we need to know who does and does not benefit from virtual care"

***Would you like to comment on the research priority of _"_Identification of clinician characteristics that affect response_"_***

"As the previous question, I strongly suggest the analyses of clinicians features. Evidence (e.g. papers from Rossettini's research group) inform on the value of these contextual factors capable of modulating the therapeutic outcomes. This is a good point! "

"Buy in from clinicians is important"

"Can be a part of other research"

"Clinicians characteristics and beliefs"

"Comfort level with technology is key to address. "

"I am thinking, clinician's training and not necessarily their characteristics, would affect a response. "

"I can't imagine identifying these characteristics can help the overall quality of telehealth in MK conditions. For instance, if a clinician's education level or cultural background affect response, what changes can/will be made from a policy-level that can address this? "

"I dont think that this is important"

"i would have the same comment as for the previous question, it likely fits into another priority (e.g. implementation) "

"I’m curious about this in all patient encounters, including in person! "

"important but there were others first as it should be the same basically as in person. we should be able to do both"

"It is helpful for clear diagnosis"

"More honesty and ethical and moral responsibility is essential"

"NA"

"No"

"no"

"No"

"No"

"No"

"No"

"no"

"no"

"no"

"No comment"

"Perhaps combine the patient and the clinician response studies"

"Physiotherapy attitudes and beliefs and the effectiveness of msk telehealth service provision. Mixed method study. "

"readiness to change and PBE in health professionals should be investigated too"

"The response in terms of outcome of tele physiotherapy? "

"Therapist that are uninterested or non-confident in remote-guidance of patients will not use the technologie. Thus research to this group is not usefull. "

"There may be particular skills for e-health engagement of patients, and response to them"

"This addressed somewhat in the conventional literature"

"this is a very important topic and often forgotten"

"yes - clinician preferences and comfort with delivery of care impact virtual care

"Yes it's affect

"yes.

***Would you like to comment on the research priority of _"Identification of mediators that explain or contribute to the mechanisms underpinning the effects of telehealth-delivered treatments (eg. self-efficacy)"_***

"As the same question.

"Because of the wide range of technologies and rapid evolution of technologies I'm not sure this would be feasible"

"feedback of patients is urgently needed"

"Good"

"I agree this is important, and I believe it comes hand-in-hand with understanding patient and clinician factors which may moderate treatment effects. "

"I think that the contextual factor will not change. Its the same as a presencial visits"

"important for optimizing interventions for individuals"

"Important to deepen our understanding of mediators in success or non-success in virtual treatment. "

"important to understand change meachnisms by different means of delivery"

"important, but not specific for telehealth"

"In my opinion related to the research priority on the patient characteristics"

"In order to further improve the available interventions, identify putative mediators of action is paramount"

"I've already commented on this. In my opinion we need to revise current PROMs (moderators) in pain rehabilitation to make them more relevant to the lives of people receiving treatment. Also to be aware that moderators imply factors in the individual that moderate treatment outcome when we know that social factors are very important as determinants of health. "

"Knowledge of these mechanisms may allow the development and improvement of the characteristics of the same, with emphasis on those processes most relevant to the patient. "

"Maybe not the highest priority..... "

"mediators brings patients closer"

"Mixed methods research could be particularly valuable (this is likely also related to patient and clinician characteristics) "

"NA"

"Não"

"no"

"No"

"No"

"No"

"No"

"no"

"no"

"no"

"No suggestion"

"No suggestions. "

"Not bad"

"Same as for 2 questions before, on predictors in patient characteristics. "

"Seems okay"

"self - efficacy is important part of research"

"Should be done routinely alongside effectiveness research"

"Suggest re-wording"

"these studies should be embedded within clinical trials (or observational studies where suitable) "

"This sentence is difficult to read. Perhaps "Identification of mediators contributing to the effects of telehealth-delivered treatments".

"This will help us in treatment priorities"

***Would you like to comment on the research priority of _"Research that examines the specific contribution of communication and information technology to the effectiveness of telehealth treatments in musculoskeletal conditions"_***

"As the same question. "

"Better communication and information sharing should be promoted"

"Cell phone, computer and specially internet are tools necessaries to the effectiveness of telehealth treatments"

"Communication software comparison study, patient and clinician satisfaction study

"Digital skills"

"do you mean the effectiveness of telehealth or the effectiveness of treatment in musculoskeletal conditions? The last option seems to be more logic. "

"Don’t really have enough information to understand what would be studies but communication is always important! "

"Due to the speed of technological change we are experiencing, it is difficult to make comparisons. "

"Human connection is important. Can virtual care still make a connection with the patient and deliver compassionate/empathetic care"

"Information technology is important in present time"

"it should be done. it seems okay for me"

"Lack of understanding around this may lead to a huge waste of resources. may of our patient opt for Telemed at first appointment (often due to confidence / convenience reasons) and then we need to see them in person to assess effectively - this creates more work, increases waiting times. We offer choice of digital and in person treatment, 90% opt for face to face treatment after the initial assessment. "

"Mixed methods research could be valuable here as well, particularly learning from the experiences of clinicians and patients engaged in telehealth"

"NA"

"Nao"

"No"

"No"

"No"

"No"

"no"

"no"

"no"

"No comment"

"No suggestion"

"Studies that can help address what part of telehealth services can replace in-person health service would be beneficial in future guidelines for treatment (e.g., SMS can replace in-person visits for prescription and lab results, email from physician may best replace for explaining diagnostics, virtual conferencing may replace education sessions and speech language therapy but in-person health may be warranted for physical assessments, etc.) "

"Telehealth is broad, and improving understanding of comparative effectiveness of different approaches/modalities available is necessary to reduce resource burden (e.g., funding, researcher time) "

"That is more interesting - the interplay between medium and content and outcome"

"The results of such research will be out of date by the time they are published. "

"There are many options out there and they are not all equivalent in terms of the rehab specific offerings"

"This is quite relevant as this can impact the telehealth session. "

"This is similar to previous topics raised by this questionnaire"

"this seems very specific; would it not be better as a sub-section of the implementation priority question? "

"Very important"

"Would this include materials/information/communications shared with patients prior to telehealth encounter. "

"yes information technology should be easily available for all. "

***Would you like to comment on the research priority of _"Explore health literacy, eHealth literacy, technology literacy and identifying relevant factors for patients with musculoskeletal conditions engaging in telehealth (eg, barriers and facilitators)"_***

"Although it can be answered by joint study, it is better to separate"

"COVID has illustrated the huge gaps in health literacy and numeracy; health literacy is inversely related to increasing age so this is a problem we need to address in our aging society; this may also impact some groups disproportionately relative to their health outcomes"

"Educate before implementation"

"Extremely important topic. "

"funding is the main barrier. "

"Health literacy should be a part of government policy"

"I am not sure what 'relevant factors' refers to? Is it barriers and facilitators? If so, I would enter these words in the statement. "

"I am pleased to see this as a priority. Health beliefs should also be considered. "

"I think health literacy has not been a very useful variable so far, and we should stop hoping it will change. I don't think it's a very psychological concept, more a managerial idea. "

"I would like to contribute to this research as a researcher"

"If telehealth is going to be promoted/justified after the end of COVID, then there should be some evidence that it is superior (or at least comparable) to in person visits. "

"Important"

"Important to understand these factors especially with regards to health equity. "

"It seems okay"

"likely included in implementation"

"mentioned in previous answers"

"Mostly important to understand barriers from resource limited countries"

"NA"

"Não"

"no"

"No"

"No"

"No"

"No"

"no"

"no"

"no"

"No comment"

"no different than normal"

"No suggestion"

"Not specific for musculoskeletal diseases. Should be broader. "

"Patient/consumer partners should be involved in identifying relevant factors for patients"

"Qualitative study of patients on telehealth msk health provision"

"this could make the difference as to whether it works or not"

"This is an important topic that would help to minimize inequity while using eHealth solutions"

"To early"

***Would you like to comment on the research priority of _"_Translation, dissemination, and communication of telehealth research findings for all stakeholders_"_***

"Already discussed"

"Due to the rapid spread of the use of telehealth tools, and even more so after the COVID19 pandemic, implementation processes are often very different from country to country, or even lacking. In addition, the staff involved are often not trained in telehealth. "

"Providing international guidelines and standards of use and quality can speed up the process of digital transformation of healthcare systems."

"Emphasis on situated experience (not individual lived experience, but the situated experiences of groups or populations) of stakeholders"

"Expanding upon this, patients and clinicians should be involved in this process to ensure that information is being disseminated appropriately and effectively to the public. "

"From a North American perspective, I feel strongly against the usage of the term 'stakeholders' as Native persons may find it inappropriate, due to its deeply rooted history in colonial practices. I would consider rewording such as collaborators, parties involved, etc. "

"Good"

"Good for future"

"Key point - with stakeholders, and that must include patient partners throughout the whole research process"

"Knowledge transfer tends to take a backseat, but the research is unhelpful if the results don't make it to end users. "

"NA"

"Não"

"No"

"No"

"No"

"No"

"no"

"no"

"no"

"No comment"

"No suggestion"

"No suggestions"

"Not a priority"

"Okay"

"Stakeholders should come forward"

The collaboration of local stakeholders is paramount for the implementation of Telehealth in health systems around the globe"

"The communication have to be simple and easy to understand"

there should be proper funding. "

"This is probably very difficult to draw general conclusions from, and conclusions specific to the telehealth and the stakeholder are not much use. "

"Too many studies never translated into care. This is an important consideration that must be taken into account from the very beginning. Engage key stakeholders early""too often research stops after initial effectiveness studies"

"we need all staeholders to buy in so they need to know about it and the efficacy"

"What clinicians like in new research dissemination"

***Would you like to comment on the research priority of _"_Identify, explore, and implement the most suitable business models to support the delivery of telehealth treatment for individuals with musculoskeletal conditions_"_***

"A big challenge for dissemination and implementation of telehealth"

"Again, I think this is best done in an observational way, allowing tech industry to sort out the best models. "

"Business models may be too region-specific to make it a top priority, considering much research on telehealth have to be done prior to successful implementation. "

"Cost effectiveness study"

"Finance is a big barrier, therefor important to share knowledge"

"Global differences will make this type of research challenging to generalize. Valuable for local contexts, though"

"Good"

"I dislike (though acknowledge the importance) of business models in healthcare. "

"I thought this might be part of the system or service level implementation question"

"I wouldn't call it a "business" model. This would encourage clinicians to consider clinical care as business rather than "service". This could be detrimental to evidence-based care. "

"If business models for support are not identified then we will never be able to implement with success and sustainability. "

"Implementation is important"

"It is good. This is very priority area of research"

"It is interesting. I would understand the feasibility of telehealth both in private and public health care services. "

"NA"

"Não"

"New solutions must have sustainable business model to be implemented in real world scenarios. Otherwise, this solution will be only adopted by public entities and will not reach its full potential of implementation. "

"No"

"No"

"No"

"No"

"no"

"no"

"no"

"no"

"No comment"

"No suggestion"

"No suggestions. "

"probably important, but too far away from my expertise"

"proper funding"

"this is always important but not as important yet"

"Ties in with health system research"

"Whilst this is important, I believe it is less a priority compared to the other points raised. "

"Why would this help? And how generalisable would it be? "

"yes, this is very important"

***Would you like to comment on the research priority of _"_Standardization of telehealth-related terms and the development of frameworks for categorizing different telehealth approaches in musculoskeletal practice_"_***

"Frameworks for categorizing approaches" is somewhat vague, and difficult to comment on without more background information. "

".não"

"Already commented"

"Common taxonomy will support evidence synthesis"

"Eventually yes we will need to standardize language however usually a term is defined to clarify and prevent assumptions"

"Frame work development is important"

"Framework/guidelines of care for telehealth msk service provision"

"Good"

"Great research priority"

"Health App"

"helpful, but not really a priority"

"Homogeneity of terminology is important to guarantee that all the stakeholders are talking about the same things"

"I agree although terms will continue to evolve as technologies emerge so I'm not sure how up to date the terminology could be"

"I guess this work falls within this category. I also believe that there are frameworks available, but no agreement on which ones to use. "

"Important to standardise terms in research and across stakeholders so we know what everyone is talking about/ talking the same thing or not"

"In spite of my last statement about disliking business models, I do think that standardization of terms is important. "

"indeed, we have to consider that the usage of the right terms and wording is important for the process of dissemination"

"It's important, but I don't think it's resolved by research"

"Language evolves as people use it. Stardardizing leads to strange terminology that people don't understand and use wrong anyway. "

"Maybe but the field of DTx is much bigger than pain and there is a danger of creating a standard vocabulary that does not communicate outside of pain"

"NA"

"Needed"

"No"

"No"

"No"

"No"

"no"

"no"

"no"

"no"

"No suggestion"

"Not a priority"

"proper training"

"Standardization is key - from an outcome comparison and a safety perspective"

"The use of a wide range of terminology can be confusing for patients, clinicians, and also researchers to navigate. "

"This is important! "

"This is why this project is really important"

too much confusion around terms and definitions, too many terms"

"Universality"

"we need to be on yje same page liguistically to know we are all talking about the same thing. we don'rt want to have to go back and say well that might not have been the same thing"

"We need to use the same terms"

"We need too much information before getting to this step"

***Would you like to comment on the research priority of _"_The role of organizations and advisory boards in supporting the use of evidence-based telehealth in musculoskeletal conditions_"_***

"Again, too specific to particular conditions to be useful"

"Already commented"

"At least at the beginning this is going to be important as services change"

"Boards and organizations tend to sometimes be forgotten in engaging support from the top levels"

"Good"

"Government of private sector should take it as priority basis"

"I am an entrepreneurship with a research formation and I like to think that support evidence-based practice is the safest way to build prosper and secure business models in healthcare. However, common organizations, healthtechs and venture capitalists look for and concern about "proven business concepts" and sustainable business models. Therefore, in my opinion the quickest way to assist organizations and advisory boards in supporting the use of evidence-based telehealth in musculoskeletal conditions is to demonstrate that telehealth is an accessible solution with cost-effectiveness and can be used to build sustainable and profitable business models. "

"Important"

"NA"

"Não"

"No"

"No"

"No"

"No"

"no"

"no"

"no"

"no"

"No comment"

"No suggestion"

"Not a priority"

"Regulatory affairs"

"This is very important but I am not sure it is a 'research' priority"

"While i think they play an important role, once integrated into curricula for example or into routine practice they may play less of a role"

"Will drive uptake"

"yes"

***Would you like to comment on the research priority of _"_New developments and advances in telehealth communication and information technologies considering predictive models and the use of artificial intelligence_"_***

"AI/VR and msk teleheath"

"Already many ongoing projects"

"Artificial intelligence, if appropriately used, may facilitate effective diagnosis, prognosis, and treatment delivery. More research needs to be done in this field"

"Building level of evidence"

"Despite the technology already exist, the cultural adaptation and the response to specific need of the population is still a challenge. "

"extremely important for the process"

"Good"

"I don't understand how this is a research activity"

"Important"

"Important on a population / public health level. "

"Maybe. There is a lot of hype. Those who are invested algorithm development are not technology specific (e.g., IESO) "

"NA"

"Não"

"New development is always welcome"

"No"

"No"

"No"

"No"

"no"

"no"

"no"

"no"

"No comment"

"No suggestion"

"No suggestions"

"Pilot work in managing acute pain after surgery is compelling"

"Recent advances with future perspective will promote new models of health"

"The future is here! "

"This has the potential to be innovative and future thinking. "

"This is certainly a paramount topic of research if, and only if, the previous research priorities were proven. In order to advance we must first prove that the solution works in every single relevant aspect of it. Otherwise, we will probably spend research and human resources in research topics that are not suitable with the today actual needs for implementation. "

"This will become increasingly important"

"understudied field within physiotherapy, therefor important"

"Very important"

"Very important topic. "

"Would be nice for the future. Too early now I guess. "

"yes artificial intelligence"

***Would you like to comment on the research priority of _"_Integration of telehealth devices with electronic health records and cloud databases_"_***

"A step in the right direction but can be left for later exploration"

"Again, this seems to be an area for industry to find best solutions. "

"can't comment on this, can't oversee"

"digital skills to read data"

"Good"

"Good"

"How to ensure data protection and avoid data-grabbing by tech monopolies seems of much larger importance to me. Currently, any acquisition of a promising e-health start-up by a large tech company, or cooperation between public services and private enterprise, hold the danger of data being misused. "

"I struggle with this. I want very badly for cloud databases to be a thing for patients, but so far in the US no systems talk to each other. "

"I think this research priority is too specific and may be merged into other priorities related with policy making, privacy, business model, implementation model, etc. "

"important topic, but not for research"

"Integration among all devices is important"

"Integration is essential to have more accessiblily of data"

"Integration will enable improved monitoring of telehealth uptake and add credibility to telehealth care as a useful alternative approach to face-to-face care. "

"Is this integration of 'devices' or 'information from telehealth encounters' to electronic health records? "

"it will be easier for research priorities already set"

"Maybe it´s important to study the best data protection system"

"NA"

"Não"

"No"

"No"

"No"

"No"

"no"

"no"

"no"

"no"

"No comment"

"No suggestion"

"proper training of healthcares"

"Safety of electronic health and cloud records"

"The future of the telehealth business is here"

"The more we can integrate these aspects the better for all - and most of all for the patient"

"This is a huge issue - our trust has an integrated system but it cannot send out patient information. Also we are not deemed a priority for being included in this and so have utilised an external provider to collect data and share patient information. We then need to upload PROMS data into the electronic patient record. "

"this is crucial to improve acceptance of telehealth among healthcare professionals"

"This is the unrealised part of remote healthcare, and undermines its efficacy. "

"This is urgent in the sense that it can be embedded with regular patient care without massive research investments/funding. "

"This will also vary significantly between health systems and regions around the globe

"Understand the need for continuity, but wonder if this is a general priority or only for a subset of telehealth applications"

"Very important"

"Very important to link telehealth to existing ressources like EHR and storage. Data security issues can be complicated and need to be resolved"

***Would you like to comment on the research priority of _"_Development of algorithms and analytical approaches for predictive models, personalized, and customized analytics, and devices to improve assessment and management of musculoskeletal conditions_"_***

"A quality research interest"

"Analytical approaches should modernized"

"as before"

"As per previous answer"

"Clinical judgement is still going to rule the day"

"Good"

"I don't understand how that is a research priority: it is a major research activity at present"

"I worry about taking the individualization out of caring for patients. "

"it needs too much data we don't have now"

"May be merged with the model re: artificial intelligence. "

"Must for optimization and quantification of musculoskeletal conditions"

"NA"

"Não"

"No"

"No"

"No"

"No"

"no"

"no"

"no"

"no"

"No comment"

"No comment"

"Predictive modelling/AI and telehealth MSK healthcare provision"

"proper training"

"related to the other AI research priority"

"struggle to understand the wording"

"Thats the future!!! "

"Very important"

"we are moving more to personal medicine perhaps adding personal to the priority"

***Would you like to add any missing research priorities not mentioned before?***

"acceptability to patients and clinicians in different setting, regions of economic development and consideration of equity for people with disability (vision, hearing, speech, cognitive or fine motor/dexterity impairments). I sense we are missing the important health equity considerations here. "

"as above: data protection / user rights / risk of harm through corporate data misuse

"Barriers to implementing Telehealth"

"Good"

"Good"

"I think these are enough"

"In a product building perspective, after identifying the main problems of a given population, developing a solution and verifing the problem-solution fit, a good solution would need to achieve the market (sustainable business models) and channel-fits (what are the channels most effective to sell/delivery the solution to clients?). Therefore, for implementing Telehealth solutions in real world scenarios, it would be worth full to research the channels though it eHealth interventions and solutions could be delivered/reach potential users. "

"International use of telehealth msk service provision, is it safe? "

"It was quite extensive! "

"maybe a comparision between teleheath and face-to-face"

"Não"

"No"

"No"

"No"

"no"

"no"

"No"

"No"

"No"

"No"

"no"

"no"

"no"

"No"

"No"

"no"

"No"

"No - great job in identifying major issues at individual, clinician and system levels"

"No thanks"

"no, my list is complete"

"nothing to add"

"Patient experience"

"Perhaps included in some of the mentioned priorities, but examining the perceptions and opinions of patients with regards to telehealth. What are their impressions, preferences, opinions, barriers to choose telehealth over in person visits? "

"research on how to measure user engagement and utilization of the digital components. "

"Research on models that works with Allied Health Professional versus a physician. "

"Scope of conditions and individual patient characteristics that are suitable for different modes of Telehealth (i.e. phone, video etc) "

"Security of personal information. "

"Sounds like you’ve got it covered, thank you! "

"specific research on demographics that might prove more difficult because perhaps it will be much more difficult for certain ages or disease processesin"

"The ethical rules and patient privacy are also very important in this regard. "

"User satisfaction in patients"

"Validity of initial assessment"

"We use the term 'blended care', that one is missing in your overview"

"What should be the frequency of these Tele-sessions and when to use them"

"XR immersion technologies"
