# Supplementary material for: One step at a time. Shaping consensus on research priorities and terminology in telehealth in musculoskeletal pain: an international modified e-Delphi study
Source: BMC Musculoskelet Disord. 2023 Oct 3;24:783. doi: 10.1186/s12891-023-06866-0 (PMC10546725; doi:10.1186/s12891-023-06866-0)
Supplement: Supplementary file 11 — Additional file 11: Supplementary file 11. Feedback with all the raw results of the second round. [file 12891_2023_6866_MOESM11_ESM.docx]

**Supplementary file 11. Feedback with all the raw results of the second round.**

**Which country do you currently reside in?**

**What is your main Stakeholder Group?**

**What is your gender?**

**Highest level of education**

**Panel members - Researchers**

**What is your area of research?**

**What is the main treatment approach that you research in telehealth?**

**What telehealth modality(s) have you been researching?**

**Panel members - Consumer representative**

**Which of the musculoskeletal conditions did you receive telehealth for?**

**Which modality(s) of telehealth have you experienced?**

**Panel members - Developer and industry partner**

**What is your area of work?**

**What is the main treatment approach that you work in telehealth?**

**What telehealth modality(s) have you been working with?**

**Standard terminologies for telehealth in musculoskeletal pain research**

**Do you support the use of the term ___ as standard terminology?**

**Standard terminologies for telehealth in musculoskeletal pain research**

**Do you support the use of the term ___ as standard terminology?**

**Standard terminologies for telehealth in musculoskeletal pain research**

**Do you support the use of the term ___ as standard terminology?**

**Standard terminologies for telehealth in musculoskeletal pain research**

**Do you support the use of the term ___ as standard terminology?**

| **Second Round Summary** | | |
| --- | --- | --- |
| Overall summary to support the use of the term as standard telehealth terminology in musculoskeletal pain suggested by the panel members. | | |
| Nº | Telehealth terms | Round 2 |
|  |  | Strong agreement (> 80 %) |
| 1 | Digital health | 84.21% |
| 2 | Telehealth | 81.95% |
| - | - | - |
|  |  | Moderate agreement (70% - 80%) |
| 1 | Mobile health app (mHealth) | 73.68% |
| 2 | Digital health intervention | 73.68% |
| 3 | Online consultations | 71.43% |
| 4 | Telemedicine | 70.68% |
| 5 | Teleconsultation | 70.68% |
| - | - | - |
|  |  | Low agreement (50% - 70%) |
| 1 | Video consultations | 64.66% |
| 2 | Electronic health (eHealth) | 63.91% |
| 3 | eHealth Intervention | 63.91% |
| 4 | Telerehabilitation | 63.16% |
| 5 | Digital rehabilitation | 63.16% |
| 6 | Online healthcare | 62.41% |
| 7 | Remote monitoring | 56.39% |
| 8 | Remote home-based exercise program | 54.89% |
| 9 | Online pain management | 54.14% |
| 10 | Videoconferencing | 50.38% |
| - | - | - |
|  |  | No agreement (< 50%) |
| 1 | Self Monitoring | 49.62% |
| 2 | Virtual consultations | 48.90% |
| 3 | Virtual appointment | 47.4% |
| 4 | Digital care program | 47.37% |
| 5 | Digital Health Application (DiHA) | 46.62% |
| 6 | Remote self-management support | 44.36% |
| 7 | Tele-assessment | 43.61% |
| 8 | Telephysiotherapy | 42.86% |
| 9 | Video assessments and treatment | 42.11% |
| 10 | Virtual clinic | 42.10% |
| 11 | Telephone assessments and treatment | 41.35% |
| 12 | Virtual exercise prescription | 39.90% |
| 13 | Telephone intervention | 39.85% |
| 14 | Technology-based intervention | 39.10% |
| 15 | Virtual Physiotherapy | 37.60% |
| 16 | Remotely delivered treatment | 36.84% |
| 17 | Virtual reality, Augmented reality & Extended reality (VR, AR & XR) | 36.80% |
| 18 | Digital tool | 30.08% |
| 19 | Telepractice | 27.82% |
| 20 | Virtual reality immersive/ non immersive | 27.10% |
| 21 | Virtual reality asynchronous/synchronous | 27.10% |
| 22 | Blended care | 24.06% |
| 23 | Virtual physical assessment and provisional tele diagonosis | 21.00% |
| 24 | Distance physiotherapy | 19.55% |
| 25 | Remote presence | 11.28% |
| 26 | Audio-simultaneous rehabilitation | 5.26% |
| - | - | - |
| **New or suggested telehealth terms for round 2** | | |
| 1 | Asynchronous Care Delivery Platform |  |
| 2 | Digital health rehabilitation |  |
|  |  |  |
| **Telehealth terms suggested and repeated with the previous list** | | |
| 1 | Tele PT (most likely from Telephysiotherapy) |  |
| 2 | Telephysiotherapy |  |
| 3 | Internet based interventions | - |
| 4 | Virtual care |  |
|  |  |  |
|  |  |  |
| **Telehealth terms re-edited or modified** | | |
| 1 | From mobile health app to mobile health | - |
|  |  |  |
| **Telehealth terms included in Round 3** | | |
| 1 | Mobile health (mHealth) | 73.68% |
| 2 | Digital health intervention | 73.68% |
| 3 | Online consultations | 71.43% |
| 4 | Telemedicine | 70.68% |
| 5 | Teleconsultation | 70.68% |
| 6 | Video consultations | 64.66% |
| 7 | Electronic health (eHealth) | 63.91% |
| 8 | eHealth Intervention | 63.91% |
| 9 | Telerehabilitation | 63.16% |
| 10 | Digital rehabilitation | 63.16% |
| 11 | Online healthcare | 62.41% |
| 12 | Remote monitoring | 56.39% |
| 13 | Remote home-based exercise program | 54.89% |
| 14 | Online pain management | 54.14% |
| 15 | Videoconferencing | 50.38% |
| 16 | Asynchronous Care Delivery Platform | - |
| 17 | Digital health rehabilitation | - |
| **Telehealth terms automatically included in Delphi's final list (consideration of the panel members' suggestions after committee agreement).** | | |
| 1 | Digital health | 84.21% |
| 2 | Telehealth | 81.95% |
|  |  |  |
|  |  |  |
|  |  |  |

**Overall suggestions, re-wording, and comments for any terminology provided by the panel members.**

"No"

"No"

"No"

"Tele PT"

"Telephysiotherapy"

"No"

"Asynchronous Care Delivery Platform"

"não"

"Clarification on Online Pain Management - Is this synchronous or asynchronous?"

"No"

"Video consultation rather than virtual"

"no"

"no"

"no"

"no"

"no"

"no"

"no"

"no"

"Supported self-management would be good, but it doesn't exist as an option to my knowledge?"

"no"

"no"

"As per our previous page. Keep it simple and focussed"

"From mobile health app to mobile health (indicating it can be done from anywhere, rather than that it is just app based. Web applications can also be accessed via mobile phone)"

"no"

"no"

"no"

"Be carefull with the use of 'tele' as a prefix, because it is not clear if it refers to 'tele as in distance' or 'tele as in abbreviation of Telephone'."

"Maybe, but I am not sure."

"nothing to add"

"no"

"no"

"no"

"no"

"no"

"Some of the terminologies have similarities"

"no"

"no"

"My opinion is in the future we should eliminate the word "tele-" "virtual-" because a teleconsultation is an clinical care carried out using technology, and must follow the same laws than face-to-face consultation"

"no"

"Virtual care"

"Digital health rehabilitation"

"no"

"I think the term 'Virtual physical assessment and provisional tele diagnosis' should be two separate terms. A provisional telediagnosis does not necessarily have to be limited to physical assessments."

"no"

"Nonee"

"Consider more options with prefix 'video' so that it is distinguished from 'virtual'. It seems that some definitions of 'virtual' include telephone and video; and in my opinion, they are very different and each have advantages and disadvantages."

"no"

"Internet based interventions"

"no"

"Anything that's precisely describing what goes on (more words are needed)"

"None, There are some terms not related to telehealth at all. I would avoid endorsement of terms that are related to "virtual" mainly because it means "not real" for many.".

**Research priorities for telehealth in musculoskeletal pain research**

**How much do you agree this should be a research priority?**

**Research priorities for telehealth in musculoskeletal pain research**

**How much do you agree this should be a research priority?**

**Research priorities for telehealth in musculoskeletal pain research**

**How important is this research priority?**

**Research priorities for telehealth in musculoskeletal pain research**

**How much do you agree this should be a research priority?**

**Research priorities for telehealth in musculoskeletal pain research**

**How much do you agree this should be a research priority?**

**Research priorities for telehealth in musculoskeletal pain research**

**How important is this research priority?**

**Research priorities for telehealth in musculoskeletal pain research**

**How important is this research priority?**

| **Second Round Summary** | | | |
| --- | --- | --- | --- |
| Overall summary for telehealth research priorities in musculoskeletal pain suggested by panel members. | | | |
| Nº | Telehealth research priorities | Round 2 | |
|  |  | Strong agreement  (>80%) | Level of importance (Mean/SD) |
| 1 | Identification of patient characteristics that affect response to treatments delivered by telehealth | 91% | 8.0 (1,7) |
| 2 | Qualitative telehealth research to determine perceptions, barriers, and enablers in the management of musculoskeletal conditions | 87.2% | 8.3 (1,9) |
| 3 | Research that examines the specific contribution of communication and information technology and digital skills to the effectiveness of telehealth treatments in musculoskeletal conditions | 83.5% | 7.5 (1,8) |
| 4 | Standardization of telehealth-related terms and the development of frameworks and guidelines for musculoskeletal telehealth practice | 82% | 7.7 (2) |
| - | - | - | - |
|  |  | Moderate agreement  (70% - 80%) | Level of importance  (Mean/SD) |
| 1 | Investigation of patient-related safety risks and adverse events during telehealth encounters for musculoskeletal conditions | 78.2% | 7.3 (2.3) |
| 2 | Research on reliability and validity testing for diagnostic tests suitable for telehealth (compared to in-person testing) in individuals with musculoskeletal conditions | 78.2% | 7.7 (2) |
| 3 | Integration of telehealth devices with electronic health records and cloud databases | 77.4% | 7.7 (2.2) |
| 4 | Identification of mediators contributing to the effects of telehealth-delivered treatments | 77.4% | 7.7 (2) |
| 5 | Research on suitable patient-oriented research outcome measures for telehealth in individuals with musculoskeletal conditions | 71.4% | 7.3 (2.3) |
| - | - | - | - |
|  |  | Low agreement  (50% - 70%) | Level of importance  (Mean/SD) |
| 1 | Development of algorithms and analytical approaches for predictive models, personalized, and customized analytics, and devices to improve assessment and management of musculoskeletal conditions | 70% | 7.1 (2.3) |
| 2 | Translation, dissemination and communication developed with all parties involved | 69.9% | 7.1 (2.5) |
| 3 | The role of organizations and advisory boards in supporting the use of evidence-based telehealth in musculoskeletal conditions | 69.9% | 6.9 (2.4) |
| 4 | New developments and advances in telehealth communication and information technologies considering predictive models and the use of artificial intelligence | 69.2% | 7.1 (2.4) |
| 5 | Identify, explore, and implement the most suitable business models to support the delivery of telehealth treatment for individuals with musculoskeletal conditions | 67.7% | 6.9 (2.3) |
| 6 | Identification of clinician characteristics and beliefs that affect response | 62.4% | 6,7 (2.5) |
| - | - | - | - |
| **Research priorities included for round 3** | | | |
| 1 | *Investigation of adverse events and patient safety during telehealth encounters for musculoskeletal conditions* | 78.2% | 7.3 (2.3) |
| 2 | Research on reliability and validity of clinical assessment and diagnostic tests administered via telehealth (compared to in-person testing) in individuals with musculoskeletal conditions | 78.2% | 7.7 (2) |
| 3 | Identification of mediators contributing to the effects of telehealth-delivered treatments | 77.4% | 7.7 (2) |
| 4 | Research on suitable patient-oriented research outcome measures for telehealth in individuals with musculoskeletal conditions | 71.4% | 7.3 (2.3) |
| 5 | Translation, dissemination and communication developed with all parties involved | 69.9% | 7.1 (2.5) |
| 6 | The role of organizations and advisory boards in supporting the use of evidence-based telehealth in musculoskeletal conditions | 69.9% | 6.9 (2.4) |
| 7 | New developments and advances in telehealth communication and information technologies considering predictive models and the use of artificial intelligence | 69.2% | 7.1 (2.4) |
| 8 | Development and testing of innovative business models to support the delivery of telehealth in musculoskeletal conditions. | 67.7% | 6.9 (2.3) |
| 9 | Identification of clinician (health professional) characteristics and beliefs that affect response to management via telehealth | 62.4% | 6.7 (2.5) |
| 10 | Data science initiative to support the use of telehealth in musculoskeletal conditions* | - | - |
|  |  |  |  |
| **New or suggested research priorities for round 3** | | | |
| 1 | Research and development of strategies for using information and communication technology to facilitate access to individuals with musculoskeletal conditions in remote or rural regions | - | - |
| **Research priorities automatically included in Delphi's final list (consideration of the panel members' suggestions after committee agreement).** | | | |
| 1 | Research about how to implement telehealth services at the user, clinician and health system level with patient/consumer involvement | 91.88% | 8.4 (1.8) |
| 2 | Identification of patient characteristics that affect response to treatments delivered by telehealth | 91% | 8.0 (1.7) |
| 3 | Effectiveness of treatment approaches delivered via telehealth in the management of musculoskeletal conditions | 89.38% | 8.5 (1.7) |
| 4 | Equity research on interventions to improve access, treatment or clinical outcomes to telehealth services for disadvantaged or historically underserved populations with musculoskeletal conditions | 88.13% | 8.3 (1.9) |
| 5 | The cost-effectiveness of telehealth treatments for musculoskeletal conditions considering different settings and income levels | 88.13% | 8 (2.2) |
| 6 | Qualitative telehealth research involving all end-users to determine perceptions, barriers, and enablers in the management of musculoskeletal conditions | 87.2% | 8.3 (1.9) |
| 7 | Design and evaluation of curricula to train students and health care practitioners in the provision of telehealth that conforms to core capability frameworks | 83.75% | 7.7 (1.9) |
| 8 | Research that examines the specific contribution of communication and information technology and digital skills to the effectiveness of telehealth treatments in musculoskeletal conditions | 83.5% | 7.5 (1.8) |
| 9 | Standardization of telehealth-related terms and the development of frameworks and guidelines for musculoskeletal telehealth practice | 82% | 7.7 (2) |
| 10 | Research on health literacy, eHealth literacy, technology literacy and identifying relevant factors for patients with musculoskeletal conditions engaging in telehealth (eg, barriers and facilitators) | 81.88% | 7.8 (2.1) |
|  |  |  |  |

**Comments and suggestions for research priorities provided by the panel members**

***Would you like to comment on the research priority of _"Qualitative telehealth research to determine perceptions, barriers, and enablers in the management of musculoskeletal conditions"_***

"Seems correct"

"This priority ise appropriate for me"

"Need to consider clinician, patient and health service perspectives"

"Barriers, perception, stake holders"

"no"

"não"

"Virtual Care can be an enabler of efficient and effective health care services however we need to understand both patient and provider perceptions/barriers and enables to optimize care delivery"

"I hear a lot of assumptions about the pros and cons of telehealth, and how well it does or doesn't work. I have seen some people have improved access, others who did not doo so well. We need research to guide us - is in person really better? when and for whom?"

"I think it is extremely important that qualitative studies be carried out on this topic"

"No"

"nil to add"

"No"

"Qualitative tele health research is modern concept for promoting rehabilitation for outreach to masses with aim to provide evidence based medicine"

"No"

"No"

"No"

"If we do not understand barriers and enablers we will not move forward appropriately with solutions to try - potentially wasting time and money and patient and healthcare worker satisfaction."

"Patient partners/consumers should be involved"

"People cannot always access in person therapy yet are uncertain about telehealth and so we need to see what can make it easier as well as what makes it hard for them to access it so we can have more people seeking and getting treatment for chronic pain."

"No"

"No"

"No"

"stakeholder engagement is key to moving this into practice sustainably"

"No"

"no comment"

"Highly relevant because we get to know the views of the stakeholders for real-world evaluations."

"No"

more than effectiveness is needed, also patient engagement, feasibility, acceptability, satisfaction with new treatment approaches"

"There has been quite a bit of qual work done in this space now"

"very important"

"trials tend to focus on motivated and educated samples which is biased"

"This is a important topic to discuss before the implementation of the Telehealth."

"has been studies already, now it's time to move to solutions"

"no suggested changes"

"While the area is relevant and high priority, I have only rated its priority as ~7 as there are more urgent questions to address prior to determining the perceptions/ barriers/ enables of telehealth in musculoskeletal conditions - to name a few, its efficacy, safety, and equity to various populations. "

"Nothing much"

"Qualitative telehealth perceptions determine the possibility that the patients use this technology as a common way of treatment. To know what are the barriers that the users or the professionals have around the use of the digital and remote ways of e-health tell us if the e-health could be or not be implemented as a regular way of health service"

'Telehealth' would need definition. Does this refer to telephone, for example?"

"No"

"Assume will cover all stakeholders involved"

"This is IMO maybe the most important priority"

"No"

"The descriptors are too vague to get a good impression of what they encompass"

"Barriers and enablers are much more than just at patient level, but investigation rarely addresses this. The findings rarely generalise across patient populations or practice settings."

"***Would you like to comment on the research priority of _"Identification of patient characteristics (moderators) that affects the response to treatments delivered by telehealth"_***

"Seems correct"

"This priority is appropriate"

"Patient characteristics are important because many times we may depends inpatient reported outcomes measures"

"não

"It is very relevant because it would facilitate the identification of potential responders, allowing an initial screening when deciding whether to choose a face-to-face or online modality"

"It may also be their presenting condition. You can assess low back pain virtually and determine need for escalation of care quite easily however another body part may require a hands on assessment. It may not be a patient characteristic but the medical presentation. Also a follow up appointment may be more appropriate virtually versus a new assessment with a new health care provider. Also, provider confidence with virtual care delivery is another component. Patient characteristics are one component of response to treatment."

"I am sure you will come onto it but feel the clinicians approach potentially is the most important. Other than if clear comprehension issues patient wise"

"stratification to identify patients that will respond its important"

"Important in order to be able to stratify patients accordingly"

"No"

"No"

"No"

"Tele health can be subsidiary to contact based management to have greater impact"

"No"

" Identification of all the factors that can hinder patients from accessing healthcare facility is must"

" This is relevant because it will help inform if the treatments are worthwhile/successful which is surely a priority as it impacts cost effectiveness, and also understand what treatment path to take."

"No"

"No"

" Very important to success of telehealth/rehab"

" I think this is important but I think we need enough people of different demographics participating first to get a true idea, however we need good outcomes to get more people to participate so thus it's importance"

" no"

"no"

"Very important, as this issue is especially relevant in a very heterogenous population with regard to i.e. sociodemographics, technological experience, health competence etc."

"Very good initiatives"

"This should be combined with qualitative to understand the nuances"

"No"

"no comment"

"No comment"

"No"

"I think this is a very high priority for the field, because I think we need to be cautious about concluding this treatment works, or that treatment doesn't work. Looking at moderators helps us to understand in a more sophisticated what what works for whom, and in particular who does and does not benefit from digital models of care"

"This is important"

"this is relevant to quantifies the extent to which variables may participate in the change of an effect size"

"what works for whom is important in all areas"

"Similar to the first research priority mentioned, patient characteristics to better identify and deliver appropriate (tele)health treatments should be done once its efficacy, safety, and equity are addressed. Once the delivery of telehealth becomes feasible from these perspectives, we may then move onto building a protocol or evidence-informed decision-making on which patient characteristics to observe for when taking telehealth into consideration."

"Nothing new"

"It is important to characterize what kind of patients better accepted this type of services"

"Same comment as above regarding definition of 'telehealth'."

"no"

"no"

"Again, patients aren't homogeneous across countries and health systems. Moderators on the patient side occur in relation to specific interventions, settings, health culture etc."

***Would you like to comment on the research priority of _"Investigation of patient-related safety risks and adverse events during telehealth encounters for musculoskeletal conditions"_***

"Seems correct"

"This priority is appropriate for me"

"Research on patient safety and adverse effect of needs to be measured before recommend as treatment"

"No"

"não"

"Risk of AEs via telehealth is presumed to be low, as long as proper screening for red flags and risk factors is done"

"This becomes quite important once you've had your first "code blue" virtually and you do not have an established process for risk mitigation. There should be safe guards put in place to minimize risk, communication plan of emergency contacts and an action plan if things do need to be escalated."

"We need to make sure that we are still identifying people who need immediate medical care, and that telehealth is at least as safe as in person care."

"rare Risk situations"

"No"

"nil"

"No"

"Investigation should be based upon logical as well as feasibility"

"no"

"I don't feel it's too important because I'd like to think that risk assessments would have been carried out before agreement on the delivery of the various types of Telehealth that would be provided."

"No"

"No"

"Adverse events are underreported in general so this is an important priority"

"Because people living with pain often have psychosocial factors affecting their pain there can be risks associated with virtual appointments where the patient might become overwhelmed and not know where to turn in person, or the clinician might not pick up on this and not follow up closely enough so I think we need to be cognizant of "do no harm"

"fail risk"

"It is important to define which pathologies are suitable for telehealth guidance."

"No"

"Telehealth has great potential to counteract an underreporting of adverse events in non-pharmacological conservative interventions such as exercise"

"No"

"Important to embed in other investigations"

"No

"no comments"

"Patient-related safety risks are usually looked into at physical clinics and should be given high considerations for telehealth encounters in wherever space the patients/clients are"

"no"

"Fair amount of evidence on this already"

"It is important to consider possible risks and adverse events in new health technologies"

"This would be part of the other research areas"

"Assume this to be relatively safe and many risks to be transferable; apart from some specialist (usually non-msk) populations"

"Efficacy, safety, and equity are key before considering the further breakdown of telehealth such as moderators, clinician characteristics, organizational roles, etc."

"No"

"The physiotherapists have to know how to control o minimize the risks related with the e-health modalities"

"No "

"I see the interventions tat can be delivered using telehealth as generally safe, so not a high priority"

"No"

"Too little attention is paid to this. We should always be able to describe likely benefits and possible harms to patients before they decide on a treatment."

***Would you like to comment on the research priority of _"Research on suitable patient-oriented research outcome measures for telehealth in individuals with musculoskeletal conditions"_***

"Seems correct"

"This priority is appropriate for me."

"Mandatory for physiotherapy. Most of the Pt treatment outcomes can be quantified by patient reported outcome measurement."

"No"

"não "

"Unclear why outcome measures should be any different for telehealth."

"Es importante pero difícil debido al carácter dinámico de la práctica digital"

"Validity and Reliability of the Virtual Physical exam needs to be explored. Also, the assessment of subjective outcome measures in a virtual platform. ie. Are pts more comfortable answering questions virtually versus onsite versus online."

"we already have to many PROMS"

"No"

"nil"

"We already have it."

"Essential for generating data and database has scope to be applied on wide areas"

"No"

"I'm not sure I actually understand this, so cannot offer re-wording or an accurate level of important, sorry. "

"No"

"No"

"While outcome measures are necessary we must not forget that process and structure measures are needed to support success in outcome measures and that balancing measures are needed to ensure something else does not suffer."

"Patient partners/consumers should be involved in establishing the PROMs"

"If we do not have reliable and valid measures we cannot know how well or poorly the intervention really is doing. We also need to know it works before we increase the volume and diversity of the clientelle"

"PROMS and PREMS"

"No"

"From my opinion, the understanding of measurement properties of outcome measures (e.g., digital formats) designed to be used via telehealth is the most relevant focus of this research topic"

"Requirements are high"

"Need to make sure we are capturing the key drivers"

"No"

"no comment"

"It is all about the most important stakeholders - the patient. Patient-centred, reported outcome measures are key."

"No "

"To identify core outcomes is also important for conducting cost-effectiveness analysis in the area"

"not so specific for telehealth"

"Likely transferable from in-person health research"

"I agree that outcomes may differ for telehealth-based interventions compared to traditional face-to-face interventions, and this should explored."

"While this field is important, it also seems feasible to merge this with other mentioned research priorities such as the 'research on diagnostic tests suitable for telehealth...', as what is considered a 'suitable' test may also need to be patient-oriented."

"No "

"The inclusion of assessment tools such as validated questionnaires is common in telehealth. However, it is necessary to validate the instrument itself in digital format, as the instrument's format often changes. Psychometric properties may change."

"Outcomes of the telehealth services on the conditions are something that allowed to determine the value of these services."

"Presumably, self-report outcome measures as per body part would be similar to those used in-person. If the question is addressing outcome measures to assess telehealth interventions, I think this a needed research priority."

"No."

"Involvement of patients is crucial"

"No"

"Not specific to eHealth (goes for all health care)"

"There are opportunities to use more than PROMs here and we should investigate them."

***Would you like to comment on the research priority of _"Research on reliability and validity testing for diagnostic tests suitable for telehealth (compared to in-person testing) in individuals with musculoskeletal conditions"_***

"Seems correct"

"This priority is appropriate for me."

"Important"

"Não "

"We know that orthopedic tests are limited when compared to imaging tests and that special tests are not so special. Comparing one with the other would give the false sensation that those performed in the office are more reliable than they are in reality."

"Diagnostic accuracy of the physical exam. ie. is Passive SLR in supine lying as effective as Active SLR in sitting to rule in/out leg dominant pain from discogenic radiculopathy?"

"I would LOVE to see this research be done. I know my bias, and that a lot of people disagree. Considering pain is often part of the diagnosis, and we are learning that many "hands on" interventions aren't more effective than other interventions - can we accurately diagnose and treat people remotely?"

"Useful to highlight use of or not diagnostic tests though very medical model biased"

"Effective education is extremely important to support the uptake of Telehealth"

"Encourage patient partners/consumers to be involved"

"physical exam its a issue in telehealth"

"No "

"nil"

"No"

"Strict measures to avoid falsification and research bias with more control is essential"

"No"

"Diagnostic tests and Diagnosis is core for the treatment. So appropriate selection of tests is a necessity"

"No "

"No"

"this goes with the last priority It is a must!"

"No"

"Since we do not have yet sufficient produced evidence in general I think that all the topics so far are very important and I can not help to establish a priority from one over the others."

"No"

"This is going to be needed to support scaling"

"No"

"no comment"

"No"

"Tests are not standardized without evidence of psychometric properties of which reliability and validity are most important."

"No"

"tests should always be reliable and valid"

"We know that most orthopaedic tests are not specific so why would telehealth be any different? I would suggest this be framed around the ability to undertake an objective clinical assessment, rather than specific diagnostic tests"

"With a preference for blended care, I believe the diagnostics should be preferably in-person"

"This would be a 'phase II' of the previous priority on identifying suitable diagnostic tests for telehealth. Once would have to identify the tests first (preferably patient-oriented as mentioned) then proceed to the reliability and validity testing - all seem to fall under one umbrella."

"No "

"Very important. It is common to observe how telemedicine tools advertise diagnostic methods, without having been previously validated. We need to control this situation."

"Objective measures that allowed the PT diagnosis and prescribe the physical exercise and modalities are a necessity to demostrated the impact of these services and to assure the security of the patients"

"I think this is important as it will help guide clinicians when assessment by videoconference is the right choice and when in-person assessment is required. I also think there needs to be research on aspects of the history that would guide one to a videoconference assessment versus an in-person assessment."

"No"

"Not that we have great reliability and validity for tests done person-to-person......"

"no"

"I fail to see the relevance besides from in truly remote regions (e.g., outback AUS)"

"Only to the extent that there are very few reliable diagnostic tests in msk conditions."

"These are prerequisites before we start using tests that we use in-person with patients before making them norms in telehealth and only identifying in a decade that these tests aren't reliable and valid in the telehealth settings"

***Would you like to comment on the research priority of _"Identification of clinician characteristics and beliefs that affect response"_***

"Seems correct"

"This priority is appropriate for me."

"Patient clinician relationship is an important for good treatment"

"No "

"Não"

"Clinicians acceptance, adoption and confidence in virtual care will have impacts on the patient. In addition, the clinicians admin staff who are booking the appointments and the available online support a patient has if they can't connect makes a difference"

"If a clinician perceives telehealth as poor value care that is what they in general will deliver"

"its already clear that the profile of a clinician that likes to work with telehealth its different..."

"No"

"correlation between clinician and patient beliefs on telehealth"

"Variability among individuals is key for heterogeneity of research data"

"No "

"Yes - it's not all about the patient, the clinician is just as important. Their characteristics and beliefs will play into the treatment they deliver, so I believe this to be vital."

"No

"No "

"There is not enough information provided to fully understand what will be researched. That affect response to what? Uptake of the method used to provide care?

"We already know that beliefs and attitudes of clinicians affect outcomes so yes if a clinician is in a clinic that provides telehealth but does not believe it to be effective , then this can seriously affect the outcome. We have worked so hard on changing beliefs and attitudes of clinicians about chronic pain. We need to work on this before it becomes a major player in the effectiveness of telehealth"

"no"

"no"

"Interesting topic, however I would not prioritize it, as the personal experience and knowledge on a specific type of intervention will always affect response"

"No "

"Not as important as patient perceptions"

"Identification of clinician characteristics, biases and beliefs that may impact telehealth outcomes

"No"

"No comment"

"No "

"not in first instance"

"Uptake of Telehealth services has been slow and there are many barriers that need to be better understood"

"without understanding service provider perspectives, intervention delivery success will be limited"

"Identifying clinician characteristics seems important but lower priority, as these are more for the sake of education once telehealth is launched. There will always be clinician characteristics that may affect response, but this is not specific to the field of musculoskeletal conditions."

"It is important that the clinician is involved in the technology adoption process, not just the patient. "

"No"

"It is relevant to define the competencies, experience, and expertise that the PT needed in order to have and optimal conditions"

"You may want to include the word 'attitudes' as well. "

"no"

"no"

"Not specific to eHealth"

"As above: clinician characteristics interact with intervention, setting, patient population etc. They are not fixed nor independent. "

***Would you like to comment on the research priority of _"Identification of mediators contributing to the effects of telehealth-delivered treatments"_***

"Seems correct"

"This priority is appropriate for me."

"May be important"

"No"

"Não"

"Can a therapeutic alliance be made virtually? Based on my clinical experiences it most definitely can. The patient introduces me to their family, pets, shows me their house etc. It has been better than I expected. however, patients who choose virtual may be more comfortable in their house and/or more comfortable with technology."

"No"

"nil"

"No

"Mediators contribute to effective outcome"

"no"

"no"

"no

"no"

"no"

"no"

"No comment"

"No

"No comment

"No"

"No comment"

"These mediators when understood can help to improve the effectiveness of tele-health interventions, reduce chronicity of pain and should be the concern of healthcare professionals"

"No "

"This is important because it will enable us to understand why some interventions are more effective than others, enabling us to iteratively develop more effective interventions for different populations - when combined with the moderator analyses."

"important, but again not the first priority"

"May be merged with the clinician characteristics that affect telehealth treatments, as similarly, it seems more for the purpose of education/ protocol-building once telehealth-delivered treatments' safety, efficacy, and equity are resolved/ addressed."

"No"

"Maybe if we analyse clinician´s and patient´s factors previously exposed, we might control those mediators."

"It is important to identify the extrinsic and intrinsic factors that could mediate the effects and the outcomes of the telehealth services"

"I think this is important but once we know more about telehealth delivered treatments."

"No "

"No"

"This allows more modelling of interactions between patient level, clinician level, and system level variables, if the population is large enough"

***Would you like to comment on the research priority of _"Research that examines the specific contribution of communication and information technology and digital skills to the effectiveness of telehealth treatments in musculoskeletal conditions"_***

"No comments"

"This priority is appropriate for me."

"Technology literacy may impact the treatment outcome in telehealth"

"No "

"não"

"Mix methods delivery - phone versus video, how much digital skills is required to access video care?"

"Technology is already a part of healthcare - it would be good to know more about the pros and cons of using it for MSK conditions."

"No"

"nil"

"No"

"Most important as it is variable factor in different areas, ethnicity ,states, country etc. And level of perception in individual capacity.also infrastructure for communication and information can contribute to variability"

"No "

"This is important from both a patient and clinician perspective - perhaps not everyone has got or can use the technology effectively to get the best out of Telehealth."

"No"

"no"

"I think this might be too many factors together. Perhaps I would look at them separately"

"no"

"no"

"Good work"

"Will be important to parcel out eventually but not the first step"

"no"

"Research that examines the impact of information technology and digital skills on the effectiveness of telehealth interventions for musculoskeletal conditions"

"No comments"

"No"

"While it is important to know what works and what doesn’t Im not sure our research designs and sample sizes can achieve this and technologies evolve quickly. So we need to be looking at the principles of what works and doesn’t instead of the specific technology."

"May be merged with "Effectiveness of treatment approaches delivered via telehealth in the management of musculoskeletal conditions", as both research would examine the efficacy of telehealth in various delivery methods - whether this be comparison between email, text, videoconferencing, etc."

"No"

"The development of the communication skills and ICT are relevant to the outcomes of the telehealth interventions"

"I think communication could be a separate study topic."

"No "

"Research that examines the specific contribution of communication and digital skills from who? Confusing…"

"This is all in vain if users do not have the necessary digital skills, we need to focus research on digital skills and health literacy in order to target TH to people who can benefit from it"

"No"

"This takes us closer to knowing when to use it, and when not. "

***Would you like to comment on the research priority of _"Translation, dissemination and communication developed with all parties involved"_***

"No comments"

"This priority is appropriate for me."

"Wider acceptance of the treatment is important in two ways. 1. Accessibility of successful treatment is everyone rights. 2. Telehealth may reduce the patient physio ratios"

"No"

"Digitalization is embedded in society, so healthcare systems have to respond at the same pace. Currently, they seem to be lagging behind and not taking advantage of the potential benefits of digitalization."

"Involvement of patient partners representing a full spectrum of diversity"

"Translation of evidence is very important and has been a struggle in other areas. I think getting the evidence of the efficacy of telehealth, etc would be a more important first step."

"No"

"No"

"not sure what you are meaning by this"

"No"

"Proper guided with well qualified and suitable translater and disseminater can richly contribute to research success"

"no"

"no"

"no"

"No matter what is developed - if it is not communicated in an appropriate and timely manner then it will not be known or used properly."

"patient partners/consumers should be one of the parties involved"

"we need to always ytake part in integrated KT in any research. I think it is imperative for all researchers"

"no"

"no"

"Need based"

"This is more of an embedded value than a program of inquiry."

"no "

"Knowledge translation approaches and impact for musculoskeletal telehealth"

"No comment"

"no "

"very important, but again rather step 2 after proving efficacy"

"Consumers should be involved in intervention design and dissemination plans. Ensuring that end-users are likely to use, or know how to access, telehealth interventions is vital."

"This field is important but I don't believe telehealth [to date] has been implemented enough to build such knowledge translation/ dissemination/ communication work. We need to focus on the foundation first before considering any further knowledge translation to broader parties."

"No"

"No comments"

"I think in the end, various terms will be used, as that is already the case. I think this points to the importance of clearly defining in any research what we mean by various terms, so that when one reads the literature, one can know if one is comparing similar modalities."

"No"

"No"

"Not specific to eHealth"

***Would you like to comment on the research priority of _"_Identify, explore, and implement the most suitable business models to support the delivery of telehealth treatment for individuals with musculoskeletal conditions_"_***

"Seems correct"

"This priority is appropriate for me."

"Important"

"no"

"Consultation based models delivered virtually - could be a business model for an individual practitioner and/or organization. Could also be intertwined with efficient models of care (RAC-LBP)

"i know interventions need to be sustainable, but I feel like we should find what's best practice, then investigate how to make it happen."

"No"

"nil"

"No"

"No comments"

"no"

"Telehealth setup requires a strong and suitable support and backup"

"No

"Important research question once first questions outlined earlier are answered"

"This is a challenging priority with there being so many differences in regions around the world as well as within regions"

"we need to explore this in order to have this type of intervention acceptable for third party payors to buy into for those who use third party payors"

"no"

"no"

"I think, this is a very important topic. However I am not sure whether this is more a political issue than a research topic. From my perspective, evidence from research should be translated to politics and other health system informers (see research topic "translation, dissemination and communication"). I would therefore argue that the research priority at this point should more be related to "implementation research" to evaluate the effectiveness and efficacy of telemedicine in real-world-scenarios rather than exploring the most suitable business modell. Maybe its just the wording "business modell" I am not getting along with."

"No"

"Need evidence first, and then payment is easier to make happen"

"No"

"none"

"No comment. "

"No"

"So, I'd be cautious about exploring business models before knowing what works, for whom, etc ... "

"This will differ from one health care system to another. "

"Very dependent on the (national) healthcare system context; But avoiding privatisation of most digital health solutions should be avoided. Similarly, the risk of using digital solutions to further-undermine in-person healthcare provision in favour of privatisation (in systems like the NHS) is real. "

"A large proportion of Australians do not have private health insurance. Statistics may vary across the globe, but are likely similar. Affordability is a key consideration. "

"I wonder if rather than business models we mean financial models for the delivery of telehealth. I'm not comfortable with the concept of healthcare as a business"

"No"

"The business models determine the possibility of insertion in the health systems

"This could be tricky as even within our country, Canada, we have different means of delivering physiotherapy - some is publicly-funded and some is privately funded (either through insurance or self-pay). "

"No"

Whether we like it or not, money determine what people (clinicians) do, so we need to find sustainable business models in this space

No

I think business developers do this fine without prioritising (limited) funds for research

Business models are different in every country, and even within countries. This benefits business, not patients.

***Would you like to comment on the research priority of _"Standardization of telehealth-related terms and the development of frameworks and guidelines for musculoskeletal telehealth practice"_***

"Seems correct"

"This priority is appropriate for me."

"no"

"Standard of practice for the assessment, diagnosis and management of MSK needs to be a focus for MSK practitioners."

"we need guidelines, but need research to include in the guidelines!"

"will be very important to develop guidelines in telehealth"

"No"

"nil"

"No"

"I think this is important only to researchers, we as clinicians still don´t know what a muscle contracture really is and you don´t see any PT desperate to find the right word to start treating patients. There are PT working with telehealth years ago and they keep practicing without any problem, even with a lack of clear concept consensus."

"More inquiry and use of scientific approaches help in standardized work

"No"

"No "

"The more we can standard terms the more helpful across the board. Also a framework is required to guide the work and how and when it is done."

"we have to talk the same language. There is nothing worse for me to read review articles where there is nothing conclusive because we are not talking about the same thing in trials. We need to start that way and disseminate this information early on in the process"

"No"

"No "

"Top priority"

"A common taxonomy is needed to be able to support evidence synthesis"

"No"

"no"

"Standardization ensures bench-marking across countries and populations and should be attended to highly."

"no"

"as long as it is understandable it is OK for me"

"Consistent terminology will assist patients and clinicians in navigating the plethora of treatment options (and alternative modality names) already available for musculoskeletal pain.".

"No"

"No comments"

"Standardization is important and as stated above, how one defines the method they use is critical. Perhaps this will be guided by frameworks and guidelines? "

"No"

"No"

"There are probably 100 guidelines, including on terminology, for every one person who uses them. "

***Would you like to comment on the research priority of _"The role of organizations and advisory boards in supporting the use of evidence-based telehealth in musculoskeletal conditions"_***

"Seems correct"

"This priority is appropriate for me."

"no"

"No"

"Nil"

"No "

"They can act as umbrella to nurture future prospects"

"No"

"No"

"Important wants earlier questions are answered"

"No "

"No"

"Noble work should received support from organization"

"No comment"

"no"

"none"

"No comments"

"no"

"very important for uptake"

"Merge with 'Research about how to implement telehealth services at the user, clinician and health system level' as these would include organization-level support also."

"No"

"It is common to find outdated infrastructures or personnel with no previous training in telemedicine, which will make difficult to implement or adopt."

"It is important to know the professional statement about the evidence- based telehealth in musculoskeletal conditions. This statement would be a guided for professional performance and continuous development"

"No"

"We need all relevant stakeholders on board. here patient organizations are probably most important"

"No"

"Not specific to eHealth"

"Again, specific to healthcare systems. "

***Would you like to comment on the research priority of _"New developments and advances in telehealth communication and information technologies considering predictive models and the use of artificial intelligence"_***

"Seems correct"

"This subject can be explored further."

"no"

"We have the opportunity to focus on precision medicine and these types of tools would contribute to this. We need to improve the biases of the processed data and find the balance between experimental data and real-world data to be more fine-tuned. "

"AI could have an impact on more accurate diagnosis and treatment recommendations based on clinical presentation and assessment findings. This is both for onsite and virtual care"

"I get annoyed with AI chats about my missing order from Amazon. AI is developing and will no doubt be used in healthcare, but I think that there are other priorities to focus on."

"the future is here in this topic"

"No"

"No"

"Novel advances adds to innovations for larger implications"

"no"

"I often receive adverts on social media, based on my search history and algorithms that are not related at all with my condition. I am in favour of AI in new research models for telehealth without compromising the human touch and detailed and humanised interaction of a practitioner with the person’s medical condition. AI to be used as assistive tool only, never a substitute for human interaction."

"Again I am not really sure what would be studied."

"no"

"no"

"Important"

"This is a more distant goal in chronic pain and msk - is gaining traction for post-op pain and follow-up (ie remote monitoring after cardiac surgeries) but I don't see it being a bit target in MSK in the short term"

"no"

"none"

"No comments"

"No"

"Whilst I do think this priority is important and is the future of telemedicine, I think that consideration of intervention safety and adverse events takes precedence. There is still much to learn in this area, before expanding to AI."

"While predictive models are great, large-scale or big data may be necessary and whether we currently have such data is questionable. Use of AI to predict and newly develop telehealth may come after we first launch a protocol of telehealth approach and through continuous data collection of this approach (therefore being important, but a lower priority)."

"No"

"No comments"

"I think we need to endorse this aspect; as it will be available to patients. "

"No"

"An obvious area in TH"

"No"

"This is unique to eHealth (data availability)"

***Would you like to comment on the research priority of _"_Integration of telehealth devices with electronic health records and cloud databases_"_***

"Seems correct"

"The priority of this topic is suitable for me."

"no."

"We have the opportunity to focus on precision medicine and these types of tools would contribute to this. We need to improve the biases of the processed data and find the balance between experimental data and real-world data to be more fine-tuned"

"Integration allows for a more seamless practitioner experience which can affect the patient experience."

"here is where I am jaded. This would be amazing to have happen, but in the US, HIPAA and different organizations using different systems makes integration so very hard. It hasn't happened yet..."

"the future is here about big data"

"No"

"nil"

"No"

"To gather and create a bank of database for application,integration is major step"

"No"

"Vital in my opinion -it's the way tech is heading, and has been around for a while already. Keeping up to date with tech is surely a high priority."

"I would loved to have seen my physiotherapist specialised app - and the version I was using to track my self management of chronic back pain - updating and uploading my personal results to the GP’s central database, this way all my medical history would be accurately store in the central medical system - rather than a simple summary of physiotherapy results being delivered to my GP in a “hard copy letter” for record keeping."

"It is important that we have inter-operability"

"no"

"no"

"Handling health data requires particular caution. For this reason, very high priority should always be given to personal rights in this context."

"Integration is important"

"Again, I see this more as a principle rather than a program of research"

"No"

"None"

"No comments"

"No"

"interoperability is a key aspect for telehealth"

"Ensuring health records and cloud-based databases fall under safety of telehealth, and it deals with patient privacy and confidentiality - top priority. "

"No"

"As I pointed out before, hospitals, clinics or organization frequently present an outdated infraestructure. Any information that facilitates this process will always be of interest. "

"This aspect is a priority in the health systems for the traceability of the patients and the outcomes of PT interventions"

"This would be helpful. "

"No"

"No"

"Must be secured (GDPR etc) "

"This is the unrealised part of personalised medicine and desperately needs improvement. "

"These are easy and important research to conduct as data is already available for routine patient care. This information can be used to inform future care in an efficient manner"

***Would you like to comment on the research priority of _"_Development of algorithms and analytical approaches for predictive models, personalized, and customized analytics, and devices to improve assessment and management of musculoskeletal conditions_"_***

"Seems correct"

"The priority of this topic is suitable for me."

"No "

"Promise greater effectiveness, efficiency and fewer errors, it is relevant"

"No"

"No"

"pain prediction models - this is relevant"

"No. "

"No"

"Care must be take to avoid too much complicated terms with low productivity"

"no"

"Not sure about this one and how I feel about the accuracy of algorithms."

"Again, provided that the technology is not introduced as a substitute of the human interaction with a clinician."

"no"

"no"

"no"

"This is similar to AI area covered earlier"

"no"

"none"

"No comments"

"no"

"no"

"This is a requirement in the Quality Assurance of the telehealth services"

"This may be more important at a later stage. "

"No"

"Split the title"

"Much like the AI question above"

"no"

***Would you like to comment on the research priority of _"_Would you like to add any missing research priorities not mentioned before?_"_***

"No"

"No"

"Nosynthesis"

"No"

"Standardizing telehealth documentation, treatment plans, terminilogies"

"Inequities and disparities with virtual care delivery"

"i think these priorities are a great start!"

"No"

"integration of established outcome measures and telehealth/medical records and exercise prescription tools"

"I am not sure if it comes under enablers of telehealth questions. Acceptability/Feasibility of the tehealth by a patient could be another reserach area."

"It's essential to emphasis the mutual dependency of tele health and contact based rehabilitation for better outcomes"

"no"

"How to increase the outreach of telehealth services to remote areas"

"No"

"No"

"I think we should do qaulitative research on the factors that influence success in telehealth that influence"

"No"

"Interprofessional education and health care supporting telehealth"

"Implementation research (health services research, pragmatic trials) with telehealth"

"no"

"no"

"no"

"no "

"I am so excited that all research priorities of interest to me are covered"

"no"

"No - I think they've all been covered."

"what about organizations? we should seek to measure the maturity level of health institutions to offer telemedicine"

"Costs, Integration of teleheath with other technologies (eg wearable sensors, virtual reality)"

"perhaps something around regulatory environments for telehealth practice across regions. We dont really have a clear understanding of this and how to shift regulatory barriers."

"Access to the healthcare of e-health for ordinary patients with musculoskeletal pain conditions."

While I don't recommend research priorities that are too discipline-specific, I would like to see research priorities that address how interdisciplinary or multidisciplinary approaches can be incorporated into the telehealth model. Perhaps this can fall under "Identify, explore, and implement the most suitable business models to support the delivery of…" or "Standardization of telehealth-related terms and the development of frameworks for…" or "Research about how to implement telehealth services at the user, clinician and health system…"

"In low income county, strategies"

"None"

"No"

"Research with cost effectiveness of digital health interventions it’s really important. Also digital intervention to promote self management approach for MSK. "

"Maybe clinician attitudes and the role of clinicians in telehealth"

"effectiveness and cost-effectiveness of different interventions (I think these were available in the first round) "
