# Supplementary material for: One step at a time. Shaping consensus on research priorities and terminology in telehealth in musculoskeletal pain: an international modified e-Delphi study
Source: BMC Musculoskelet Disord. 2023 Oct 3;24:783. doi: 10.1186/s12891-023-06866-0 (PMC10546725; doi:10.1186/s12891-023-06866-0)
Supplement: Supplementary file 12 — Additional file 12: Supplementary file 12. Feedback with all the raw results of the third round. [file 12891_2023_6866_MOESM12_ESM.docx]

**Supplementary file 12. Feedback with all the raw results of the third round.**

**Which country do you currently reside in?**

**What is your main Stakeholder Group?**

**What is your gender?**

**Highest level of education**

**Panel members - Researchers**

**What is your area of research?**

**What is the main treatment approach that you research in telehealth?**

**What telehealth modality(s) have you been researching?**

**Panel members - Consumer representative**

**Which of the musculoskeletal conditions did you receive telehealth for?**

**Which modality(s) of telehealth have you experienced?**

**Panel members - Developer and industry partner**

**What is your area of work?**

**What is the main treatment approach that you work in telehealth?**

**What telehealth modality(s) have you been working with?**

**Standard terminologies for telehealth in musculoskeletal pain research**

**Do you support the use of the term ___ as standard terminology?**

| **Third Round** | | |
| --- | --- | --- |
| Summary to support the use of the term as standard telehealth terminology in musculoskeletal pain suggested by the panel members. | | |
| Nº | Telehealth terms | Round 3 |
|  |  | Strong agreement (> 80 %) |
| 1 | * | - |
| 2 | * | - |
|  |  |  |
|  |  | Moderate agreement (70% - 80%) |
| 1 | Digital health intervention | 74.6% |
| 2 | Telemedicine | 71.6% |
| 3 | Telerehabilitation | 70.1% |
|  |  |  |
|  |  | Low agreement (50% - 70%) |
| 1 | Online consultations | 67.2% |
| 2 | Mobile health app (mHealth) | 67.2% |
| 3 | Electronic health (eHealth) | 66.4% |
| 4 | Remote monitoring | 59.7% |
| 5 | Videoconferencing | 57.5% |
| 6 | Video consultations | 57.5% |
| 7 | eHealth Intervention | 57.4% |
| 8 | Teleconsultation | 51.5% |
| 9 | Online pain management | 50% |
| 10 | Online healthcare | 50% |
| - | - | - |
|  |  | No agreement (< 50%) |
| 1 | Remote home-based exercise program | 46.3% |
| 2 | Digital rehabilitation | 40.3% |
| 3 | Digital health rehabilitation | 40.3% |
| 4 | Asynchronous Care Delivery Platform | 16.4% |
|  |  |  |
| *No new terminologies reached strong agreement level (> 80%) considered for final list | | |

**Overall suggestions, re-wording, and comments for any terminology provided by the panel members.**

1. "Nonee"
2. "The problem with much of your wording is that it does not include telephone without video. This is most ubiquitous an most under utilized resource"
3. "No"
4. "No"
5. "This is more a comment as opposed to a suggestion: When the prefix 'tele' is used, it makes one think of telephone as the means of communicating. In my experience telephone consultation can be quite limited as compared to video."
6. "No"
7. "Teleconsultation to change to online consultation.
8. "Virtual Care"
9. "no?"
10. "telehealth versus telemedicine"
11. "No"
12. "No"
13. "healthcare metaverse"
14. "No"
15. "Telephysiotherapy"
16. "no"
17. "REMOTE LEARNING - REMOTE REHABILITATION"
18. "Digital health care interventions should be used as standard term"
19. "not at all"
20. "None"
21. "N/A"

**Research priorities for telehealth in musculoskeletal pain research**

**How much do you agree this should be a research priority?**

**Research priorities for telehealth in musculoskeletal pain research**

**How important is this research priority?**

| **Third Round** | | | |
| --- | --- | --- | --- |
| Summary for telehealth research priorities in musculoskeletal pain suggested by panel members. | | | |
| Nº | Telehealth research priorities | Round 3 | |
|  |  | Strong agreement  (>80%) | Level of importance (Mean/SD) |
| 1 | Research and development of strategies for using information and communication technology to facilitate access to individuals with musculoskeletal conditions in remote or rural regions | 91.8% | 8.3 (1.5) |
| 2 | Research on reliability and validity of clinical assessment and diagnostic tests administered via telehealth (compared to in-person testing) in individuals with musculoskeletal conditions | 88% | 8.1 (1.8) |
| 3 | Identification of mediators contributing to the effects of telehealth-delivered treatments | 82% | 7.5 (1.8) |
| 4 | Investigation of adverse events and patient safety during telehealth encounters for musculoskeletal conditions | 80.6% | 7.7 (2) |
|  | - | - | - |
|  |  | Moderate agreement  (70% - 80%) | Level of importance  (Mean/SD) |
| 1 | Research on suitable patient-oriented research outcome measures for telehealth in individuals with musculoskeletal conditions | 79.2% | 7.6 (1.8) |
| 2 | Translation, dissemination and communication developed with all parties involved | 75.3% | 7.5 (2.1) |
| 3 | New developments and advances in telehealth communication and information technologies considering predictive models and the use of artificial intelligence | 73.9% | 7.2 (2.2) |
| 4 | Data science initiative to support the use of telehealth in musculoskeletal conditions | 73.1% | 7.4 (2) |
| 5 | The role of organizations and advisory boards in supporting the use of evidence-based telehealth in musculoskeletal conditions | 70.9% | 7.1 (2.2) |
| - | - | - | - |
|  |  | Low agreement  (50% - 70%) | Level of importance  (Mean/SD) |
| 1 | Development and testing of innovative business models to support the delivery of telehealth in musculoskeletal conditions. | 66.4% | 6.8 (2.3) |
| 2 | Identification of clinician (health professional) characteristics and beliefs that affect response to management via telehealth | 66.4% | 6.8 (2.3) |
| - | - | - | - |
|  |  | No agreement (< 50%) | Level of importance  (Mean/SD) |
|  | - | - | - |

**Comments and suggestions for research priorities provided by the panel members**

***Would you like to comment on the research priority of _"Research and development of strategies for using information and communication technology to facilitate access to individuals with musculoskeletal conditions in remote or rural region"***

1. "No"
2. "no"
3. "no"
4. "No"
5. "No"
6. "No"
7. "No"
8. "Sometimes people who live in rural areas don't have access a tecnology and information"
9. "no"
10. "I would include others who may not have access for different reasons."
11. "We must make access available to everyone. Every person has a chance to be brilliant, no matter geographics."
12. "Important"
13. "It will provide the people in remote/rural setups, the much needed access to healthcare. COVID has shown how it is the need of the hour"
14. "shorten make more punchy"
15. "This is a priority research, especially in Brazil, where it is difficult to have access to the entire population to conduct studies and be able to generalize results of future research that need representative samples of the country.
16. "Facilitating digital rehabilitation access"
17. "Very high priority, but equally important to facilitate access for everyone."
18. "NA"
19. "Two points--in many rural areas there is not good broadband and thus telephone needs to be the technology. We need ways of making healthcare equitable be it for rural folks, those without transportation, or those with disability for which in person is difficult if not impossible. Also health care institutions are often not the most friendly so sometime people avoid in person for this reason."
20. "I thi k we need to know that rural regions will want to use this method first"
21. "The safety aspect is important no matter whether the patient is in remote or urban setting. Agree access can be a bigger issue in remote and rural areas."
22. "No"
23. "Access to care is a barrier in remote/rural regions based on geography. Access is improved with virtual care however internet connectivity based on geography can be a similar barrier."
24. "No"
25. Due to financial and time constraints linked to accessing health care for individuals living in rural or more remote locations, this has always seemed like the best possible solution to provide access to health care, advice and monitoring."
26. "No"
27. "I understand why this topic may be of importance to people in large countries, but I believe it is more important to reach people who have difficulty transporting themselves to clinics, or few financial resources for transport/time/fee for service."
28. "While important, this has to be specific to the local context / environment / government policies; COVID-19 should also have identified that telehealth should not only have a place for those living in rural/remote areas, but for any individual that has difficulty accessing in-person services (or wishes to access their care via telehealth regardless)"
29. "appropriate."
30. "No"
31. "No"
32. "Individual care in form of physical contact still is most important for effective and holistic health care management"
33. "We are using telehealth, and there is much need in rural areas. But we need to make sure that the interventions/interactions are safe and helpful."
34. "Please focus on chronic musculoskeletal conditions."
35. 'would recommend defining access a bit more specifically. For example, to facilitate access to rehabilitation care...for individuals with...”
36. "None"
37. "This leverages telehealth to advance equity"

"***Would you like to comment on the research priority of _"Investigation of adverse events and patient safety during telehealth encounters for musculoskeletal conditions"***

1. "Should know avoid adverse effects"
2. "No"
3. "This is important to know if teleintervention is safe"
4. "This would be useful to prevent recurrence"
5. "the patient can misunderstood the performance of exercise and repetition,timing or frequency of the exercises"
6. "Important to include all types of adverse events."
7. "I think already exist some studies like that"
8. "NA"
9. "No matter what the research, looking at adverse events should be apart. Separate studies in adverse events are probably not necessary"
10. "Suggest consider medical, physical and emotional safety considerations."
11. "No"
12. "Think of what Can be threatening patient safety in MSK patients, makes this relatively low priority"
13. "Prevalence of adverse events, prevention of adverse events, preparation for adverse events and what to do if an adverse event happens (appropriate methods of escalation) all need to be understood with virtual care"
14. "No"
15. "When following routine red flag and yellow flag screening for first time consultations and ensuring that emergency contacts and/ or caregivers are present, this could be less of a concern than expected."
16. "No"
17. "The investigation of adverse events should be incorporated into any research, whether it is telehealth or in-person care, and presented as part of the findings. Again, strategies around processes to manage urgent care needs are very context specific, so beyond some general guidelines/tips, this word needs to completed at a local level."
18. "appropriate"
19. " no"
20. "no"
21. "Investigation for detailed assessment is priority."
22. "It is important that we are making sure patients are safe."
23. "no adverse events for telehealth encounter but it could be a screening to understand if you solve the problem in that way or need to have a visit in presence"
24. "None"
25. "Needs to be addressed to sustain telehealth broadly"

***Would you like to comment on the research priority of _"Research on suitable patient-oriented research outcome measures for telehealth in individuals with musculoskeletal conditions"***

1. "Patient-oriented research encourages patients as partners in research."
2. "There is a huge amount of work on outcomes already: they could be used. There are no perfect outcomes for all situations; they rather depend on the question/s, the population/s, and the treatment methods."
3. "No"
4. "No"
5. "No"
6. "No"
7. "No
8. "No
9. "No"
10. "As I train people, they need MSK ultrasound or other diagnostic test to measure progress. I think "teleradiology" may exist."
11. "very much relevant"
12. "Will help the clinician in quantification and understanding the patients feedback"
13. "no"
14. "to identify important outcomes of this intervention."
15. "it is important to evaluate the effectiveness of any healthcare intervention"
16. "should be comparable to PROMS of non-digital interventions as outlined by OMERACT"
17. "Important to allign these w PROMs for non-telehealth interventions"
18. "Very important. Perhaps using artificial intelligence"
19. "NA"
20. "We already have the measures. People just need to use them."
21. "No need to produce a special outcome measure for video interaction"
22. "I think we should start with the measure we already know and see how they work"
23. "I think the 'experience' component is important to research; however, other outcome measures already in use such as the DASH can be used for in-person encounters and telehealth encounters."
24. "Patients must be an equal part of this research endeavour - from the outset."
25. "Good tools are needed and Patient outcome measurements are political high value right now"
26. "Tests that are administered electronically need to be understood from an accuracy/validity/reliability perspective."
27. "no"
28. "Especially presenting these outcome measures in a user-friendly and "device" friendly manner (USSD/ bot codes for low tech and low cost options and interactive screening and measuring options for higher tech)"
29. "No"
30. "Definitely need more of this across the spectrum of MSK conditions/body regions - research needs to not only present agreement results, but also provide detailed information on how the tests were modified, how the tests are performed in-person, and what constitutes a positive/negative test. All agreement studies also need an inter-rater reliability arm with a second in-person assessment to better understand the reliability in response to the change in test/delivery medium, as opposed to individual assessor application & interpretation of the test."
31. "Existing may be sufficient for now"
32. "appropriate."
33. "No"
34. "No "
35. "No comments"
36. "Validating current outcome measures in an outpatient setting would be appropriate."
37. "None"
38. Need to be able to measure for effectiveness studies

***Would you like to comment on the research priority of _"Research on reliability and validity of clinical assessment and diagnostic tests administered via telehealth in individuals with musculoskeletal conditions"***

1. "Reliability and validity ensures the appropriateness of these tests and for standardization"
2. "Most existing clinical assessment is dismal even in the best circumstances. Reliability and validity are not static properties but depend on the application of the assessment. This needs considerably more critical thought."
3. "No."
4. "No"
5. "No"
6. "No"
7. "No"
8. "No"
9. "No"
10. "As I train people, I am able to test, assess and measure current real time condition of patient issue. The patient is participating in "Telemedicine self management" or "TELESELF"
11. "Reliable."
12. "No"
13. "we need reliable data"
14. "It would be good to have consistency, but clinical tests are often inaccurate or vary between administrators in face to face settings too."
15. "NA"
16. "Much of this work has already been done starting with the HAQ more then 35 years ago."
17. "not sure it can be compared with in person tests but would want to know if the consultation resulted in similar conclusion"
18. "We absolutely need to know the validity and reliability before we offer this type of consultation"
19. "I think this is important; validating clinical findings in-person with telehealth. Will help guide what is appropriate to do in telehealth forum and what is best done in-person."
20. "No."
21. "I still find the clinical assessment and tests should be done at the clinics…. Not just for the quality of testing, but for the benefit of patient-clinician relationship to ensure compliance. Take that into the research"
22. "This is a high priority research question as we base our virtual assessment on both the subjective and objective findings. The subjective component remains unchanged however the objective is modified for a virtual exam. Is the functional exam accurate in determining myotomal weakness and/or self administered resistance?"
23. "No"
24. "As previous comment made."
25. "appropriate"
26. "No"
27. "At this point, it is very important to validate assessment methods."
28. "Validity and reliability should be most important aspect."
29. "My bias is that for MSK conditions, telehealth is as helpful in person in many cases. But this needs to be studied (even if it means my bias is wrong)"
30. "it is necessary to do to understand what it functions or not"
31. "None"

***Would you like to comment on the research priority of _"Identification of clinician (health professionals) characteristics and beliefs that affect response to management via telehealth"***

1. "No"
2. "Unlikely to be particularly different from those that affect response face to face; that at least would be where to start."
3. "No"
4. " No "
5. " No."
6. " No"
7. " No "
8. "The professional must be competent and have to know using the tools of internet"
9. "No"
10. "For good health "
11. "No "
12. "important, but not now "
13. "Any clinician's beliefs will have an influence on their patient management choices "
14. "Could be obvious answer as allied health needs more physical touch etc "
15. "NA"
16. "It is often clinicians who stand in the way of telehealth based on beliefs and past practice."
17. " I would like to see the research in healthprofessional beliefs and attitudes towards telehealth as we know this will affect outcomes"
18. "If are trying to 'scale up' telehealth this is important. However, if doing things in isolation, some clinicians will simply choose not to use telehealth for a variety of reasons, including lack of confidence."
19. "No"
20. "Find the objectives first, and then if needed, look at influence from clinicians believes."
21. "Uptake and adoption of virtual care is based on clinician acceptance and comfort with virtual care and technology"
22. "No"
23. "Not specific to eHealth"
24. "No"
25. "appropriate."
26. "No"
27. "Suitability is essential for better outcomes"
28. "I think that this would be helpful research in all settings, but I think knowing more about the safety, measuring outcomes, and knowing the validity assessment via telehealth would be a higher priority."
29. "the new generation of doctors born in a digital era will use as normal"
30. "This is critical - from personal experience, clinicians that do well with telehealth believe in it in the first place."
31. "None"

***Would you like to comment on the research priority of _"Identification of mediators contributing to the effects of telehealth-delivered treatments"***

1. "These mediators can help limit the chronicity of patients' symptoms when identified before and during the delivery of treatments"
2. "These are most unlikely to be common to all telehealth interventions regardless of all the other variables: population, intervention, etc etc."
3. "No"
4. "No "
5. "No"
6. "No"
7. "No"
8. "It is necessary to analize the final results of all researches"
9. "lets identify the best."
10. "Perfection is important by a mediator"
11. "No "
12. "This is important to identify the mediators that can influence in the intervention."
13. "Most health professions would look more holistically at contributing factors to a patient's condition "
14. "Sorry, I rate all of these as highly important - but they are..... "
15. "NA"
16. "We know a lot about the mediation already but this has been largely dropped from studies and interventions should be built on theory."
17. "This is very complicated and I think comes later in the research spectrum once we understand more basic things about telehealth."
18. "No "
19. "If I understand this right, this is to find out, who can benefit from telehealth and more importantly if there are factors that show who can not? If so, it is important "
20. "No"
21. "appropriate"
22. "No "
23. "No"
24. "Most important factor "
25. "Please list some mediators as examples"
26. "None."

***Would you like to comment on the research priority of _"Translation, dissemination and communication developed with all parties involved"***

1. "Highly relevant because it enhances co-production."
2. "I don't really think this is research."
3. "Its not clear to me what the research question or objective is here"
4. "No"
5. "I think this research priority is difficult to interpret. Translation, dissemination and communication of what? and to which group?"
6. "Can a therapeutic alliance be made virtually? Based on my clinical experiences it most definitely can. The patient introduces me to their family, pets, shows me their house etc. It has been better than I expected. however, patients who choose virtual may be more comfortable in their house and/or more comfortable with technology."
7. "No"
8. "no"
9. "no"
10. "Yes it is important"
11. "If there are lessons to be learned it is important that they are communicated"
12. "NA"
13. "This is a very under studied area especially for MS diseases. One of the big problem is that while there are interventions that are evidenced base, been shown to be effective in clinical trials, there are few funding sources to allow for dissemination. These need to be built into payor system and a study done on why there is so much resistance."
14. "I believe in integrated KT therefore it should be happening at the beginning"
15. "Including patients is very important in both the research and in the translation."
16. "No."
17. "It is of high importance, but you need to focus on what works before you need to prioritise communication strategy and research translation."
18. "This is very important to ensure that the needs, expectations and competencies of all parties are considered and addressed, otherwise resources could be wasted on inappropriate programmes that are not sustainable."
19. "No"
20. "There needs to be much more work attempted in the co-design space, where our patients are advocating for the benefits and needs of telehealth as part of their care. Many MSK telehealth studies that have been published in response to COVID-19 have completed ignored the patient perspective, used simple survey techniques that have not captured the depth of their experience, and not involved the consumer in the development and interpretation of results. We also need to think about different ways to disseminate results (outside of a manuscript or conference) that can reach the hearts & minds of our patients so that they can make informed choices about their care, instead of having to entirely rely on their clinicians' opinion."
21. "Not specific to eHealth"
22. "appropriate"
23. "No"
24. "No comments"
25. "Evidence-based translation, dissemination, and communication"
26. "This could be defined more specifically. Perhaps translation, dissemination and communication of impact of telehealth on management of msk conditions should be ensured with input from patients and health team members"
27. "None"

***Would you like to comment on the research priority of _"Development and testing of innovative business models to support the delivery of telehealth in musculoskeletal conditions"***

1. "No"
2. "Little experience of reimbursement systems. There are healthcare systems outside the US!"
3. "no"
4. "Business word should be deleted"
5. "The development of business models seems, at least to me, not to be research related, and therefore can't be a research priority - unless we are defining research priorities very broadly to include market research, for example."
6. "Models for long-term sustainability of interventions is a key challenge - researchers develop and evaluate helpful interventions but the interventions often languish in universities because there is no system (a) to make them widely available or (b) to keep the content updated."
7. "No"
8. "No"
9. "No "
10. "Innovations are ever important factor."
11. "importante para centros de salud, no para clínicos"
12. "Often technology and innovations move and develop faster in business settings"
13. "without sustainable business models we are never going to get clinicians on board"
14. "NA"
15. "see comment above. I am not sure we need more study, we may need much more advocacy"
16. "No"
17. "Really relevant for implementation, but the research on what works well is higher/first priority"
18. "Very important, especially in lower income/ resourced countries where data and communication technologies costs are expensive. Community based "hubs" such as a room in the local library/ clinic equipped with the technology to attend specialist care or follow up for those who do not have the resources to use tele services"
19. "No"
20. "Particularly within the public health sector, we need to continue to look at care models using remote monitoring / asynchronous mediums to reduce the need for direct patient care (to manage/support waitlists), while still achieving similar outcomes and pushing for a focus on active participation and self-management strategies, particularly for chronic MSK conditions."
21. "appropriate."
22. "No"
23. "Innovation is key to sustainability and better results"
24. "This needs to happen across healthcare."
25. "i don't like the use of business model. One aspect of it is service delivery which is widely accepted term"
26. "None"

***Would you like to comment on the research priority of _"The role of organizations and advisory boards in supporting the use of evidence-based telehealth in musculoskeletal conditions"***

1. "No"
2. "Few people read guidelines; fewer apply them"
3. "No"
4. "Such boards and organizations may be more influential in making interventions available (sustainable)"
5. "No"
6. "No"
7. "No"
8. "Evidence based practice is important"
9. "no"
10. "If telehealth is effective, it is important that acknowledged bodies advocate the use of it"
11. "NA"
12. "I think if this is not supported it will be hard to be successful in rural regions"
13. "Involvement of regulatory colleges is important, to be sure what is developed meets standards."
14. "No"
15. "No"
16. "I don't think that this should necessarily be a research priority, rather work that is done at the appropriate level of government/ organisation."
17. "appropriate"
18. "No "
19. "Yes..its important"
20. "I like the idea of patients having a say in their care!"
21. "None "

***Would you like to comment on the research priority of _"New developments and advances in telehealth communication and information technologies considering predictive models and the use of artificial intelligence"***

1. "no"
2. "Given the anomalies that can be built into AI (ethnic and cultural biases, for instance), this is an important area."
3. "No"
4. "No"
5. "Personalised health-care is the way of the future"
6. "no"
7. "No
8. "no"
9. "Artificial intelligence is the future perspective"
10. "No"
11. "important, but research about current treatments using ICT is more important"
12. "Digital models and AI can help filter and signpost patients towards the correct services for their needs"
13. "NA"
14. "One lesson learned from the pandemic is the role of isolation and depression in all diseases. I think that AI just adds to this. We need to look at how to use technology to enhance human touch."
15. "this is an interesting area and will be challenging but it is what is coming down the track and we need to be confident it is appropriate"
16. "Will be important once we know more"
17. "No"
18. "Important to be the among the first to use AI for MSK Healy"
19. "Leveraging advances in technology and use of AI have potential to improve the patient and provider experience with virtual care delivery."
20. "Very important"
21. "appropriate"
22. "No"
23. "I believe it is necessary, but it is important to consider the limitations of AI at the level of data entry, which can have many cognitive biases of the programmer, and to ignore certain contextual factors that can have great relevance in decision making."
24. "Need of hour"
25. "No"
26. "I am skeptical about AI, but agree that it should be studied!"
27. "None."

***Would you like to comment on the research priority of _"Data science initiative to support the use of telehealth in musculoskeletal conditions"***

1. "Not clear what the research element is"
2. "no"
3. "No"
4. "No"
5. "No"
6. "Data science is important for telehealth."
7. "different in developed countries than developing countries"
8. "Data is important - it is good to have relevant data collated and analysed on a larger scale"
9. "Very important to secure patient ownership of data and at the same time data available for research"
10. "NA"
11. "No"
12. "Important to make sure MSK conditions/patients are monitored together with all other conditions…. I guess this is not (just) MSK research but multidisciplinary research."
13. "It is very current"
14. "appropriate"
15. "No"
16. "yes"
17. "we need data."
18. "I think it was not a good idea to merge these research priorities. In my opinion, making collecting data and making it available is extremely important, but AI/machine learning stuff at this stage is more likely to lead us astray at this stage"
19. "None"
