# Supplementary material for: One step at a time. Shaping consensus on research priorities and terminology in telehealth in musculoskeletal pain: an international modified e-Delphi study
Source: BMC Musculoskelet Disord. 2023 Oct 3;24:783. doi: 10.1186/s12891-023-06866-0 (PMC10546725; doi:10.1186/s12891-023-06866-0)
Supplement: Supplementary file 13 — Additional file 13: Supplementary file 13. A. Stability analysis of non-consensus terminologies items from the e-Delphi survey. B. Stability analysis of non-consensus research priorities items from the e-Delphi survey. [file 12891_2023_6866_MOESM13_ESM.docx]

| **Supplementary file 13 A. Stability analysis of non-consensus terminologies items from the e-Delphi survey** | | | | | | | | | |
| --- | --- | --- | --- | --- | --- | --- | --- | --- | --- |
|  | Round | Likert scale (%) | | | | | Mean (SD) | Median (IQR) | Wilcoxon Signed |
|  |  | 1 | 2 | 3 | 4 | 5 |  |  | Rank Test |
| *Terminologies* | | | | | | | | | |
| Mobile health app (mHealth) | 2 | 4 | 9 | 14 | 35 | 43 | 3.99 (1.11) | 4.00 (3.00 – 5.00) | 0.03 |
|  | 3 | 5 | 16 | 17 | 25 | 42 | 3.79 (1.25) | 4.00 (3.00 - 5.00) |  |
| Digital health intervention | 2 | 2 | 7 | 17 | 51 | 28 | 3.91 (0.93) | 4.00 (3.50 - 5.00) | 0.47 |
|  | 3 | 2 | 5 | 16 | 49 | 33 | 4.01 (0.91) | 4.00 (4.00 - 5.00) |  |
| Online consultations | 2 | 5 | 13 | 14 | 42 | 31 | 3.77 (1.14) | 4.00 (3.00 - 5.00) | 0.44 |
|  | 3 | 3 | 6 | 25 | 40 | 31 | 3.86 (1.00) | 4.00 (3.00 - 5.00) |  |
| Telemedicine | 2 | 3 | 9 | 18 | 35 | 40 | 3.95 (1.07) | 4.00 (3.00 - 5.00) | 0.55 |
|  | 3 | 5 | 9 | 19 | 31 | 41 | 3.90 (1.16) | 4.00 (3.00 - 5.00) |  |
| Teleconsultation | 2 | 3 | 8 | 20 | 45 | 29 | 3.85 (1.00) | 4.00 (3.00 - 5.00) | 0.00 |
|  | 3 | 5 | 18 | 34 | 27 | 21 | 3.39 (1.13) | 3.00 (3.00 - 4.00) |  |
| Video consultations | 2 | 6 | 13 | 18 | 47 | 21 | 3.61 (1.11) | 4.00 (3.00 - 4.00) | 0.84 |
|  | 3 | 6 | 14 | 25 | 31 | 29 | 3.60 (1.19) | 4.00 (3.00 - 5.00) |  |
| Electronic health (eHealth) | 2 | 6 | 9 | 20 | 39 | 31 | 3.76 (1.14) | 4.00 (3.00 - 5.00) | 0.92 |
|  | 3 | 8 | 10 | 18 | 30 | 39 | 3.78 (1.25) | 4.00 (3.00 - 5.00) |  |
| eHealth Intervention | 2 | 4 | 5 | 25 | 45 | 26 | 3.80 (0.99) | 4.00 (3.00 - 4.50) | 0.14 |
|  | 3 | 4 | 16 | 22 | 34 | 29 | 3.65 (1.15) | 4.00 (3.00 - 5.00) |  |
| Telerehabilitation | 2 | 6 | 17 | 16 | 27 | 39 | 3.72 (1.27) | 4.00 (3.00 - 5.00) | 0.00 |
|  | 3 | 2 | 11 | 18 | 26 | 48 | 4.02 (1.10) | 4.00 (3.00 - 5.00) |  |
| Digital rehabilitation | 2 | 7 | 10 | 22 | 48 | 18 | 3.57 (1.09) | 4.00 (3.00 - 4.00) | 0.00 |
|  | 3 | 11 | 18 | 35 | 25 | 16 | 3.16 (1.19) | 3.00 (2.00 - 4.00) |  |
| Online healthcare | 2 | 7 | 9 | 25 | 46 | 18 | 3.56 (1.08) | 4.00 (3.00 - 4.00) | 0.03 |
|  | 3 | 8 | 22 | 23 | 37 | 15 | 3.28 (1.17) | 3.00 (2.00 - 4.00) |  |
| Remote monitoring | 2 | 9 | 12 | 26 | 35 | 23 | 3.49 (1.20) | 4.00 (3.00 - 4.00) | 0.26 |
|  | 3 | 5 | 13 | 23 | 38 | 26 | 3.64 (1.12) | 4.00 (3.00 - 4.50) |  |
| Remote home-based exercise program | 2 | 11 | 14 | 23 | 40 | 17 | 3.36 (1.21) | 4.00 (3.00 - 4.00) | 0.13 |
|  | 3 | 10 | 20 | 29 | 33 | 13 | 3.18 (1.16) | 3.00 (2.00 - 4.00) |  |
| Online pain management | 2 | 8 | 17 | 25 | 37 | 18 | 3.38 (1.17) | 4.00 (3.00 - 4.00) | 0.98 |
|  | 3 | 6 | 14 | 30 | 41 | 14 | 3.41 (1.06) | 4.00 (3.00 - 4.00) |  |
| Videoconferencing | 2 | 13 | 24 | 13 | 36 | 19 | 3.23 (1.32) | 4.00 (2.00 - 4.00) | 0.29 |
|  | 3 | 14 | 18 | 14 | 34 | 25 | 3.36 (1.36) | 4.00 (2.00 - 4.00) |  |
| 1) Strongly disagree, 2) Disagree, 3) Neither agree or disagree, 4) Agree, 5) Strongly agree; SD) Standard deviation IQR) Interquartile range. | | | | | | | | | |

| Additional file 13 B. Stability analysis of non-consensus research priorities items from the e-Delphi survey | | | | | | | | | |
| --- | --- | --- | --- | --- | --- | --- | --- | --- | --- |
|  | Round | Likert scale (%) | | | | | Mean (SD) | Median (IQR) | Wilcoxon Signed |
|  |  | 1 | 2 | 3 | 4 | 5 |  |  | Rank Test |
| *Research priorities* | | | | | | | | | |
| Research on suitable patient-oriented research outcome measures for telehealth in individuals with musculoskeletal conditions | 2 | 1 | 5 | 23 | 37 | 39 | 4.03 (0.93) | 4.00 (3.00 - 5.00) | 0.10 |
|  | 3 | 0 | 3 | 15 | 46 | 41 | 4.19 (0.78) | 4.00 (4.00 - 5.00) |  |
| Identification of clinician (health professional) characteristics and beliefs that affect response to management via telehealth | 2 | 2 | 6 | 32 | 31 | 34 | 3.85 (1.00) | 4.00 (3.00 - 5.00) | 0.80 |
|  | 3 | 4 | 4 | 25 | 47 | 25 | 3.81 (0.97) | 4.00 (3.00 - 4.00) |  |
| Translation, dissemination and communication developed with all parties involved | 2 | 2 | 7 | 25 | 31 | 40 | 3.95 (1.03) | 4.00 (3.00 - 5.00) | 0.03 |
|  | 3 | 0 | 3 | 22 | 38 | 42 | 4.13 (0.84) | 4.00 (4.00 - 5.00) |  |
| Development and testing of innovative business models to support the delivery of telehealth in musculoskeletal conditions. | 2 | 0 | 8 | 31 | 39 | 27 | 3.81 (0.91) | 4.00 (3.00 - 5.00) | 0.51 |
|  | 3 | 1 | 8 | 26 | 51 | 19 | 3.75 (0.87) | 4.00 (3.00 - 4.00) |  |
| The role of organizations and advisory boards in supporting the use of evidence-based telehealth in musculoskeletal conditions | 2 | 0 | 7 | 26 | 37 | 35 | 3.95 (0.92) | 4.00 (3.00 - 5.00) | 0.48 |
|  | 3 | 2 | 6 | 24 | 42 | 31 | 3.90 (0.96) | 4.00 (3.00 - 5.00) |  |
| New developments and advances in telehealth communication and information technologies considering predictive models and the use of artificial intelligence | 2 | 1 | 2 | 29 | 36 | 37 | 4.01 (0.89) | 4.00 (3.00 - 5.00) | 0.71 |
|  | 3 | 0 | 2 | 23 | 47 | 33 | 4.06 (0.78) | 4.00 (4.00 - 5.00) |  |
| Integration of telehealth devices with electronic health records and cloud databases* | 2 | 1 | 3 | 19 | 33 | 49 | 4.20 (0.90) | 4.00 (4.00 - 5.00) | 0.05 |
| Data science initiative to support the use of telehealth in musculoskeletal conditions* | 3 | 0 | 2 | 24 | 50 | 29 | 4.01 (0.76) | 4.00 (3.50 - 5.00) |  |
| Development of algorithms and analytical approaches for predictive models, personalized, and customized analytics, and devices to improve assessment and management of musculoskeletal conditions* | 2 | 0 | 2 | 28 | 44 | 31 | 3.99 (0.80) | 4.00 (3.00 - 5.00) | 0.82 |
| Data science initiative to support the use of telehealth in musculoskeletal conditions* | 3 | 0 | 2 | 24 | 50 | 29 | 4.01 (0.76) | 4.00 (3.50 - 5.00) |  |
| 1) Strongly disagree, 2) Disagree, 3) Neither agree or disagree, 4) Agree, 5) Strongly agree; SD) Standard deviation; IQR) Interquartile range; *Previously merged research priority* | | | | | | | | | |
