# Supplementary material for: One step at a time. Shaping consensus on research priorities and terminology in telehealth in musculoskeletal pain: an international modified e-Delphi study
Source: BMC Musculoskelet Disord. 2023 Oct 3;24:783. doi: 10.1186/s12891-023-06866-0 (PMC10546725; doi:10.1186/s12891-023-06866-0)
Supplement: Supplementary file 14 — Additional file 14: Supplementary file 14. A. Sensitivity analysis of the first round for the support of the research priorities in musculoskeletal pain. B. Sensitivity analysis of the second round for the support of the research priorities in musculoskeletal pain. C. Sensitivity analysis of the third round for the support of the research priorities in musculoskeletal pain. D. Sensitivity analysis of the first round for the support of the use of the term as standard terminology in musculoskeletal pain. E. Sensitivity analysis of the second round for the support of the use of the term as standard terminology in musculoskeletal pain. F. Sensitivity analysis of the third round for the support of the use of the term as standard terminology in musculoskeletal pain. [file 12891_2023_6866_MOESM14_ESM.docx]

| **Supplementary file 14 A. Sensitivity analysis of the first round for the support of the research priorities in musculoskeletal pain** | | |
| --- | --- | --- |
| **Telehealth research priorities** | **All panel members**  **(n = 160)** | **Non-ISC panel members**  **(n = 150)** |
| **Strong agreement** |  |  |
| Research about how to implement telehealth services at the user, clinician and health system level | 147 (92) | 137 (91) |
| Effectiveness of treatment approaches delivered via telehealth in the management of musculoskeletal conditions | 143 (89) | 134 (89) |
| The cost-effectiveness of telehealth treatments for musculoskeletal conditions | 141 (88) | 131 (87) |
| Equity research on interventions to improve access, treatment or clinical outcomes to telehealth services for disadvantaged or historically underserved populations with musculoskeletal conditions | 141 (88) | 131 (87) |
| Design and evaluation of curricula to train students and health care practitioners in the provision of telehealth that conforms to core capability frameworks | 134 (84) | 125 (83) |
| Research on health literacy, eHealth literacy, technology literacy and identifying relevant factors for patients with musculoskeletal conditions engaging in telehealth (eg, barriers and facilitators) | 131 (82) | 122 (81) |
| **Moderate agreement** |  |  |
| Identification of patient characteristics that affects the response to treatments delivered by telehealth* | 128 (80) | 121 (81) |
| Investigation of harms and adverse events during telehealth encounters for musculoskeletal conditions | 125 (78) | 117 (78) |
| Integration of telehealth devices with electronic health records and cloud databases | 124 (78) | 117 (78) |
| Research on suitable outcome measures for telehealth in individuals with musculoskeletal conditions | 122 (76) | 115 (77) |
| Translation, dissemination and communication developed with stakeholders | 120 (75) | 113 (75) |
| Research that examines the specific contribution of communication and information technology to the effectiveness of telehealth treatments in musculoskeletal conditions | 117 (73) | 112 (75) |
| Identification of clinician characteristics that affect response | 116 (73) | 109 (73) |
| Identification of mediators that explain or contribute to the mechanisms underpinning the effects of telehealth-delivered treatments (e.g., self-efficacy) | 116 (73) | 109 (73) |
| Standardization of telehealth-related terms and the development of frameworks for categorizing different telehealth approaches in musculoskeletal practice | 116 (73) | 108 (72) |
| New developments and advances in telehealth communication and information technologies considering predictive models and the use of artificial intelligence | 115 (72) | 109 (73) |
| **Low agreement** |  |  |
| Development of algorithms and analytical approaches for predictive models, personalized, and customized analytics, and devices to improve assessment and management of musculoskeletal conditions | 108 (68) | 103 (69) |
| Research on diagnostic tests suitable for telehealth in individuals with musculoskeletal conditions | 106 (66) | 99 (66) |
| Identify, explore, and implement the most suitable business models to support the delivery of telehealth treatment for individuals with musculoskeletal conditions | 103 (64) | 97 (65) |
| The role of organizations and advisory boards in supporting the use of evidence-based telehealth in musculoskeletal conditions | 99 (62) | 93 (62) |
| **No agreement** |  |  |
| - | - | - |
| ISC, International Steering Committee panel members; Strong agreement (> 80 %); Moderate agreement (70% - 80%); Low agreement (50% - 70%); No agreement (< 50%); ^†^ Downgrading one level of agreement; * Upgrading one level of agreement | | |

| **Supplementary file 14 B. Sensitivity analysis of the second round for the support of the research priorities in musculoskeletal pain** | | | | |
| --- | --- | --- | --- | --- |
| **Telehealth research priorities** | | **All panel members**  **(n = 133)** | **Non-ISC panel members**  **(n = 123)** |  |
| **Strong agreement** | |  |  | |
| Identification of patient characteristics that affect response to treatments delivered by telehealth | | 121 (91) | 112 (91) | |
| Qualitative telehealth research to determine perceptions, barriers, and enablers in the management of musculoskeletal conditions | | 116 (87) | 106 (86) | |
| Research that examines the specific contribution of communication and information technology and digital skills to the effectiveness of telehealth treatments in musculoskeletal conditions | | 111 (84) | 101 (82) | |
| Standardization of telehealth-related terms and the development of frameworks and guidelines for musculoskeletal telehealth practice | | 109 (82) | 100 (81) | |
| **Moderate agreement** | |  |  | |
| Investigation of patient-related safety risks and adverse events during telehealth encounters for musculoskeletal conditions | | 104 (78) | 96 (78) | |
| Research on reliability and validity testing for diagnostic tests suitable for telehealth (compared to in-person testing) in individuals with musculoskeletal conditions | | 104 (78) | 87 (71) | |
| Integration of telehealth devices with electronic health records and cloud databases | | 103 (77) | 96 (78) | |
| Identification of mediators contributing to the effects of telehealth-delivered treatments | | 103 (77) | 95 (77) | |
| Research on suitable patient-oriented research outcome measures for telehealth in individuals with musculoskeletal conditions | | 95 (71) | 87 (71) | |
| **Low agreement** | |  |  | |
| Development of algorithms and analytical approaches for predictive models, personalized, and customized analytics, and devices to improve assessment and management of musculoskeletal conditions | | 93 (70) | 86 (70) | |
| Translation, dissemination and communication developed with all parties involved* | | 93 (70) | 87 (71) | |
| The role of organizations and advisory boards in supporting the use of evidence-based telehealth in musculoskeletal conditions* | | 93 (70) | 87 (71) | |
| New developments and advances in telehealth communication and information technologies considering predictive models and the use of artificial intelligence | | 92 (69) | 85 (69) | |
| Identify, explore, and implement the most suitable business models to support the delivery of telehealth treatment for individuals with musculoskeletal conditions | | 90 (68) | 84 (68) | |
| Identification of clinician characteristics and beliefs that affect response | | 83 (62) | 78 (63) | |
| **No agreement** | |  |  | |
| - |  | - | - | |
| ISC, International Steering Committee panel members; Strong agreement (> 80 %); Moderate agreement (70% - 80%); Low agreement (50% - 70%); No agreement (< 50%); ^†^ Downgrading one level of agreement; * Upgrading one level of agreement | | | | |

| **Supplementary file 14 C. Sensitivity analysis of the third round for the support of the research priorities in musculoskeletal pain** | | |
| --- | --- | --- |
| **Telehealth research priorities** | **All panel members**  **(n = 134)** | **Non-ISC panel members**  **(n = 125)** |
| **Strong agreement** |  |  |
| Research and development of strategies for using information and communication technology to facilitate access to individuals with musculoskeletal conditions in remote or rural regions | 123 (92) | 114 (91) |
| Research on reliability and validity of clinical assessment and diagnostic tests administered via telehealth (compared to in-person testing) in individuals with musculoskeletal conditions | 118 (88) | 109 (87) |
| Identification of mediators contributing to the effects of telehealth-delivered treatments | 110 (82) | 102 (82) |
| Investigation of adverse events and patient safety during telehealth encounters for musculoskeletal conditions^†^ | 108 (81) | 99 (79) |
| **Moderate agreement** |  |  |
| Research on suitable patient-oriented research outcome measures for telehealth in individuals with musculoskeletal conditions | 106 (79) | 98 (78) |
| Translation, dissemination and communication developed with all parties involved | 101 (75) | 92 (74) |
| New developments and advances in telehealth communication and information technologies considering predictive models and the use of artificial intelligence | 99 (74) | 91 (73) |
| Data science initiative to support the use of telehealth in musculoskeletal conditions | 98 (73) | 92 (74) |
| The role of organizations and advisory boards in supporting the use of evidence-based telehealth in musculoskeletal conditions | 95 (71) | 89 (71) |
| **Low agreement** |  |  |
| Development and testing of innovative business models to support the delivery of telehealth in musculoskeletal conditions. | 89 (66) | 85 (68) |
| Identification of clinician (health professional) characteristics and beliefs that affect response to management via telehealth | 89 (66) | 83 (66) |
| **No agreement** |  |  |
| **-** | - | - |
| ISC, International Steering Committee panel members; Strong agreement (> 80 %); Moderate agreement (70% - 80%); Low agreement (50% - 70%); No agreement (< 50%); ^†^ Downgrading one level of agreement; * Upgrading one level of agreement | | |

| **Supplementary file 14 D. Sensitivity analysis of the first round for the support of the use of the term as standard terminology in musculoskeletal pain** | | | |
| --- | --- | --- | --- |
| **Telehealth terms** | **All panel members**  **(n = 160)** | **Non-ISC panel members**  **(n = 150)** |  |
| **Strong agreement** |  |  |  |
| - | - | - |  |
| **Moderate agreement** |  |  |  |
| Telehealth | 124 (78) | 117 (78) |  |
| Mobile health app (mHealth) | 122 (76) | 114 (76) |  |
| Digital health | 117 (73) | 108 (72) |  |
| Telerehabilitation | 113 (71) | 107 (71) |  |
| **Low agreement** |  |  |  |
| Telemedicine* | 111 (69) | 107 (71) |  |
| eHealth Intervention | 97 (60) | 92 (61) |  |
| Video consultations | 104 (65) | 95 (63) |  |
| Electronic health (eHealth) | 101 (63) | 96 (64) |  |
| Self Monitoring | 98 (61) | 93 (62) |  |
| Videoconferencing | 98 (61) | 91 (61) |  |
| Digital health intervention | 97 (61) | 91 (61) |  |
| Teleconsultation | 91 (57) | 87 (58) |  |
| Remote Monitoring | 83 (52) | 79 (53) |  |
| Telephone intervention | 81 (51) | 77 (51) |  |
| Telephysiotherapy | 80 (50) | 75 (50) |  |
| **No agreement** |  |  |  |
| Telemonitoring* | 78 (49) | 53 (51) |  |
| Internet- and Mobile-Based Intervention | 75 (47) | 70 (47) |  |
| Remote Patient Management (RPM) | 75 (47) | 70 (47) |  |
| Virtual care | 75 (47) | 70 (47) |  |
| Internet-based exercises | 74 (46) | 71 (47) |  |
| Tele-Intervention | 71 (44) | 69 (46) |  |
| Remote treatment or therapy | 70 (44) | 68 (45) |  |
| Telecare | 69 (43) | 67 (45) |  |
| e-consults | 67 (42) | 64 (43) |  |
| Home Telehealth | 65 (41) | 62 (41) |  |
| Tele-assistance | 63 (39) | 61 (41) |  |
| Virtual support | 63 (39) | 57 (38) |  |
| Smartphone-Based Remote Self-Management | 63 (39) | 58 (39) |  |
| Digital physical therapy practice | 60 (38) | 56 (37) |  |
| Personal videoconferencing | 60 (38) | 57 (38) |  |
| Therapist–guided synchronous (ie, live and interactive) telehealth exercise program | 60 (38) | 58 (39) |  |
| Video visits | 59 (37) | 56 (37) |  |
| Remote or non–face-to-face care models | 57 (36) | 54 (36) |  |
| Telecoaching | 54 (34) | 52 (35) |  |
| Telementoring | 54 (34) | 53 (35) |  |
| Digital therapeutics (DTx) | 50 (31) | 48 (32) |  |
| e-visits | 43 (27) | 42 (28) |  |
| ISC, International Steering Committee panel members; Strong agreement (> 80 %); Moderate agreement (70% - 80%); Low agreement (50% - 70%); No agreement (< 50%); ^†^ Downgrading one level of agreement; * Upgrading one level of agreement | | |  |

| **Supplementary file 14 E. Sensitivity analysis of the second round for the support of the use of the term as standard terminology in musculoskeletal pain** | | |
| --- | --- | --- |
| **Telehealth terms** | **All panel members**  **(n = 133)** | **Non-ISC panel members**  **(n = 123)** |
| **Strong agreement** |  |  |
| Digital health | 112 (84) | 104 (85) |
| Telehealth^†^ | 109 (82) | 99 (80) |
| **Moderate agreement** |  |  |
| Mobile health app (mHealth) | 98 (74) | 89 (72) |
| Digital health intervention | 98 (74) | 91 (74) |
| Online consultations | 95 (71) | 91 (74) |
| Telemedicine | 94 (71) | 88 (72) |
| Teleconsultation | 94 (71) | 86 (70) |
| **Low agreement** |  |  |
| Video consultations | 86 (65) | 63 (51) |
| Electronic health (eHealth) | 85 (64) | 76 (62) |
| eHealth Intervention | 85 (64) | 77 (63) |
| Telerehabilitation | 84 (63) | 76 (62) |
| Digital rehabilitation | 84 (63) | 78 (63) |
| Online healthcare | 83 (62) | 79 (64) |
| Remote monitoring | 75 (56) | 69 (56) |
| Remote home-based exercise program | 73 (55) | 70 (57) |
| Online pain management | 72 (54) | 70 (57) |
| Videoconferencing | 67 (50) | 63 (51) |
| **No agreement** |  |  |
| Self-monitoring* | 66 (50) | 65 (53) |
| Virtual consultations* | 65 (49) | 63 (51) |
| Virtual appointment* | 63 (47) | 62 (50) |
| Digital care program | 63 (47) | 58 (47) |
| Digital Health Application (DiHA) | 62 (47) | 56 (46) |
| Remote self-management support | 59 (44) | 55 (45) |
| Tele-assessment | 58 (44) | 55 (45) |
| Telephysiotherapy | 57 (43) | 53 (43) |
| Video assessments and treatment | 56 (42) | 52 (42) |
| Virtual clinic | 56 (42) | 53 (43) |
| Telephone assessments and treatment | 55 (41) | 53 (43) |
| Virtual exercise prescription | 53 (40) | 51 (41) |
| Telephone intervention | 53 (40) | 49 (40) |
| Technology-based intervention | 52 (39) | 50 (41) |
| Virtual Physiotherapy | 50 (38) | 48 (39) |
| Remotely delivered treatment | 49 (37) | 45 (37) |
| Virtual reality, Augmented reality & Extended reality (VR, AR & XR) | 49 (37) | 48 (39) |
| Digital tool | 40 (30) | 37 (30) |
| Telepractice | 37 (29) | 34 (28) |
| Virtual reality immersive/ non immersive | 36 (27) | 36 (29) |
| Virtual reality asynchronous/synchronous | 36 (27) | 35 (28) |
| Blended care | 32 (24) | 29 (24) |
| Virtual physical assessment and provisional Tele diagonosis | 28 (21) | 28 (23) |
| Distance physiotherapy | 26 (20) | 24 (20) |
| Remote presence | 15 (11) | 14 (11) |
| Audio-simultaneous rehabilitation | 7 (5) | 7 (6) |
| ISC, International Steering Committee panel members; Strong agreement (> 80 %); Moderate agreement (70% - 80%); Low agreement (50% - 70%); No agreement (< 50%); ^†^ Downgrading one level of agreement; * Upgrading one level of agreement | | |

| **Supplementary file 14 F. Sensitivity analysis of the third round for the support of the use of the term as standard terminology in musculoskeletal pain** | | |
| --- | --- | --- |
| **Telehealth terms** | **All panel members**  **(n = 134)** | **Non-ISC panel members**  **(n = 125)** |
| **Strong agreement** |  |  |
| - | - | - |
| **Moderate agreement** |  |  |
| Digital health intervention | 100 (75) | 92 (74) |
| Telemedicine | 96 (72) | 89 (71) |
| Telerehabilitation | 94 (70) | 87 (70) |
| **Low agreement** |  |  |
| Online consultations | 90 (67) | 82 (66) |
| Mobile health app (mHealth) | 90 (67) | 82 (66) |
| Electronic health (eHealth) | 89 (66) | 83 (66) |
| Remote monitoring | 80 (60) | 74 (59) |
| Videoconferencing | 77 (58) | 72 (58) |
| Video consultations | 77 (58) | 70 (56) |
| eHealth Intervention^†^ | 77 (57) | 61 (49) |
| Teleconsultation | 69 (52) | 63 (50) |
| Online pain management^†^ | 67 (50) | 56 (45) |
| Online healthcare | 67 (50) | 62 (50) |
| **No agreement** |  |  |
| Remote home-based exercise program | 62 (46) | 59 (47) |
| Digital rehabilitation | 54 (40) | 49 (39) |
| Digital health rehabilitation | 54 (40) | 51 (41) |
| Asynchronous Care Delivery Platform | 22 (16) | 20 (16) |
| ISC, International Steering Committee panel members; Strong agreement (> 80 %); Moderate agreement (70% - 80%); Low agreement (50% - 70%); No agreement (< 50%); ^†^ Downgrading one level of agreement; * Upgrading one level of agreement | | |
